# Medical and Nonmedical Applications of Synthetic Transmembrane Anion Transporters

**DOI:** 10.1021/acs.chemrev.5c00129

**Published:** 2025-07-07

**Authors:** Uththara M. C. Rathnaweera, Surid Mohammad Chowdhury, Rayhanus Salam, Nathalie Busschaert

**Affiliations:** Department of Chemistry, 5783 Tulane University, New Orleans, Louisiana 70118, United States

## Abstract

The transport of anionic species across phospholipid bilayers is an important biological function that plays a vital role in cellular and organismal homeostasis. The function is normally performed by transmembrane transport proteins, but several diseases have been linked to malfunctioning or malregulation of these proteins (so-called “channelopathies”). As a consequence, supramolecular chemists have tried to develop synthetic molecules that can transport anions across biological membranes. While the original goal of this endeavor was the development of potential therapeutics for channelopathies, it is now clear that there are several other potential medical and nonmedical applications for synthetic transmembrane anion transporters. The medical applications of synthetic anion transporters have been widely explored, especially their potential as anticancer agents. However, there are many other applications of synthetic anion transporters that are often overlooked. These range from antibacterial activity and drug delivery, to nonmedical applications such as biomimicry, the development of optical sensors, catalysis, organic synthesis and others. In this review, we aim to highlight the versatility of synthetic transmembrane anion transporters and to convince chemists to look beyond channelopathies and anticancer activity as applications of artificial anion transporters.

## Graphical abstract

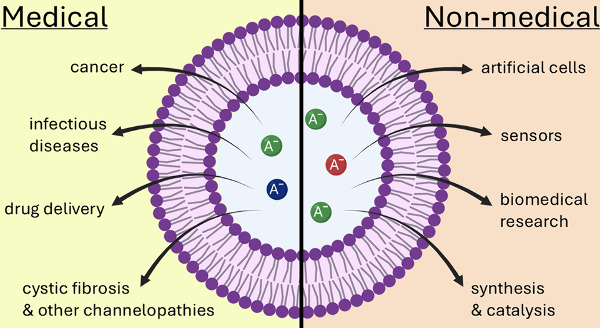

## 1 Introduction

Cellular membranes consist of a lipid bilayer that provides a hydrophobic barrier between the intracellular and extracellular environment or organelle. This barrier is made semipermeable by various embedded transport proteins that maintain ion gradients, import essential nutrients, and export cellular waste or toxic compounds; thereby playing a crucial role in cellular and organismal homeostasis.^1^ It is estimated that nearly 5–10% of all human genes code for transport-related proteins,^2,3^ which demonstrates the importance of transmembrane transport in biology. Unfortunately, there are many diseases that have been linked to the malfunctioning of various transport proteins. These diseases are often called ‘channelopathies’,^4^ although there are also many pathologies caused by mutated transport proteins that do not belong to the ion channel superfamily (but instead belong to the ABC (ATP-binding cassette) or SLC (solute carrier) superfamilies).^5–7^ The best-known example is cystic fibrosis, a genetic disease caused by mutations in the CFTR protein (cystic fibrosis transmembrane conductance regulator).^8^ The discovery that CFTR functions as a channel for Cl^–^ (and other small anions such as HCO_3_^–^),^9,10^ encouraged supramolecular chemists to develop synthetic molecules that can transport these anions across biological membranes.

As shown in Figure 1a, synthetic transmembrane transporters and transport proteins generally translocate anions across lipid bilayers via one of two mechanisms: (1) ion channel—membrane-spanning molecules or self-assemblies that provide a hydrophilic path in the membrane through which the anion can diffuse, or (2) mobile carrier—molecules that form a supramolecular complex with the anion which subsequently diffuses through the membrane.^11–16^ For synthetic transporters, researchers have designed molecules that can function through more exotic mechanisms, such as relays,^17,18^ Jacob’s ladder,^19^ and molecular machines.^20^ Artificial anionophores can transport anions via an electroneutral or electrogenic mechanism (Figure 1b). In electroneutral transport, the charge imbalance that would occur due to anion transport is compensated by the transport of another anion in the opposite direction (antiport), or the transport of a cation in the same direction (symport) (in a pure electroneutral mechanism, these two transport processes cannot be separated). On the other hand, electrogenic anion transport allows uniport of an anion across a membrane, thereby creating a membrane potential. This membrane potential eventually hinders further transport of anions, but is often compensated by a separate uniport event caused by the same anionophore, or by the presence of other ionophores or transport proteins in the membrane.

**Figure 1:**
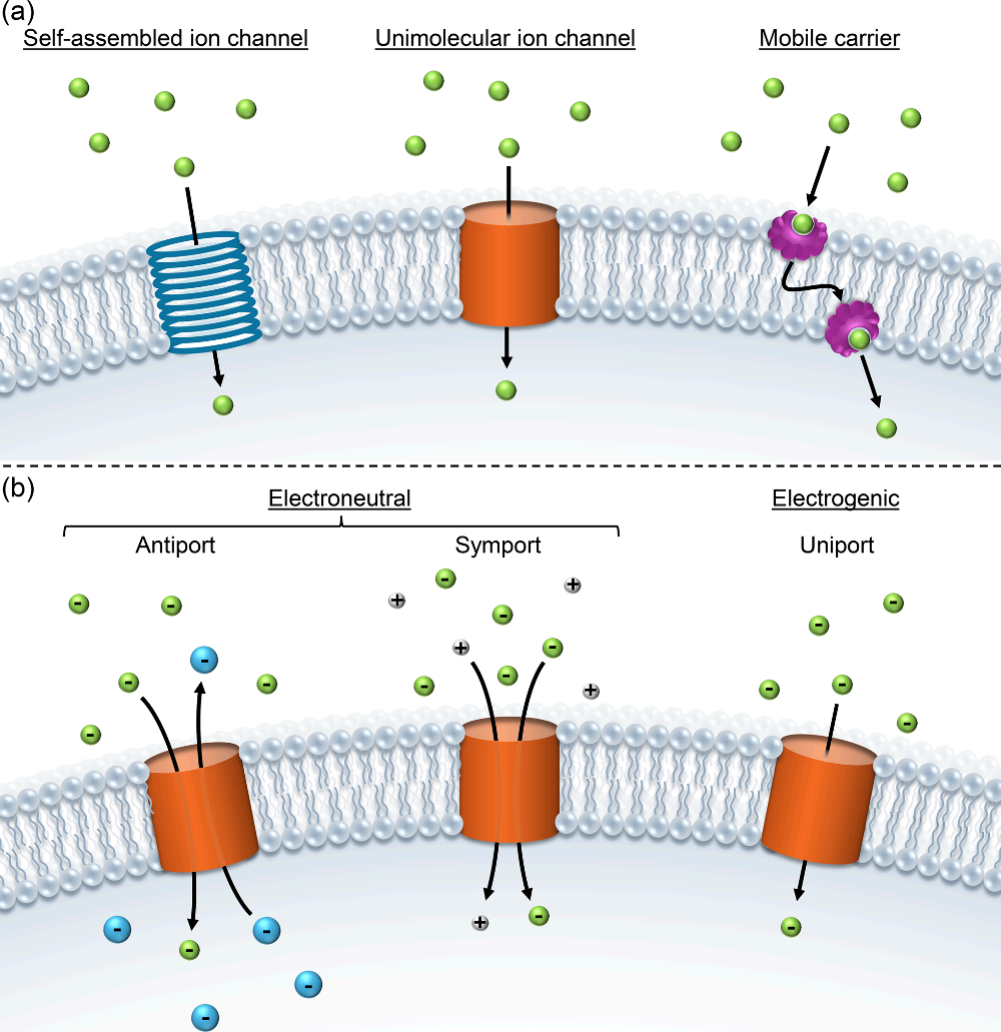
Ion transporters are classified by their mechanism: (a) channel or mobile carrier, (b) electrogenic uniport or electroneutral antiport/symport (overall antiport or symport can also be the result of two independent uniport events, but the figure shows an electroneutral process where they are inseparably linked).

Although the original aim for the development of artificial transmembrane anion transporters (or anionophores) was to find a treatment for cystic fibrosis, most studies have focused on liposome-based assays that provide proof-of-concept that synthetic molecules can transport anions, with little focus on the application of such transporters. Nonetheless, these studies have provided many useful insights and guidelines. For example, the liposome-based studies have led to the insight that both lipophilicity (or more accurately lipophilic balance)^21^ and anion binding ability are important parameters to be optimized, and to a lesser extent the size of the transporter.^22–24^ When transporters are too hydrophilic they are unable to partition into the membrane, whereas if they are too lipophilic they are unable to move to the interface to pick up an anion or have deliverability problems.^25^ Similarly, when transporters bind their target ion too weakly they cannot extract it from the aqueous layer, but if they bind too strongly they cannot release their substrate on the other side of the membrane.^26,27^ As a result, the −CF_3_ group has become one of the most popular substituents to optimize anionophores due to its favorable effect on both lipophilicity and anion binding.^28^ Lessons have also been learned in terms of substrate selectivity. For synthetic channels and pore-forming molecules, selectivity is largely determined by the (partial) charge on the inside of the channel (anion vs cation selectivity) and size match (i.e., ions with the correct size pass through easiest, and larger ions are rejected).^29–31^ For synthetic mobile carriers, selectivity is often determined by the Hofmeister series, whereby more lipophilic anions are easier to transport than more hydrophilic ions.^32,33^ A number of groups have been able to break this Hofmeister bias and transport more hydrophilic ions selectively over more hydrophobic anions (usually through the use of a macrocycle for improved binding, or the use of halogen bonding).^34–37^ Recently, it has also become clear that interactions of artificial anion transporters with the phospholipid headgroups of the membrane lipids need to be considered.^38–40^ Due to the anionic nature of the phosphate moiety in biological lipids, many synthetic anion transporters bind to the lipid headgroup, which competes with the anion transport process. Given the recent findings that simple anion receptors can bind to phospholipid headgroups,^41–44^ this avenue should be explored further in the future.

While more fundamental studies are still necessary, especially regarding anion selectivity, we are now in a position where we can explore the applications of synthetic anion transporters in more detail. Cation transporters (cationophores) have been around for much longer and have therefore found several commercial applications such as ion selective electrodes (valinomycin),^45^ and animal feed antibiotics (monensin, salinomycin).^46,47^ The field of anion transporters is much younger and has not seen a commercial application yet. However, recent years have seen an explosion in the number of publications where the potential cystic fibrosis or anticancer activity of synthetic anion transporters has been investigated, as can be seen by the many reviews regarding the biological applications of anion transporters.^48–58^ Unfortunately, other potential medical applications of synthetic anion transporters are often overlooked (e.g., antibacterial activity and drug delivery). In addition, it is underappreciated that the unique combination of a hydrophobic barrier made semipermeable by a synthetic transporter offers the opportunity for many nonmedical applications (e.g., optical sensors and catalysis). In this review, we therefore aim to highlight the versatile applications that are possible with artificial transmembrane anion transporters and to inspire other researchers to consider these alternative applications for their anionophores. We will use the terms synthetic anion transporter, artificial anion transporter and anionophore throughout this review as synonyms for any compound that was designed to facilitate the movement of anionic species across membranes. While we tried to be as thorough as possible, no review can ever be fully comprehensive, and examples have been chosen to best demonstrate the progress in the field up to 2024.

## 2 Medical Applications

### 2.1 Cystic Fibrosis

Cystic fibrosis (CF) is one of the most prevalent rare genetic diseases among Caucasians and is caused by mutations in the CFTR gene that ultimately lead to a shorter life expectancy.^59^ More than 2000 CFTR mutations are known to date, of which >340 are classified as causing CF (the most common of which is the ΔF508 mutation).^60^ Mutations can lead to absence or reduced expression of the CFTR protein, improper trafficking of the CFTR protein to the membrane, or reduced CFTR channel activity or stability.^61^ CFTR is responsible for anion transport (chloride and bicarbonate) at the apical membranes of epithelial cells and thereby also plays a role in regulating fluid secretion and luminal pH.^62^ Most CF symptoms are thus manifested in epithelial tissues, with the most serious effects seen in the lungs (which is usually the ultimate cause of death).^63^ The relative high prevalence of CF in rich countries has led to significant efforts in trying to find a treatment for this disease. As a result, the past decade has seen the emergence and FDA approval of CFTR modulators that can treat the underlying cause of CF by either improving CFTR protein folding (correctors) or by increasing the ‘open’ probability of the CFTR protein (potentiators): ivacaftor (a potentiator that can increase the opening probability of CFTR),^64^ lumacaftor (a corrector that stabilizes misfolded ΔF508 CFTR),^65^ tezacaftor (a corrector that improves processing and trafficking of CFTR to the cell membrane)^66^ and elexacaftor (a corrector that facilitates trafficking of CFTR to the cell surface).^66^ While these drugs have presented a major breakthrough in the treatment of CF (especially the combination treatments), they each only function on a subset of CFTR mutations. Furthermore, correctors and potentiators do not work in patients with nonsense mutations that lead to the absence of the CFTR protein and an estimated 10–15% of CF patients therefore still have no effective drug treatment.^67^ There is therefore still a need to develop compounds that can treat the remaining CF patients. One possibility is the development of artificial anion transporters that can mimic the function of native CFTR and restore epithelial homeostasis. Although CFTR belongs to the family of ABC transporters which normally hydrolyze ATP to transport substrates against their electrochemical gradient,^68^ CFTR is an exception and functions as a ligand-gated ion channel that allows passive diffusion of Cl^–^.^69,70^ The development of small-molecule anionophores for the treatment of CF should thus be a feasible task. Here, we will focus only on compounds that have shown chloride transport ability across epithelial cell lines or in animal studies.

The first attempts at preparing synthetic chloride transporters for the treatment of cystic fibrosis were based on mimicking peptide sequences from natural chloride channels. Tomich and colleague have studied in detail the chloride transport activity of synthetic peptides based on the second membrane-spanning region of the α-subunit of the postsynaptic glycine receptor (GlyR).^71,72^ Their best results have been obtained with a 23 amino acid transmembrane peptide sequence appended with four lysine residues at the C- or N-terminus for solubility purposes (peptide **1** and **2**, Scheme 1).^73^ Planar lipid bilayer studies and whole-cell patch clamping revealed that these peptides function as anion-selective channels.^74,75^ They have also shown that **1** and **2** can increase the short-circuit current (*I*_SC_), hyperpolarize the transepithelial potential difference (*V*_te_), decrease the transepithelial electrical resistance (*R*_te_), and induce fluid secretion in a number of mammalian epithelial cells, such as MDCK cells (Madin-Darby canine kidney), and T84 cells (human colonic adenocarcinoma cells).^76^ These effects were not seen in the absence of extracellular Cl^–^, indicating that chloride transport plays a role. In addition, the effects of peptides **1** and **2** on epithelial cells were amplified when the cells were treated with activators of K^+^ channels (e.g., 1-EBIO), presumably because the K^+^ channels maintain the electrochemical driving force for Cl^–^ efflux induced by **1** and **2**.^77^ The combination of peptide **2** and a K^+^ channel activator was also found to increase chloride secretion in CFT1 cells (human CF airway epithelial cells), which in turn stimulates glutathione efflux from this cell line.^78^ This finding is significant because glutathione is a major antioxidant in lung epithelium fluid and is reduced in CF patients. In later work, Tomich and co-workers tried to shorten the peptide sequence of the ion channels.^79,80^ While these modified peptides were still potent channels, they lost their anion-over-cation selectivity and affected paracellular pathways in epithelial cells, making these newer peptides less suitable for CF treatment (but potentially suitable for drug delivery–see section 2.5).^81,82^

**Scheme 1:**
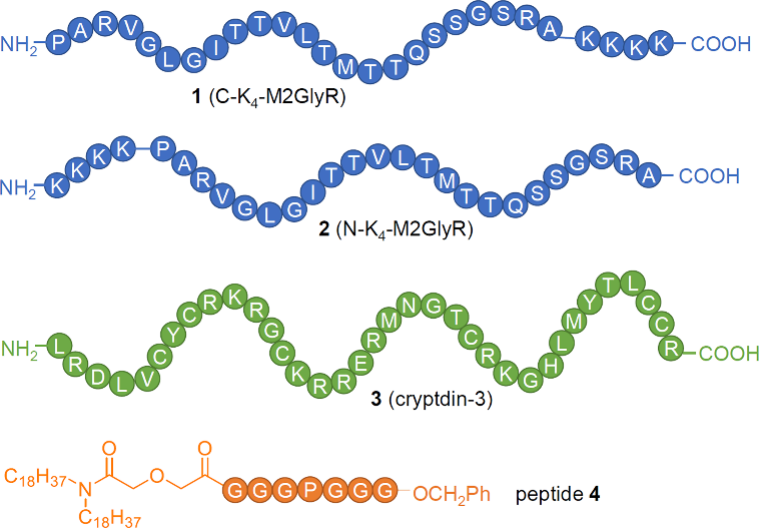
Structure of Peptide-Based Chloride Channels **1**–**4**; Amino Acids Are Represented by Their Single Letter Abbreviation

Other peptide-based anion channels have also been tested in epithelial cells. Lencer et al. found that **cryptdin-3** (a type of antimicrobial peptide, see Scheme 1) could stimulate Cl^–^ secretion in T84 cell monolayers, as measured by the increase in *I*_SC_.^83^ Similar to Tomich’s peptides **1** and **2**, the activity of **cryptdin-3** was diminished in the absence of extracellular chloride, and could be enhanced by the addition of K^+^ channel activators. Gokel and colleagues have reported a synthetic amphiphilic peptide (**4**, Scheme 1) that was shown to transport chloride anions in liposomes and planar lipid bilayer studies.^84^ Using the Ussing chamber technique with primary epithelial layers differentiated from murine tracheal epithelia, they showed that **4** can induce a change in the transepithelial voltage (*V*_te_) under normal growth conditions, but not when chloride-free buffer is used.^85^ This demonstrates that **4** also functions as a Cl^–^ channel in epithelial cells, and has potential for CF treatment.

A variety of nonpeptide-based ion channels have also been reported as potential CF therapeutics. Jeffrey Davis and co-workers showed that calix[4]­arene **5** (Scheme 2) mediates H^+^/Cl^–^ cotransport across liposomes, forms ion channels in voltage-clamp assays with black lipid membranes (BLM), and causes a current across the cell membrane of HEK-193 cells (human embryonic kidney cells) only when the cells are suspended in NaCl buffer.^86^ Yang and colleagues studied in depth the anion transport and biological activity of isophthalamide analogue **6** (Scheme 2).^87^ Chloride transport induced by **6** was observed using the Cl^–^-sensitive fluorophore SPQ in both liposomes and MDCK cells, while patch-clamp experiments with giant liposomes suggested that **6** self-assembles into an anion-selective channel. Furthermore, they showed that **6** is also able to increase Cl^–^ conductance in CuFi-8 cell lines (cells derived from human cystic fibrosis bronchial epithelia) and that it did not induce an immune response or other toxicity in mice.^88^ Later, Yang reported an analogous compound which displays chloride and bicarbonate transport in liposomes, as well as in Calu-3 and CFBE41o monolayers (two epithelial cell lines that display functional CFTR or ΔF508-mutated CFTR, respectively).^89^ The group of Talukdar have shown that other isophthalic acid derivatives (**7** and **8**, Scheme 2) also form chloride-selective self-assembled ion channels with biological activity, confirming the power of the isophthalamide scaffold.^90^ Compounds **7** and **8** were able to increase the intracellular Cl^–^ concentration in human epithelial cells (MCF-10A) and did not show cytotoxicity to this and other cell lines at concentrations as high as 100 μM. Away from the isophthalamide scaffold, Muraglia et al. showed that amphotericin B, a known antifungal agent that can form unselective ion channels in membranes (see section 2.4.2), can stimulate HCO_3_^–^ secretion, and restore airway surface liquid pH, viscosity and antibacterial activity in cultured and primary airway epithelia from people with CF.^91^ They also showed that amphotericin B (administered as the FDA-approved AmBisome formulation) can increase the airway surface liquid pH in pig models of CF, providing one of the few examples where synthetic ionophores have been tested *in vivo* against CF.

**Scheme 2:**
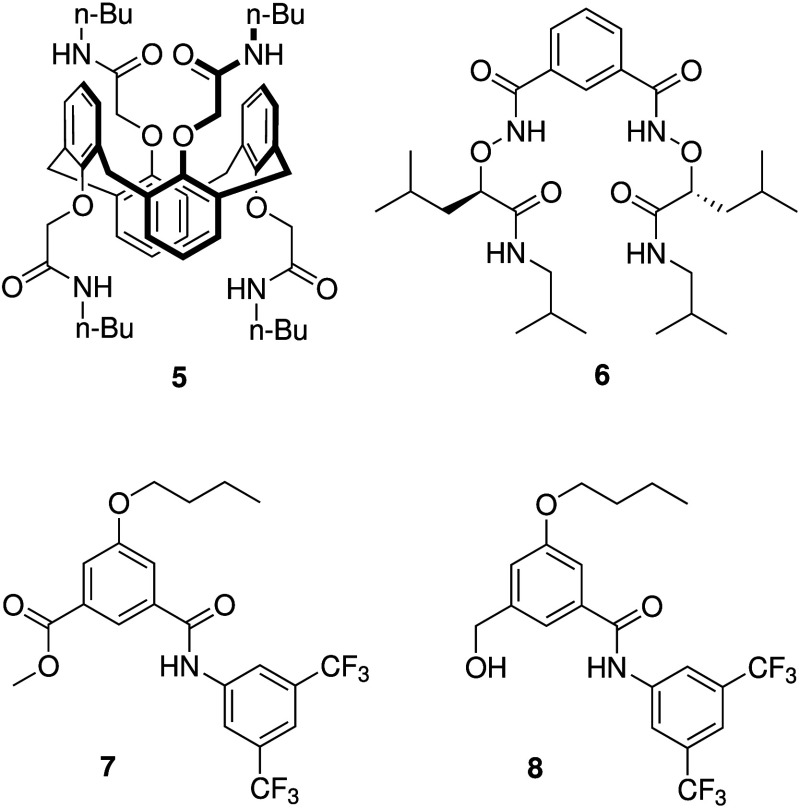
Structure of Nonpeptide-Based Ion Channels **5**–**8**

In the past decade, there has been a surge of small-molecule chloride transporters that function as mobile carriers. Early work had shown that 5,10,15,20-tetraphenyl-21*H*,23*H*-porphine manganese­(III) chloride^92^ and squalamine analogues^93^ can increase anion permeability in various animal and human epithelial cells. Later, the group of Anthony Davis intensively studied the anion transport capabilities of a variety of highly lipophilic neutral anion receptors.^94,95^ In their earliest work, they had already shown that cholapod **9** (Scheme 3) is able to transport chloride anions in liposomes and across live cells using MDCK epithelia in the Ussing chamber technique.^96^ More recently, the group developed a high-throughput method to assess the chloride transport ability of synthetic transporters in epithelial cells using Fischer Rat Thyroid (FRT) cells expressing a mutant yellow fluorescent protein (YFT-H148Q/I152L) developed by Verkman and co-workers that can detect changes in intracellular halide concentrations.^97–99^ Using this method, they identified compounds **10–13** (Scheme 3) as potential leads. Transport ability was further confirmed using the CF bronchial epithelial cell line CFBE41o^–^ and FRT cells expressing ΔF508-mutated CFTR. The anionophore activity of **10**–**13** was also found to be additive to the effect of the CF-drug ivacaftor. Furthermore, the compounds were found to be nontoxic at concentrations that mediate anion transport, rendering them potential candidates for CF treatment.

**Scheme 3:**
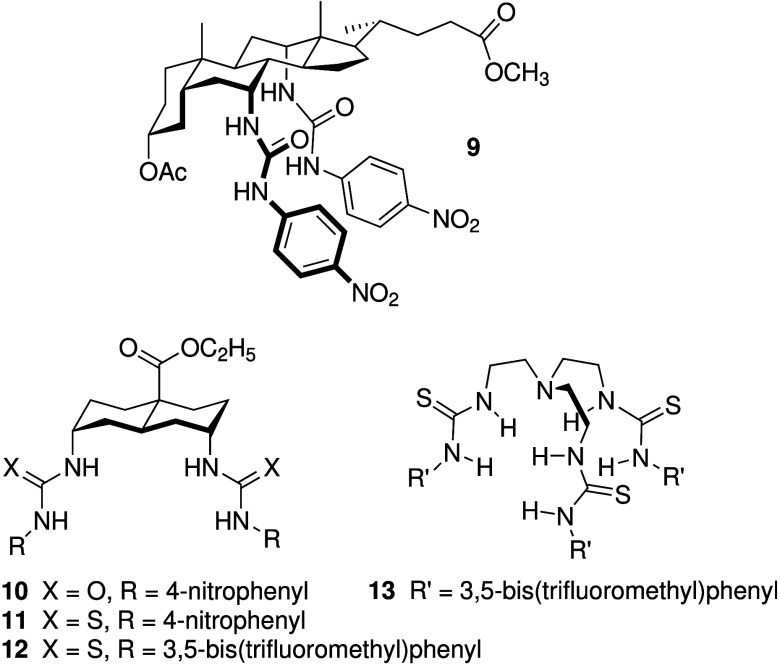
Structure of Anion Carriers **9-13** Reported by Davis and Co-workers

Prodigiosins and tambjamines are two classes of natural products that have been studied in depth for their anion transport properties. While they are best known for their anticancer properties (see next section), Quesada and co-workers have recently shown that they also have potential as cystic fibrosis treatments due to their ability to transport Cl^–^ and HCO_3_^–^.^100–104^ It was found that prodigiosin, certain tambjamines (e.g., **14**, Scheme 4) and derivatives of prodigiosin and tambjamines where one of the pyrrole groups was replaced by a 1,2,3-triazole (e.g., **15**, Scheme 4) could facilitate anion transport in epithelial cells at concentrations below their cytotoxicity levels, and that this effect does not interfere with CFTR modulators. Furthermore, assays with **14** in bronchial epithelial cell monolayers derived from CF (ΔF508/ΔF508 genotype) and non-CF individuals showed that these compounds can restore some of the impaired properties of the airway surface liquid of CF epithelia (reduce fluid reabsorption, correct pH, and reduce viscosity of the mucus). Talukdar and co-workers also showed that triazoles are a good motive to incorporate into anion transporters.^105^ They replaced one amide in isophthalamide derivatives with a triazole, and found that these compounds function as anion transporters in liposomes and cancer cells, but had low cytotoxicity (making them more suitable for CF treatment than anticancer activity).

**Scheme 4:**
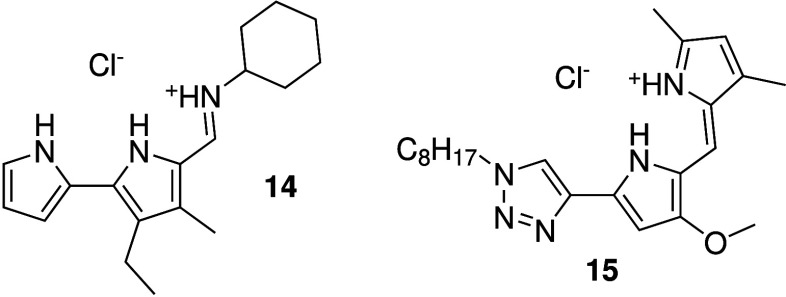
Structure of Anion Carriers **14**–**15** Reported by Quesada and Co-workers

A recent example of a mobile carrier with potential for CF treatment is the pentaamide macrocycle **16** reported by Cao et al. (Scheme 5).^106^ The pentaamide was shown to form strong 1:1 complexes with halides and oxoanions in solution, and could transport anions across POPC liposomes with a high selectivity for Cl^–^. Furthermore, treatment of cultured human bronchial epithelial (HBE) cells isolated from CF patients with **16** showed a >50% increase in the thickness of the airway surface liquid, suggesting that **16** can increase hydration of this layer presumably due to its ability to restore Cl^–^ transport in the cells.

**Scheme 5:**
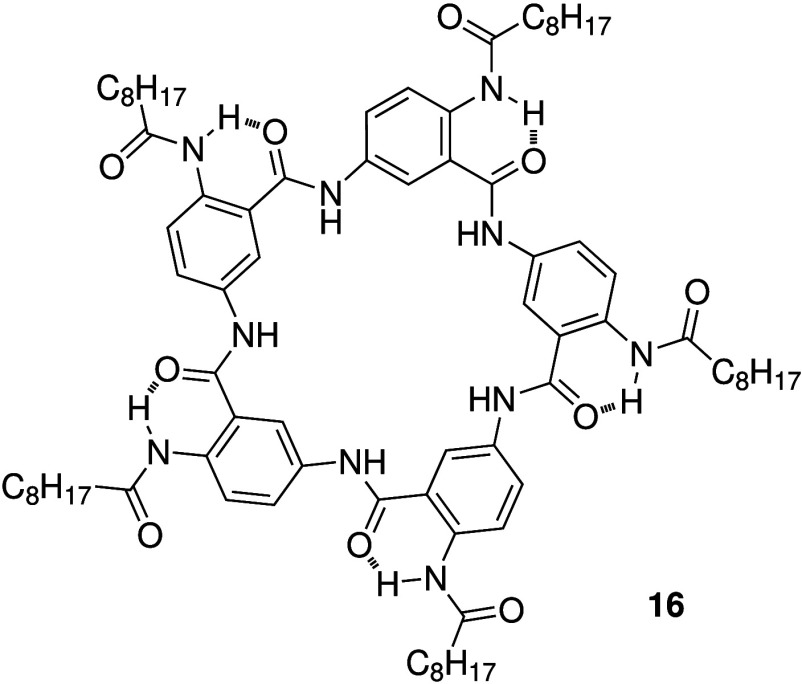
Structure of Pentaamide Anion Carrier **16**

While the aforementioned studies show the potential of anionophores for the treatment of CF (most likely in combination therapies with CFTR modulators), one of the biggest challenges associated with the development of synthetic transporters is deliverability. Because the mode of action requires anionophores to be located in the membrane, they are inherently hydrophobic. This reduces their aqueous solubility and therefore their pharmacokinetic properties and drug-likeness. To overcome this problem, Gale and co-workers developed simple urea-based transporters appended with adamantane to form water-soluble inclusion complexes with β-cyclodextrin.^107^ Although the inclusion complexes were still able to transport anions in liposomes, the biological activity of the delivery system has not yet been tested. Similarly, Quesada and co-workers have shown that lipophilic click-tambjamines (analogs of compound **15**) can be efficiently delivered to liposomes by encapsulating the tambjamines in nanostructured lipid carriers (NLCs), the biological activity of this delivery system also remains to be investigated.^108^ One of the few studies of improved delivery in biological systems has been reported by the group of Anthony Davis.^109^ They tried to overcome the deliverability issue of their highly hydrophobic *cis*-decalin based transporters (e.g., **10**–**12**). Previous studies had already shown that pure POPC liposomes are insufficient for the delivery of the anionophores. However, when the liposomes were functionalized with an amphiphilic peptide, and the receiving membrane was also treated with a complementary amphiphilic peptide, membrane fusion and transporter delivery could be achieved upon the formation of a coiled-coil between the two peptides. The delivery system was successful in both liposomes and Fischer Rat Thyroid cells. However, because the receiving membrane needs modification, it is not the most practical delivery system. Comparably, Jeong and co-workers developed water-soluble procarriers which can be converted to active chloride transporters in liposomes and in Fischer rat thyroid cells upon the addition of the appropriate enzyme (e.g., **17**, Scheme 6).^110^ However, the enzymes need to be added externally to the cell culture, and the system is therefore not perfect for real-life applications. Additional work on anion transporter delivery is therefore still needed before these compounds can be used in medical applications.

**Scheme 6:**
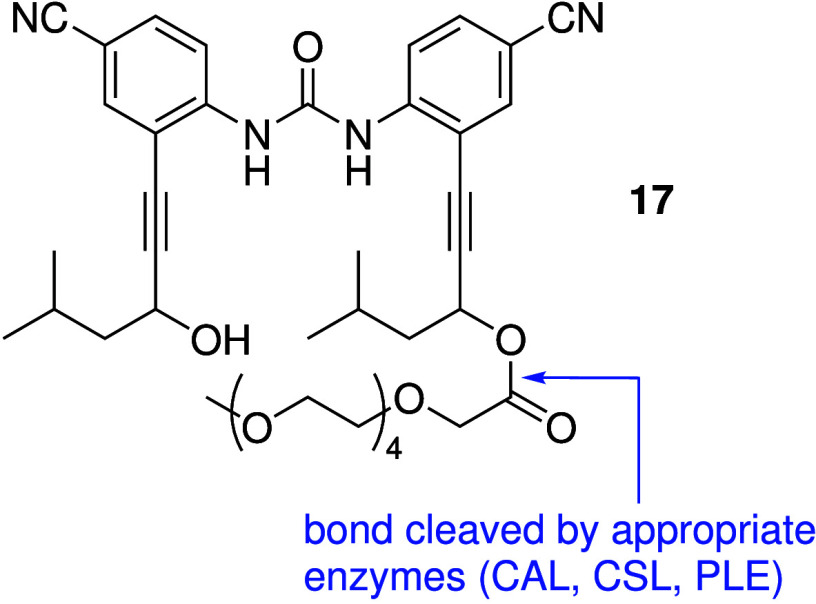
Water-Soluble Pro-carrier **17** Reported by Jeong and Co-workers

### 2.2 Other Channelopathies

Cystic fibrosis is the most common channelopathy related to anion transport. However, there are many more rare diseases that have been linked to malfunctioning transport proteins, most of which have no known treatment. For example, Bartter Syndrome^111^ and Dent’s disease^112^ are two other channelopathies related to malfunctioning Cl^–^ transport, but they are due to mutations in proteins other than CFTR (a variety of mutations have been linked to these diseases). However, it is also important to note that many channelopathies are not related to malfunctioning chloride transport, but due to malfunctioning of transporters for other ions and substrates (for an overview, see reviews by Giacomini and colleagues,^6^ and Schaller and Lauschke^7^). For example, more than 80 SLC transporters have been linked to rare genetic diseases,^7^ including Pendred syndrome (mutations in pendrin (SLC26A4 gene), an anion exchange protein for Cl^–^, I^–^ and HCO_3_^−^)^112^ and hereditary hypophosphatemic rickets with hypercalciuria (HHRH) (loss-of-function mutations in the sodium-phosphate cotransporter NPT2c (SLC34A3 gene)).^113^ Most SLC transporters function as passive or secondary active transporters,^114^ and their function should therefore be easy to mimic using small-molecule transmembrane ion transporters. Furthermore, there is increasing evidence that transport protein malfunction also plays a role in more common complex diseases such as cancer and diabetes.^114,115^ For example, the glucose transporters SLC5A1/SGLT1 and SLC5A2/SGLT2,^116,117^ as well as the zinc transport protein SLC30A8,^118,119^ are closely linked to type 1 and type 2 diabetes. Due to the different metabolic needs of cancer cells, many important transport proteins are up- or down-regulated in cancer, making them useful therapeutic targets.^114^ In addition, a variety of neurological disorders have been linked to imbalanced neurotransmitter transport. For example, Alzheimer disease, amyotrophic lateral sclerosis (ALS) and Parkinson’s disease have all been associated with downregulated levels of EAAT2, a glutamate transport protein.^120–124^

In order to develop treatments for these diseases using artificial ionophores, it is necessary to develop transporters that can transport anions other than Cl^–^ and HCO_3_^–^ (preferably selectively). In the last decades, there have been a few reports of synthetic transmembrane transporters that can transport other anions, such as I^–^,^125,126^ F^–^,^127–131^ SO_4_^2–^ and other oxyanions,^132–137^ and amino acids,^138–142^ but most have not been tested in a biological environment. Notable exceptions are the iodide transporter reported by Roh and Kim,^125^ fluoride transporter **18** by Gabbaï and colleagues,^129^ and proline transporter **19** by Ballester and co-workers (Scheme 7).^142^ Roh and Kim showed that a hydrophobic porphyrin-based organic cage was able to selectively transport iodide anions across the membranes of synthetic vesicles and human embryonic kidney cells (HEK-293T). Lipophilic pnictogen cation **18** was shown to transport F^–^, Cl^–^ and OH^–^ in synthetic liposomes, and can induce hemolysis of human red blood cells in the presence of fluoride (presumably due to F^–^ transport). The calixpyrrole-derivative **19** can selectively transport the amino acid proline in liposomes and in HeLa cells at high concentrations. While these examples show that the transport of anions other than chloride in biological systems is possible, none of these synthetic transporters have been tested in therapeutically relevant systems. In order to find treatments for other channelopathies, it will be necessary to further develop artificial anionophores for a variety of anions and to test them in relevant model systems.

**Scheme 7:**
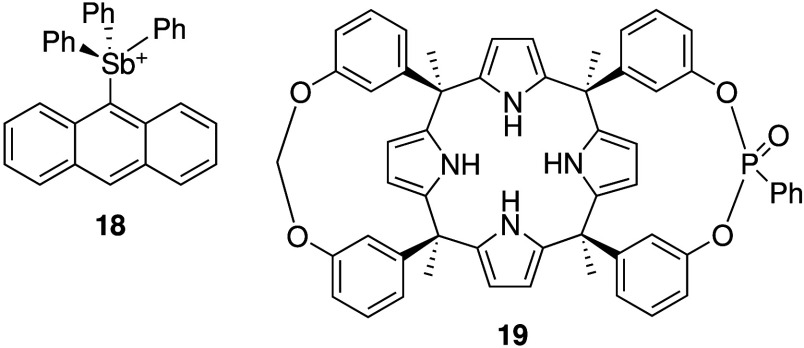
Structure of Fluoride Transporter **18** and Proline Transporter **19**

### 2.3 Cancer

Maintaining cellular ion concentrations is essential for cell survival. Therefore, the disruption of this homeostasis by artificial transporters could lead to cytotoxicity. This is beneficial in cases where cell death is the desired outcome, such as anticancer activity. Artificial transmembrane ion transporters have potential in this regard because many cancer cells have altered ion and pH concentrations and have different expression levels of transporting proteins.^143–147^ Furthermore, the development of drug resistance against ionophores might be difficult because the process is not dependent on binding to proteins that can be easily mutated. As a result, researchers have recently become interested in the anticancer activity of artificial anionophores (as well as cationophores^148^).

The first report of the link between transmembrane anion transport and cancer came from Sessler and co-workers.^149^ Prodigiosin (**20a**, Scheme 8), a natural product found in certain *Serratia* and *Streptomyces* species, was already known as an anticancer agent and early work by Ohkuma and co-workers had suggested that it can transport H^+^ and Cl^–^ across liposomes and inhibit lysosomal acidification in cells (prodigiosin is only able to bind Cl^–^ strongly upon protonation).^150,151^ Sessler et al. subsequently confirmed that prodigiosin **20a** and synthetic analogues (e.g., **20b**, Scheme 8) are able to facilitate H^+^/Cl^–^ transport in liposomes, and that the observed transport activity correlated well with the anticancer activity of the prodigiosins in A549 and PC3 cancer cell lines. Later, Quesada and co-workers showed that the synthetic prodigiosin analogue Obatoclax (**21**, Scheme 8), a compound that has undergone several clinical trials for its anticancer potential,^152–156^ is also able to function as a potent anionophore for Cl^–^, NO_3_^–^ and HCO_3_^–^.^157^ Furthermore, Obatoclax was shown to display cytotoxicity against the GLC4 cancer cell line through a mechanism that involves dissipation of lysosomal pH (measured using the pH-sensitive dye Acridine Orange),^158,159^ and to have antimetastatic activity in melanoma *in vitro* and *in vivo*.^160^ While prodigiosin and Obatoclax clearly have anion transport ability in liposomes, various mechanisms for their anticancer activity have been suggested that are unrelated to their ionophore activity, such as inhibition of mTOR complexes,^159^ binding to the BH3 domain of BCl-2 proteins,^161^ DNA cleavage,^162^ and inhibition of the Wnt/β-catenin pathway.^163^ Nevertheless, the potency of prodigiosin has inspired many researchers to look for analogues. For example, Quesada and co-workers studied the anticancer and anion transport activity of a class of related natural products called tambjamines.^164–167^ Tambjamines contain the same 2,2’-bipyrrole moiety as prodigiosins, but have an imine functionality as protonatable group (for an example, see Scheme 4: compound **14**). Early studies had shown that tambjamines can be potent anionophores^23^ that show cytotoxicity against various cancer cell lines. While detailed mechanistic studies on the anticancer activity of tambjamines have shown different pathways for different analogues and different cell lines (both apoptosis and necrosis have been reported), a common feature is the ability of tambjamines to alter lysosomal and intracellular pH. These changes in pH could be due to the disruption of cellular homeostasis induced by these anionophores. Recently, however, the Quesada group showed that some click-tambjamines (most notably **22**, Scheme 8) are also able to transport lactate anions across liposomal and cellular membranes.^168,169^ Since the lactate concentration is significantly higher in cancer cells,^170^ lactate transport could contribute to the anticancer activity of this class of compounds. Mechanistic studies with **22** on various cancer cell lines (A549 and HeLa) revealed that lactate is transported out of the cells, and this results in acidification of the cytosol and mitochondria, which eventually leads to cell death via apoptosis or necrosis. Furthermore, tambjamine **22** showed synergistic activity with cis-platin, and had a modest selectivity for cancer cells over healthy cells (IC_50_ against healthy MCF10A cells was about 2×–3× higher than against cancer cell lines). These results indicate that lactate could be an interesting target for the development of new anionophores with anticancer activity.

**Scheme 8:**
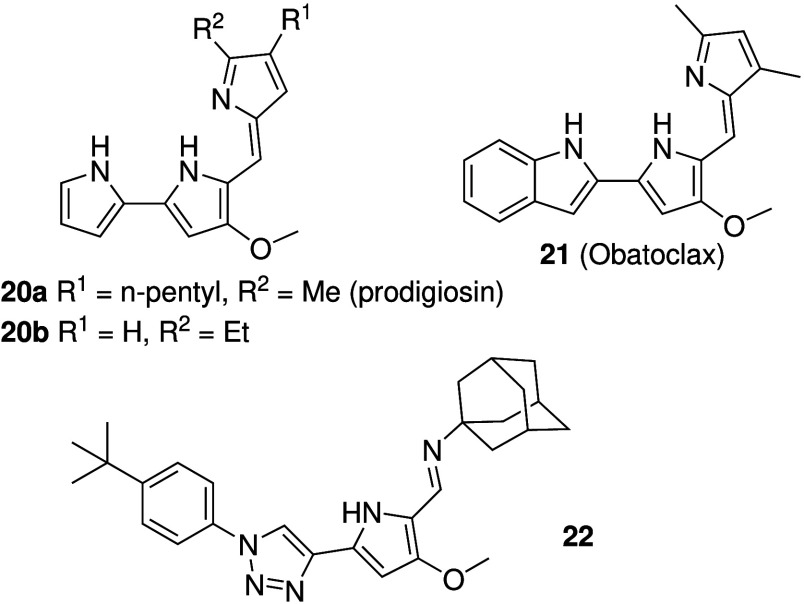
Structure of Prodigiosins **20a**, **20b** and **21**, and Click-Tambjamine **22**

The success of prodigiosins and tambjamines has prompted researchers to develop synthetic transporters that mimic some of the most important structural features of these natural products. Gale and co-workers, for example, developed “perenosins”, which consist of a pyrrole linked through an imine to an indole (e.g., **23**, Scheme 9).^171,172^ Detailed liposome-based assays showed that perenosins function as H^+^/Cl^–^ transporters. *In vitro* studies revealed that the perenosins are selectively cytotoxic against cancer cells (MDA-MB-231) over noncancerous human cells (MCF-10A), and that the cell death mechanism is most likely the induction of apoptosis. Aslam et al. developed a prodigiosin mimic lacking a protonatable functionality (**24**, Scheme 9).^173^ They showed that **24** can transport Cl^–^ across model membranes, and possesses moderate antiproliferative activity against a variety of cancer cell lines. Talukdar and co-workers also reported prodigiosin mimics without protonatable groups (e.g., **25**, Scheme 9).^174^ The NH groups in **25** are somewhat reminiscent to the positions of the NH hydrogen bond donors of protonated prodigiosin. Liposome-based studies showed that **25** can dissipate transmembrane pH gradients and transport a variety of anions across lipid bilayers. The compound was also shown to possess moderate cytotoxicity against MCF-7 cancer cells through an apoptosis mechanism.

**Scheme 9:**
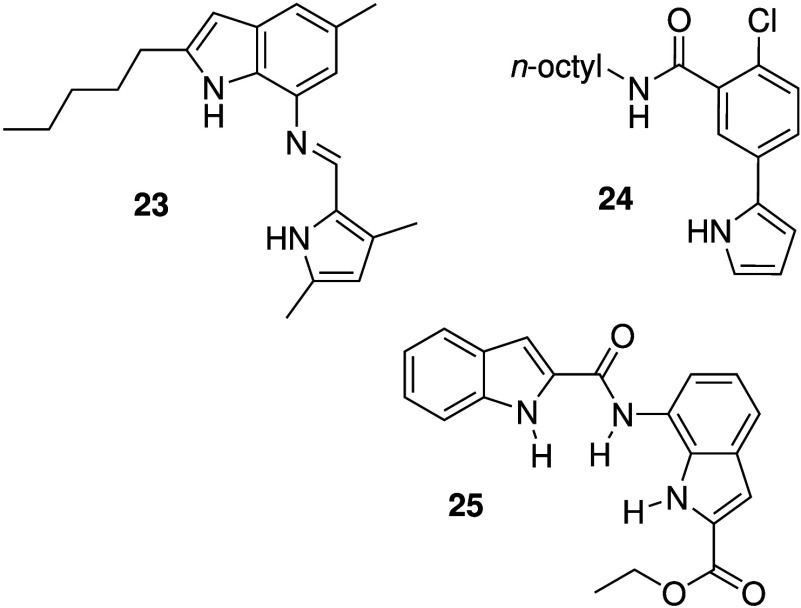
Structure of Synthetic Prodigiosin Mimics **23** (a Perenosin) and **24**–**25**

The anticancer activity of prodigiosins and tambjamines is related to their protonatable nitrogen, which allows H^+^/Cl^–^ transport and subsequent alterations of cellular pH (a known trigger for apoptosis).^175,176^ Not surprisingly, many other synthetic transporters with protonatable nitrogens have been reported to possess anticancer activity. In an early study, Tecilla and de Riccardis showed that a 1,3-*alternate* calixarene derivative appended with protonatable spermidine appendages (**26**, Scheme 10) can facilitate H^+^/Cl^–^ transport in liposomes and display cytotoxicity against J774.A1 cancer cells.^177^ Kocsis and colleagues found that simple triaminopyrimidines (e.g., **27**, Scheme 10), which have a p*K*_a_ value about 7.4, can transport HCl across liposome membranes and induce apoptosis in cancerous and noncancerous cell lines.^178^ Chen and co-workers studied a range of benzimidazole-based anion transporters with various electron-withdrawing groups (e.g., **28**, Scheme 10).^179,180^ These compounds were shown to transport anions in liposomes and cells, deacidify acidic organelles and induce apoptosis. However, little selectivity in cytotoxicity for cancer cells over normal cells was found. Manna and co-workers also reported similar benzimidazole containing anion transporters (e.g., **29**, Scheme 10).^181^ Because the p*K*_a_ of 2-aminobenzimidazoles is higher than benzimidazole (approximately 7.18 and 5.41 respectively),^182^ these compounds are more likely to be protonated at physiological pH. It was found that **29** can facilitate H^+^/Cl^–^ transport as well as electrogenic anion transport in model liposomes, and has antiproliferative activity against cancer cells. More recently, Talukdar and co-workers reported pyridyl-linked benzimidazolyl hydrazones (e.g., **30**, Scheme 10) that show more efficient H^+^/Cl^–^ transport in liposomes than prodigiosin at the same extremely low concentration (50 pM).^183^ Furthermore, the compound was shown to be toxic against cancer cell lines (MCF-7), but not healthy cell lines (HEK293T), and mechanistic studies revealed that alteration of lysosomal pH plays a role in the anticancer activity.

**Scheme 10:**
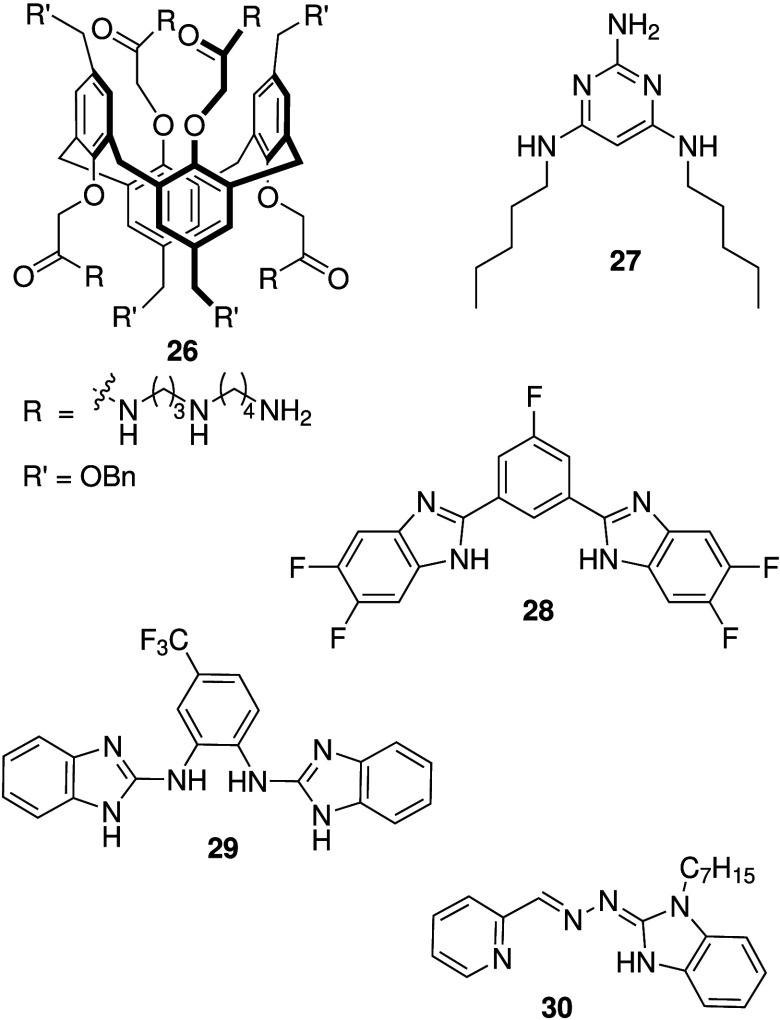
Structure of Protonatable Transporters **26**–**30**

Ureas and thioureas have also been shown to dissipate pH gradients in cells. Although (thio)­ureas do not possess a protonatable group, it has been shown that they are able to transport OH^–^ as neutral transporters, facilitate H^+^/Cl^–^ transport through deprotonation of the acidic NHs of the (thio)­urea functionality, or mediate H^+^ transport via fatty acid flip-flop induced by the anion transporters coupled with protonation/deprotonation of the fatty acid carboxylic acid group.^184–188^ As a result, they have become promising leads for anticancer drugs that function through an anion transport mechanism. For example, Gale and co-workers have shown that tripodal ureas and thioureas (e.g., **13**, Scheme 3),^28^ as well as simple indole-(thio)­ureas (e.g., **31**, Scheme 11)^189^ and a variety of bis-ureas,^190–192^ function as potent anion transporters and induce apoptosis in human cancer cells by increasing the lysosomal pH and/or decreasing the cytosolic pH. The group of Debasis Manna have also reported a number of thiourea-containing transporters that can induce cell death by promoting apoptosis, including diphenylethylenediamine-based compound **32** (Scheme 11).^193^ The same group has also show that quinine-based thioureas such as **33** (which have an additional protonatable nitrogen), and PITENINs such as **34** (which have a deprotonatable phenolic OH in addition to a thiourea moiety), can perform H^+^/Cl^–^ transport in liposomes and display cytotoxicity against cancer cell lines (Scheme 11).^194,195^ Furthermore, **33** was shown to reduce tumor growth in mice, with negligible toxicity to other organs. Pradhan et al. introduced a series of molecular clips based on pyridine-2,6-dicarboxamide appended with two urea groups (e.g., **35**, Scheme 11) and showed that they can assist H^+^/Cl^–^ cotransport in liposomes.^196^ Initial cytotoxicity assays of **35** showed modest activity against cancerous HeLa cells (IC_50_ ≈ 20 μM), but not against noncancerous HEK-293T cells.

**Scheme 11:**
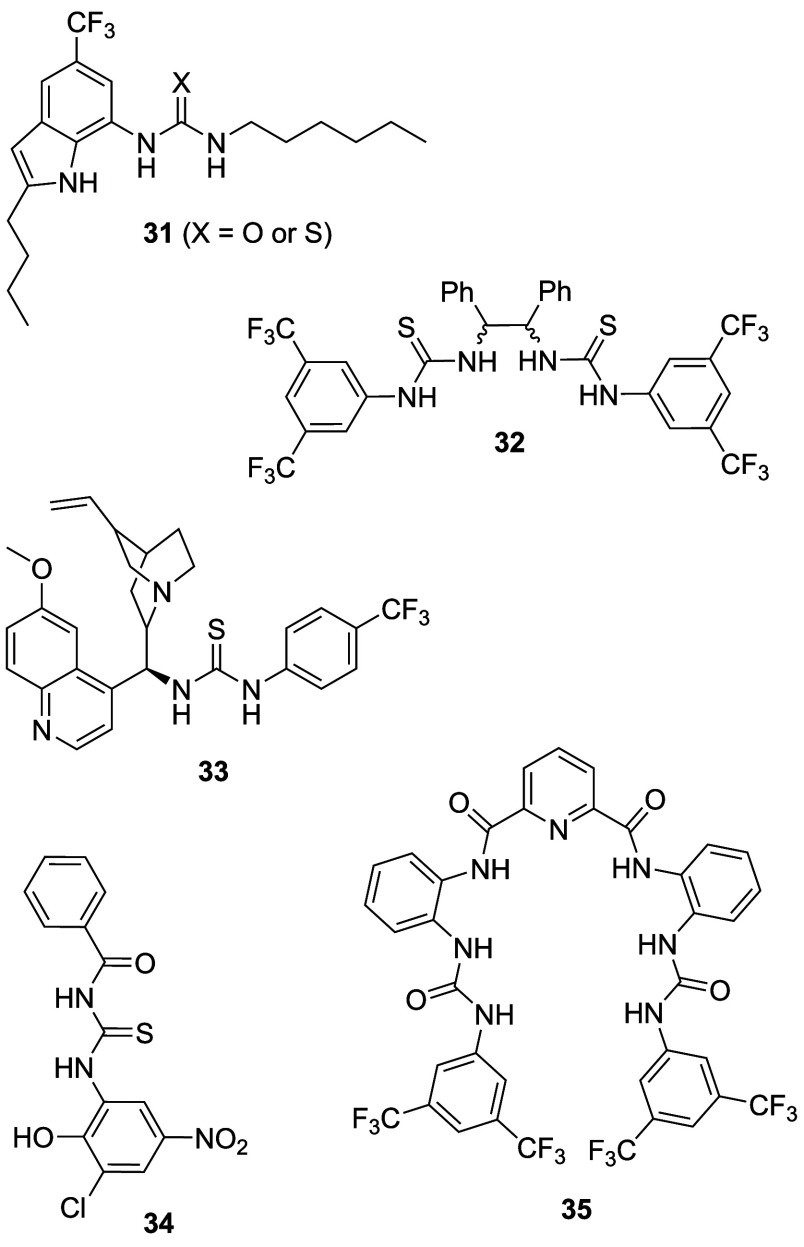
Structure of (Thio)­urea-Based Anionophores **31**–**35**

Squaramides are urea isosteres containing relatively acidic NHs as hydrogen bond donors. Busschaert et al. were the first to report the anion transport activity and detailed anticancer activity of simple squaramides such as **36** (Scheme 12).^26,197^ It was shown that **36** is able to facilitate both electrogenic Cl^–^ transport and electroneutral H^+^/Cl^–^ transport in liposomes, and that it can cause a Cl^–^ influx in FRT cells. It was also found to induce cell death in various cancer cell lines, that cell death was reduced in chloride-free media, and that cell death is probably due to caspase-dependent apoptosis. Furthermore, **36** was shown to increase the lysosomal pH, and subsequently reduce the activity of lysosomal enzymes and disturb autophagy. Chen and co-workers have continued on this work and reported a series of squaramides containing morpholines in order to target the lysosome (e.g., **37**, Scheme 12).^198–200^ The compounds were shown to transport chloride in liposomes and human cells, and were able to increase the pH of the lysosome. However, cytotoxicity data has not been reported yet. The same group also reported that benzimidazole-appended squaramides can function as chloride transporters with cytotoxic activity against cancer cell lines.^201^ More recently, they developed squaramide-hydroxamic acid conjugates that were able to bind to anions via the squaramide unit and iron via the hydroxamic acid unit (**38**, Scheme 12).^202^ It was shown that compound **38** is able to transport Cl^–^ in liposomes, and that it can increase the intracellular Cl^–^ concentration in A549 cells while decreasing the Cl^–^ concentration in the lysosomes of the same cell line (suggesting efflux of chloride out of the lysosomes into the cytosol). Furthermore, **38** was reported to increase lysosomal pH, reduce the intracellular iron concentration, inhibit autophagy, decrease the mitochondrial membrane potential, increase ROS production, and ultimately induce apoptosis. Interestingly, this compound showed potent cytotoxicity against a variety of cancer cell lines with IC_50_ values in the nanomolar range, but showed much weaker cytotoxicity against healthy human liver cells (L02) with IC_50_ values 37x higher than against the cancer cell lines. As a result, **38** also caused a reduction in tumor size in A549 xenografts mouse models, making it one of the few synthetic anionophores with anticancer activity observed *in vivo*.

**Scheme 12:**
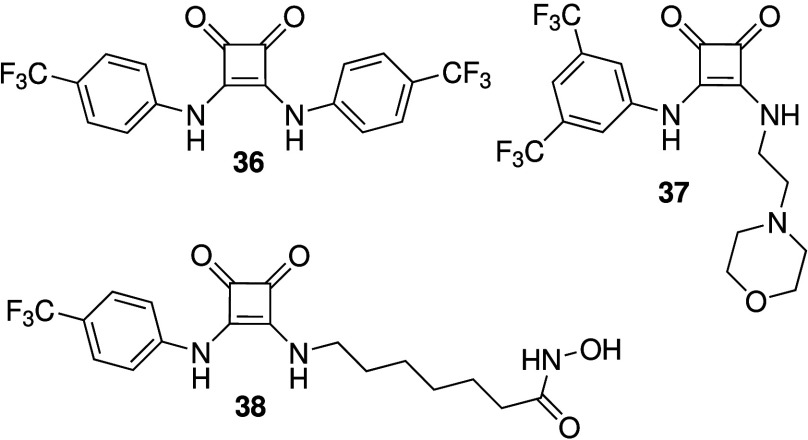
Structure of Squaramide-Based Anionophores **36**–**38**

Other NH hydrogen bond donor motifs have also been investigated. One thorough investigation came from Gale, Sessler, Shin and co-workers.^203^ They reported the activity of diamide-strapped calix[4]­pyrroles (e.g., **39**, Scheme 13). Liposome studies revealed that **39** is able to transport chloride via an anion antiport mechanism, as well as a Na^+^/Cl^–^ symport mechanism. Cell-based studies showed that **39** can alter chloride and sodium concentrations in cells, but does not induce changes in the intracellular pH. Calixpyrrole **39** also leads to cytotoxicity via a caspase-dependent apoptosis pathway, and time-dependent fluorescence studies revealed that the changes in ion concentrations preceded the appearance of apoptotic cells. This provided strong evidence toward the theory that changes in intracellular ion concentrations can lead to apoptosis and hence can be used for the development of anticancer agents. Other NH hydrogen bond donor motifs used as chloride transporters with anticancer activity include the bis-sulfonamides (e.g., **40**), hydroxyphenyl benzamides (e.g., **41**) and benzohydrazides (e.g., **42**)^204^ reported by Talukdar and co-workers,^205,206^ a triazole/aminoxy amide macrocycle described by Chattopadhyay and colleagues (**43**),^207^ and a guanine-based amphiphile (**44**) by Montesarchio and co-workers (Scheme 13).^208^

**Scheme 13:**
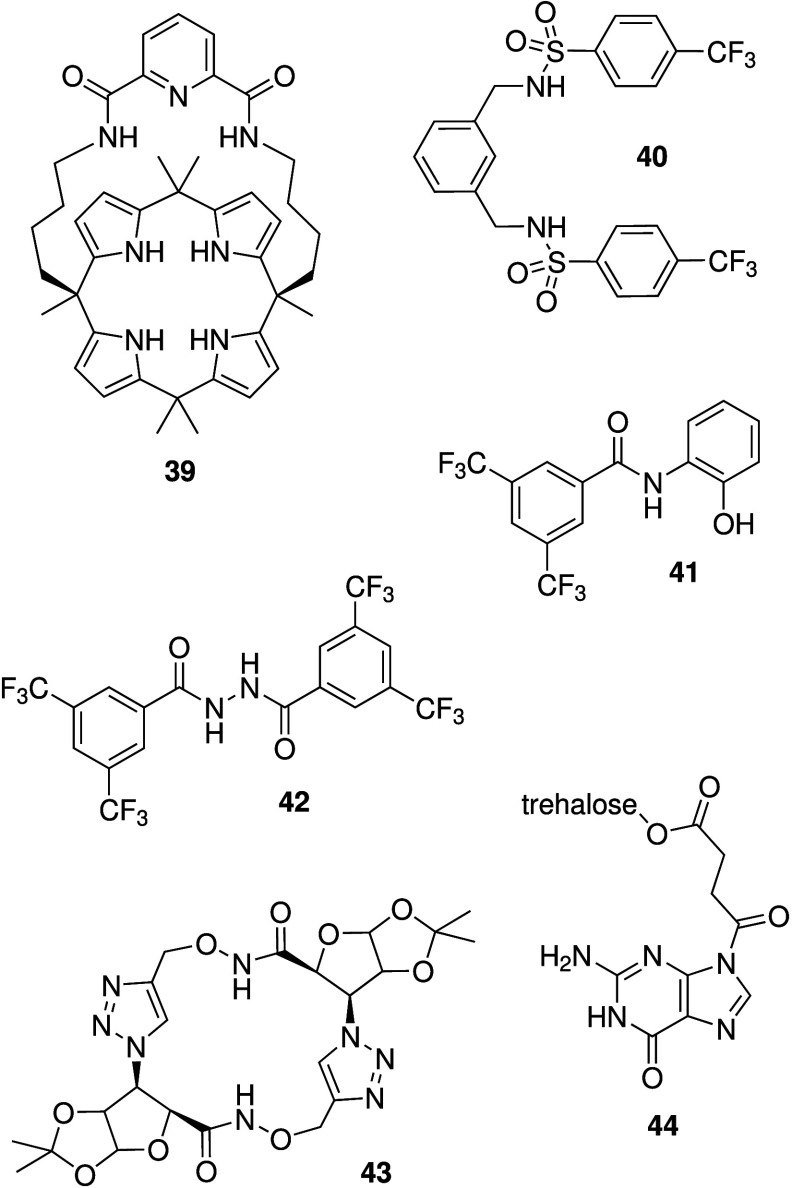
Structure of NH-Based Anionophores **39**–**44**

There are also reports of anionophores with anticancer activity that do not employ NH hydrogen bonding to transport anions across the membrane. Talukdar and co-workers have reported vicinal diol containing small molecules that presumably self-assemble into chloride-selective anion channels (e.g., **45**, Scheme 14).^209^ The compounds were shown to also transport Cl^–^ in human cells and display cytotoxicity against a variety of cancer cells via a caspase-dependent apoptosis pathway. Another small molecule capable of self-assembling into ion channels was reported by Zeng and co-workers (**46**, Scheme 14).^37^ Compound **46** was shown to transport chloride anions in liposomes, presumably through halogen bonding, and displays intermediate cytotoxicity against human breast cancer cells (BT-474). Metal-based anionophores with anticancer activity have been reported by Mao and colleagues (**47** and **48**, Scheme 14). Iridium complex **47** was shown to facilitate anion transport in liposomes, and can lead to cytotoxicity in a variety of cancer cell lines via caspase-independent apoptosis and/or autophagic flux inhibition.^210^ Furthermore, **47** was also proven to inhibit tumor growth in mice models with low systemic toxicity. Manganese porphyrin **48** was shown to transport chloride anions in lipid models and in HeLa cancer cells. Cytotoxicity against HeLa cells was shown to be due to autophagy and immunogenic cell death.^211^ Recently, Gale and co-workers reported the anticancer activity of Pt­(II) complexes with 4 urea-containing ligands.^212^ It was shown that the complexes had excellent chloride transport ability in liposomes and induced apoptosis in cancer cells. Furthermore, the cytotoxicity activity of the Pt­(II)-complexes was found to be dependent on the presence of Cl^–^ in the external medium, suggesting that chloride transport could be relevant for its anticancer activity. However, the authors mention that the chloride binding and transport ability of the complexes is due to hydrogen bonding interactions with the urea-containing ligands, and that the metal center merely serves to preorganize the binding pockets and is not involved in anion binding.

**Scheme 14:**
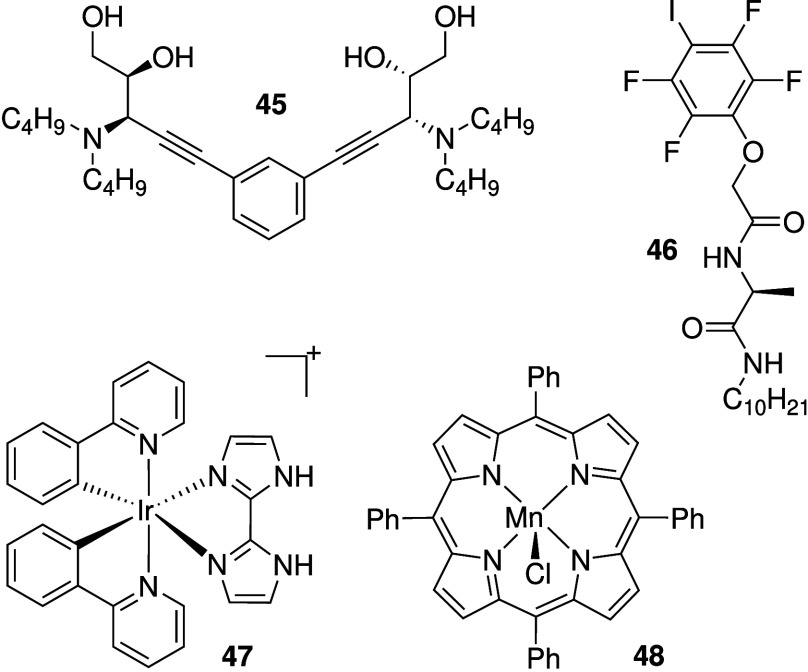
Structure of Anionophores **45**–**48** That Do Not Possess NH Hydrogen Bond Donors

Most mechanistic studies have shown that the anticancer activity of artificial anionophores is the result of deacidification of lysosomes due to ion transport, leading to apoptosis and/or inhibition of autophagy. It is therefore conceivable that appending anionophores with a lysosome targeting moiety could increase their potency. For this reason, Soto-Cerrato and colleagues prepared a click-tambjamine derivative containing a morpholine group for lysosome targeting (**49**, Scheme 15).^213^ Co-localization experiments using confocal microscopy confirmed that **49** is predominantly located in the lysosomes. It was shown that **49** can facilitate anion transport and dissipate pH gradients across liposomal membranes, while cytotoxicity studies confirmed modest anticancer activity against 3 different lung cancer cell lines (A549, SW900 and DMS53). Mechanistic studies revealed that treatment with **49** causes deacidification of the lysosomes and a decrease in the pH of the cytosol, which leads to blocking of late-phase autophagy and induction of apoptosis or necrosis depending on the cell line. Gale and co-workers took this concept one step further and designed a family of urea-based anionophores tethered with groups to target either the endoplasmic reticulum (ER), lysosomes or mitochondria (**50**–**53**, Scheme 15).^214^ The compounds were shown to facilitate H^+^/Cl^–^ transport in POPC liposomes, albeit to a different extent (**50** > **51** > **53** > **52**). Fluorescence imaging confirmed that each anionophore was predominantly located in their intended organelle. Interestingly, cell viability assays revealed that the lysosome and ER targeting compounds (**50** and **51**) showed higher cytotoxicity against A549 human lung carcinoma compared to the nontargeted anionophore **53** and mitochondria targeting compound **52**, but only the lysosome targeting anionophore **50** showed better selectivity for cancer cells over healthy cells. While Gale and co-workers did not see an improvement with mitochondria targeting, Ren and co-workers recently reported mitochondria-targeting self-assembled ion channels based on cholic acid (**54**, Scheme 15).^215^ The authors reasoned that synthetic ion channels targeting mitochondria could increase the selectivity for cancer cells because the mitochondrial membrane potential in cancer cells is ∼60 mV higher than in healthy cells. Liposome and planar lipid bilayer studies suggest that **54** can self-assemble into anion-selective pores at low concentration and nanopores capable of transporting larger molecules such as carboxyfluorescein at higher concentrations. Cell-based studies showed that **54** is specifically localized in the mitochondria and shows cytotoxicity against cancer cells selectively over healthy cells (IC_50_ = 3.8 μM against cancerous HepG2 cells and IC_50_ = 16.6 μM against healthy HHl-5 cells). Mechanistic studies revealed that treatment with **54** leads to mitochondrial membrane depolarization, increased ROS production, release of cytochrome c, and ultimately apoptosis. While further studies are needed, these 3 examples indicated that there is a potential in using organelle-targeting strategies to increase the potency (and potentially selectivity) of synthetic anion transporters toward anticancer activity.

**Scheme 15:**
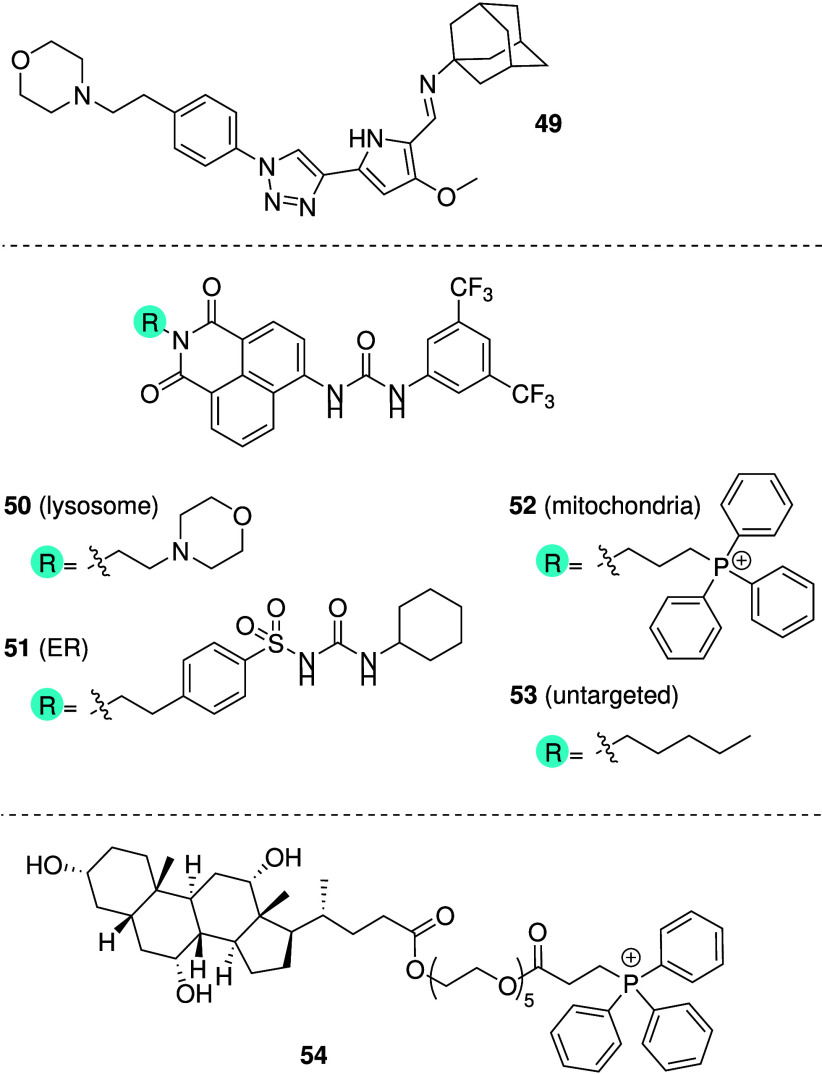
Structure of Organelle-Targeting Anionophores **49**–**54**

As shown by the many examples discussed above, there is reason to believe that anion transport can induce anticancer activity. However, only a few anionophores have been shown to exhibit selective toxicity against cancer cells over healthy human cells,^169,171,172,181,189,201,202,206^ or have been shown to reduce tumor growth in animal models.^167,195,202,210^ Most studies either did not investigate selectivity, or reported little selectivity for cancer cell lines (refs 165, 174, 178, 179, 191, 193, 194, 205,^208^,^209^, and^212^ ). This is not completely surprising as maintaining cellular homeostasis is essential for survival, and disruption of this process is likely to be toxic for all cells. Nonetheless, some of the above-mentioned examples (as well as the examples discussed under “cystic fibrosis”) indicate that selectivity is possible. This could be due to the altered transport protein regulation in cancer cells, and the reversed pH gradient in cancer cells compared to healthy cells (and maybe by organelle targeting).^216^ It is therefore essential for the field to determine the characteristics needed for an anion transporter to be selective for cancer cells over healthy cells. Alternatively, the selectivity problem could be overcome by using transporters that can be “switched on” in cancer cells through endogenous or external factors, or by using targeted delivery systems. A variety of switchable anionophores have therefore appeared in literature (see section 3.2),^18,217–228^ but only a few have been shown to function in biological systems. In healthy cells, the intracellular pH is lower than the extracellular pH (pH_in_ = 7.2, pH_out_ = 7.4), while this is reversed in cancer cells (pH_in_ > 7.4, pH_out_ = 6.2–6.9).^229–231^ Therefore many researchers have been trying to develop anion transporters that are selectively switched on in the acidic environment surrounding cancer cells. One of the few examples where this effect was shown *in vitro*, came from Alfonso and co-workers.^232,233^ They developed fluorine-substituted pseudopeptidic cages that can bind and transport chloride anions when protonated (**55**, Scheme 16). It was shown that **55** displays cytotoxicity against cancer cells only in acidic extracellular environments that mimic solid tumors, rendering this compound essentially nontoxic at pH 7.5 (healthy cells) to highly toxic at pH 6.2 (solid tumors) with an IC_50_ value of 25 μM against A549 cells at this pH.

**Scheme 16:**
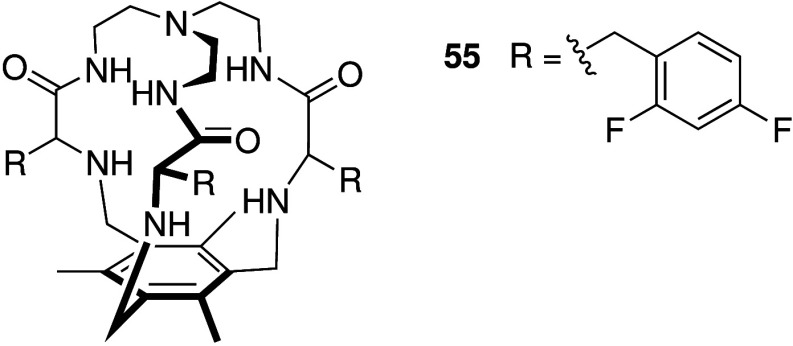
Structure of pH-Switchable Anionophore **55**

Other endogenous factors that can be used for switching are GSH (also called glutathione, a biological antioxidant that is well-known to be overexpressed in cancer cells),^234–236^ and enzymes that are upregulated in cancer (e.g., esterase).^237,238^ Talukdar and co-workers have used both methods to develop anion transporters than can be selectively activated in cancer cells. They had previously shown that isophthalamide derivative **56** (Scheme 17a) can self-assemble into an ion channel that can facilitate Na^+^/Cl^–^ symport in model membranes, and induces cytotoxicity against breast cancer cell line MCF7 through an apoptosis pathway. The group then showed that appending the phenol group with methyl pivalate generates an inactive compound that is converted to the active compound in the presence of esterase (Scheme 17a).^239^ In addition, they also appended the OH group with a 2,4-dinitrobenzenesulfonyl group that can be cleaved by GSH to generate the active transporter (Scheme 17a).^240,241^ Both activatable transporters were found to induce apoptosis in MCF-7 cancer cells, but the activity against healthy cells was not reported. Recently, the same group also attempted to leverage the abundance of NAD­(P)­H:quinone acceptor oxidoreductase 1 (NQO1) in cancer cells to develop enzyme-activatable synthetic transporters (Scheme 17b).^242^ Anionophore **57** was shown to function as a H^+^/Cl^–^ transporter in EYPC liposomes, and this activity could be switched off by converting it into NQO1-cleavable pro-transporters. Both anionophore **57** and the protransporters were shown to display cytotoxicity against MCF-7 cancer cells (IC_50_ ≈ 8 μM), but not against healthy MEF cells, suggesting that the prodrug approach was not necessary for this compound as it already showed selectivity. Gale and colleagues used a different approach to harness the high GSH concentration in cancer cells.^222^ They reported benzimidazole-pyridine compounds, which resemble some of Chen’s benzimidazoles (e.g., **28**, Scheme 10), and showed that their anion transport activity can be switched off by forming Au^III^ complexes (Scheme 17c). Given the high affinity of gold for thiols, such as GSH, it was reasoned that high GSH concentrations can remove the gold ion and release the active anion transporter. Liposome-based studies using POPC indicated that anion transport activity can indeed be restored upon *in situ* incubation of **58-Au** ^**III**^ complex with GSH (or other reducing agents). Cytotoxicity studies also showed that the **58-Au** ^**III**^ complex was less toxic to healthy human cells (HEK293 and MCF10A) compared to the free transporter **58**, but the toxicity against cancer cells (SW620) remained high, suggesting that the higher GSH concentration in cancer cells can release the active transporter but the lower GSH content in healthy cells cannot.

**Scheme 17:**
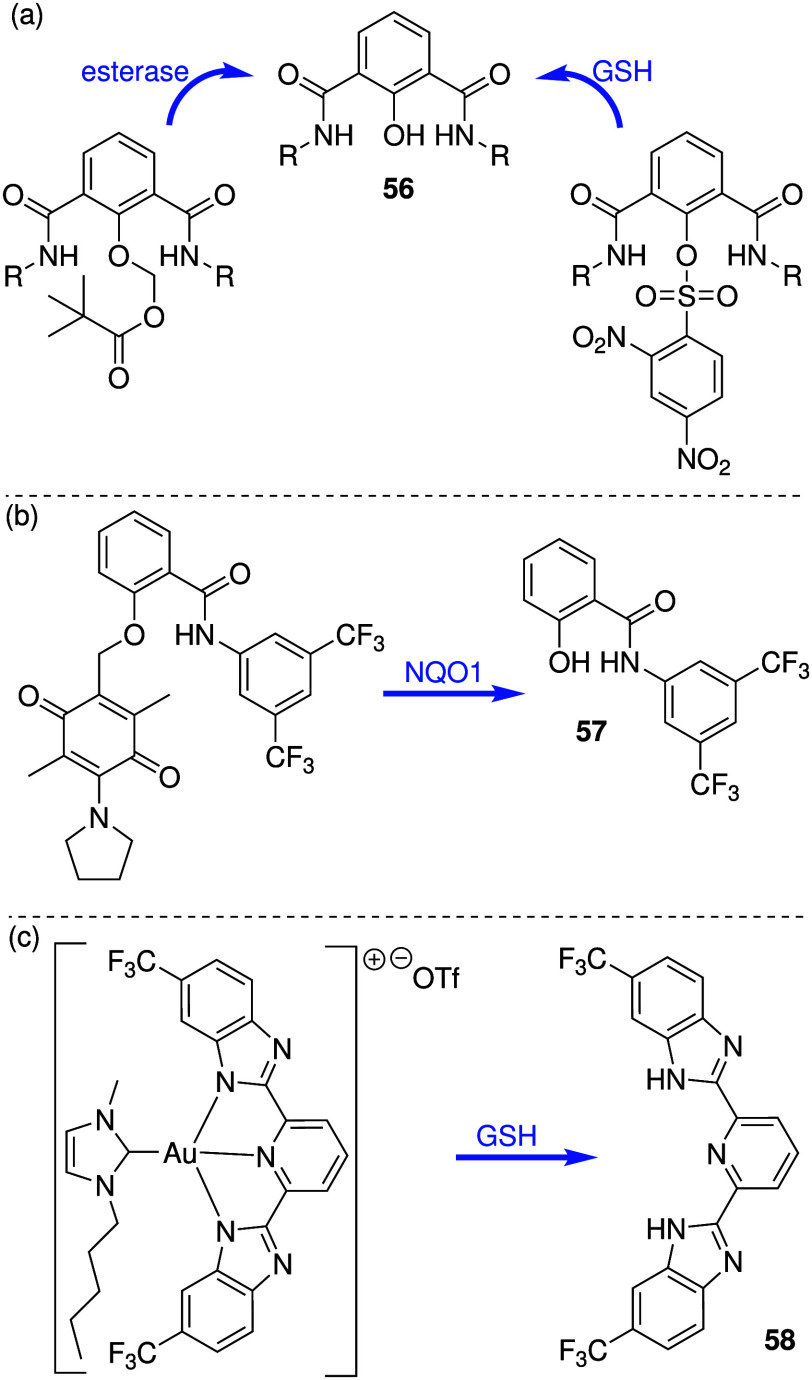
(a) Structure of Activatable Anion Channel **56** and Its Precursors; (b) Structure of Anionophore **57** and Its NQO1 Cleavable Precursor; (c) Structure of Anionophore **58** and its Au^III^ Complex Precursor

The use of external factors, such as light, to switch on transmembrane anion transporters can be advantageous if the difference in endogenous triggers between cancerous and healthy cells is small. The Talukdar group reported photoactivatable anion transporters with biological activity (e.g., **59**, Scheme 18a).^243^ It was shown that the anion transport activity of **59** in liposomes can be switched on upon irradiation with 365 nm light. Furthermore, **59** only showed cytotoxicity against MCF-7 cells after irradiation, suggesting that it is an excellent switchable transporter in cells. Recently, they employed the same *o*-nitrobenzyl protection strategy to generate a benzimidazole-based photoactivatable anionophore with anticancer activity upon irradiation.^244^ The group of Debasis Manna also used the *o*-nitrobenzyl group to prepare photoswitchable anionophore **60** based on a β-carboline-thiourea scaffold (which already has pH-dependent anion transport behavior), and showed that it had excellent anticancer activity and selectivity (Scheme 18b).^245^ Similarly, Ren and co-workers used the *o*-nitrobenzyl group to photocage a classic isophthalamide-based chloride channel and showed that it had good anticancer activity and selectivity.^246^

**Scheme 18:**
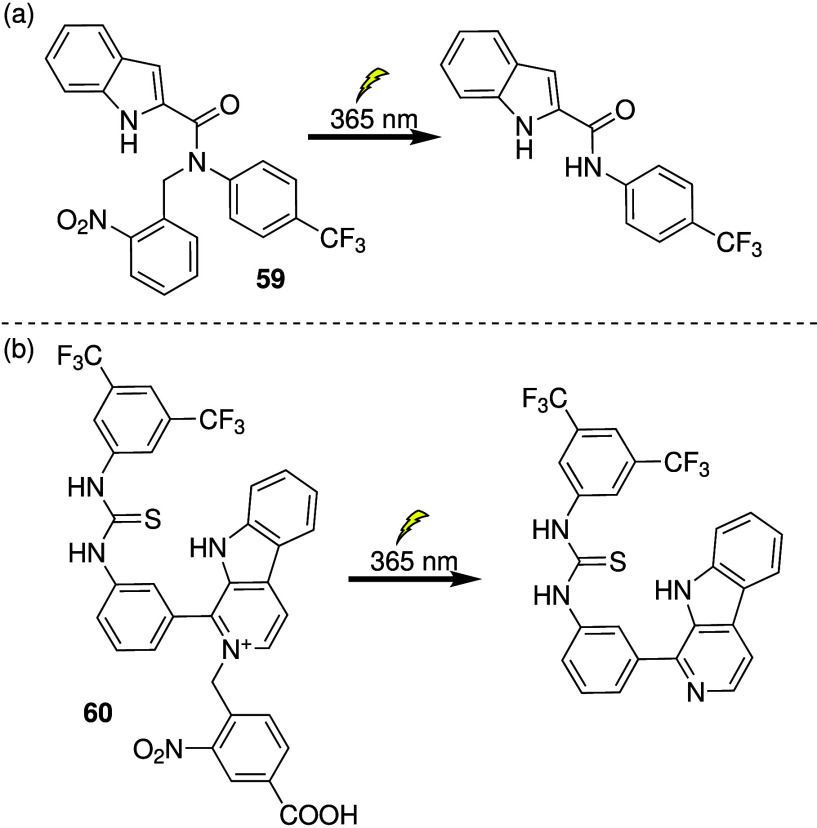
Structure of Photocaged Anionophores and the Active Transporter upon Photocleavage: (a) **59** and (b) **60**

In addition to the selectivity problem, the commercial use of synthetic anion transporters as anticancer drugs is also hindered by their limited deliverability. The problem of deliverability of the highly hydrophobic anion transporters was already discussed for channelopathies, but is even more acute for anticancer drugs which are administered systemically. For the field to progress further in the direction of anticancer activity, both the selectivity and deliverability problem will need to be addressed. This could be achieved using targeted delivery systems, whereby a toxic anticancer drug is selectively delivered to cancer cells in order to increase its therapeutic effect and reduce its side effects.^247^ A few advances toward this goal have been reported recently. The group of Ricardo Pérez-Tomás, in collaboration with Roberto Quesada, have shown that tambjamine aniniophores can be delivered using polyurea/polyurethane nanocapsules designed to be taken up preferentially at the lower pH near cancer cells and to be degraded by GSH inside cancer cells.^248^ The nanocapsules were tested both *in vitro* and *in vivo* and were shown to reduce tumor growth in mice and to reduce the systemic toxicity compared to unencapsulated tambjamine. Another advance came from the group of Debasis Manna.^249^ They designed a bis-thiourea anionophore that was appended with a cleavable peptide that is a known substrate for integrin (**61**, Scheme 19). The peptide can improve the water solubility of the artificial transporter, but it also allows selective uptake in cancer cells due to integrin receptor-mediated endocytosis because some integrins are overexpressed in cancer cells. Furthermore, inside the cancer cells the active transporter can be released by cleavage of the disulfide bond by GSH (which is also present at higher concentrations in cancer cells than in normal cells). It was shown that the active anionophore can indeed be generated by incubation with GSH and function as a moderate H^+^/Cl^–^ transporter in 8:2 EYPC:cholesterol liposomes. The active transport itself was shown to be cytotoxic to both cancerous and healthy human cells, whereas the proanionophore **61** showed selective toxicity against cancer cells (A375) over healthy cells (HEK293T), especially under serum starvation conditions. Finally, Chen and co-workers also developed a smart delivery method for targeted anionophore delivery.^250^ They reported the anion transport activity of a different bis-thiourea (**62**, Scheme 19), and showed that it is cytotoxic to osteosarcoma cancer cells. Detailed *in vitro* mechanistic studies further revealed that **62** can increase intracellular chloride concentrations, and can induce cell death through multiple pathways (activation of endoplasmic reticulum stress, autophagy, apoptosis and cell cycle arrest). To show the *in vivo* anticancer potential, the authors devised a delivery system consisting of liposomes containing anionophore **62** as well as an osteosarcoma-targeting peptide (TPPRVPLLTFGS tethered to tetradecanoic acid). It was shown that this delivery system was able to inhibit tumor growth in subcutaneous xenograft tumor and lung metastasis models of human osteosarcoma in mice and induced negligible toxicity. These last two examples show that careful design of anionophores to overcome both selectivity and delivery problems is possible, although further investigations are still necessary.

**Scheme 19:**
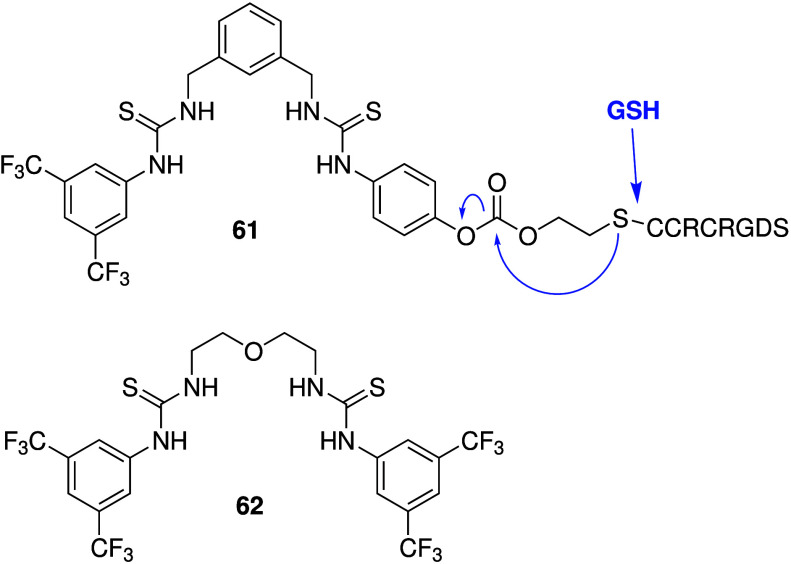
Structure of RGD-Peptide Linked Proanionophore **61**, and the Mechanism of GSH-Mediated Cleavage (Amino Acids of the Peptide Are Given as 1 Letter Codes), As Well As the Structure of Anionophore **62**

### 2.4 Infectious Diseases

Another area where the toxicity of artificial anion transporters could be beneficial is for the treatment of infectious diseases (bacterial, fungal, viral, and parasitic infections). There is currently a pressing need for the development of novel antimicrobial drugs due to a combination of resistance against existing drugs and a lack of investment in new drugs. Membrane-active compounds, such as ionophores, have great potential in this regard because they are less prone to resistance mechanisms.^251,252^ Cationophores such as valinomycin, monensin and salinomycin are a well-studies class of antibiotics and are commercially used a veterinary antibiotics. While the antibacterial, antiviral and antimalarial activity of the natural product prodigiosin is well-known,^253–257^ synthetic anionophores have only recently started to be investigated for their potential antimicrobial activity.

#### 2.4.1 Antibacterial Agents

The antimicrobial compounds that have attracted the most attention are antibiotics. Cationophores (both natural products such as valinomycin and synthetic cationophores) are well-known for their antibacterial activity, even though they are only used for veterinary/agricultural use and not in humans.^258–260^ Similarly, antimicrobial peptides (AMPs) are a large class of membrane-active peptides that have been intensely studied for their antibacterial activity. Most AMPs are polycationic and amphiphilic in nature, which allows them to bind to the negatively charged membranes of bacteria.^261,262^ The exact mechanism of AMPs is still up for debate, but it is generally believed that membrane binding eventually leads to severe membrane disruption (lysis) or the formation of nonselective pores. The success of these two categories shows that membrane-targeting is a valid strategy for the development of novel antibiotics. Selectivity for bacterial membranes over human membranes is possible due to the different lipid composition,^263^ increased curvature of bacterial membranes, and the larger membrane potential of bacteria.^264^ In addition, due to the small size of bacteria, only around 100 monovalent ions need to be transported to change the membrane potential in bacteria by 1 mV, which is 1000x fewer ions that need to be transported to achieve the same change in membrane potential in mammalian cells.^264^ Despite these advantages, small molecule anion transporters have only recently started to be investigated for their antibacterial activity.

Most early work on the use of anionophores as antibacterial agents has been conducted by Schmitzer and co-workers. They have reported a number of simple cationic (benz)­imidazolium-based compounds that function as chloride transporters and possess antibacterial activity (e.g., **63**–**67**, Scheme 20).^265^ For example, the benzimidazolium salts **63**–**65** were shown to function as chloride transporters in liposomes, and showed antibacterial activity against the Gram-positive bacterium *B. thuringiensis* with minimum inhibitory concentrations (MIC) around 10 μM.^266,267^ Furthermore, these compounds showed little hemolytic activity and mechanistic studies revealed that the antibacterial activity is likely due to membrane depolarization. Later studies on analogues of **63** also found that these benzimidazolium salts have activity against methicillin-resistant *S. aureus*, vancomycin-resistant *E. faecium* and even the Gram-negative bacterium *E. coli*.^268^ These compounds were also shown to possess antibiofilm activity.^268–270^ Similarly, imidazolium-appended binaphthols (e.g., **66**) were shown to facilitate both Cl^–^/NO_3_^–^ antiport and H^+^/Cl^–^ symport in liposomes, and displayed high antibacterial activity against the Gram-positive bacteria *B. thuringiensis* and *Listeria seeligeri*.^271,272^ Interestingly, the group also reported adamantyl-appended imidazolium salts (e.g., **67**) and showed that the transport and antibacterial activity can be switched off by forming an inclusion complex with β-cyclodextrin.^273,274^ Through the addition of a competitive guest (adamantane-ethanol), **67** can be released and the activity is switched on again. This approach could be useful to reduce the toxicity to human cells of anion transporters by only releasing the active compound where needed. In more recent work, the Schmitzer group incorporated benzimidazoles into metal–organic frameworks and showed that this can also facilitate the transport of anions and antibiotics.^275^

**Scheme 20:**
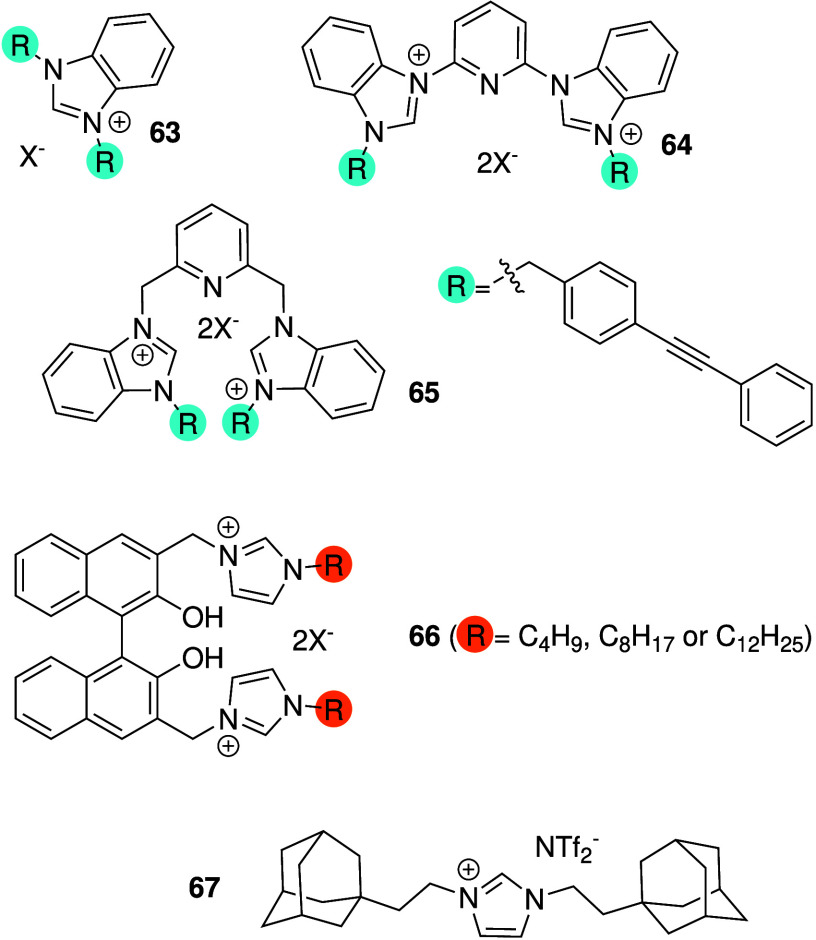
Benzimidazole Anionophores **63**–**67** with Antibacterial Activity Reported by Schmitzer and Co-workers

Other groups have also looked at the antibacterial activity of benzimidazole-containing compounds. Davis and Amendola reported a benzimidazolium-based organic cage (**68**, Scheme 21), and showed that it displayed weak chloride transport ability in liposomes.^276^ Nonetheless, **68** displayed antibacterial activity against *S. aureus* (MIC = 13.29 μM) and negligible cytotoxicity against human cell lines. In addition to benzimidazole, there are many other cationic anionophores with reported antibacterial activity. This is probably due to the fact that bacterial membranes contain large amounts of negatively charged phospholipids (e.g., phosphatidylglycerol (PG) and cardiolipin (CDL)), while human cells only display small amounts of negatively charged lipids.^263^ This implies that cationic species often have a preference for bacterial membranes. Schmitzer and co-workers developed the cationic sterol-based molecular umbrella **69** (Scheme 21) and showed that it forms anion-selective channels in liposomes and possesses antibacterial activity against Gram-positive bacteria (MIC = 8–10 μM against *B. thuringiensis*).^277^ Similarly, Regen and co-workers showed that cationic derivatives of the sterol squalamine can also form chloride channels^278^ and many of them display broad-range antibacterial activity.^279,280^

**Scheme 21:**
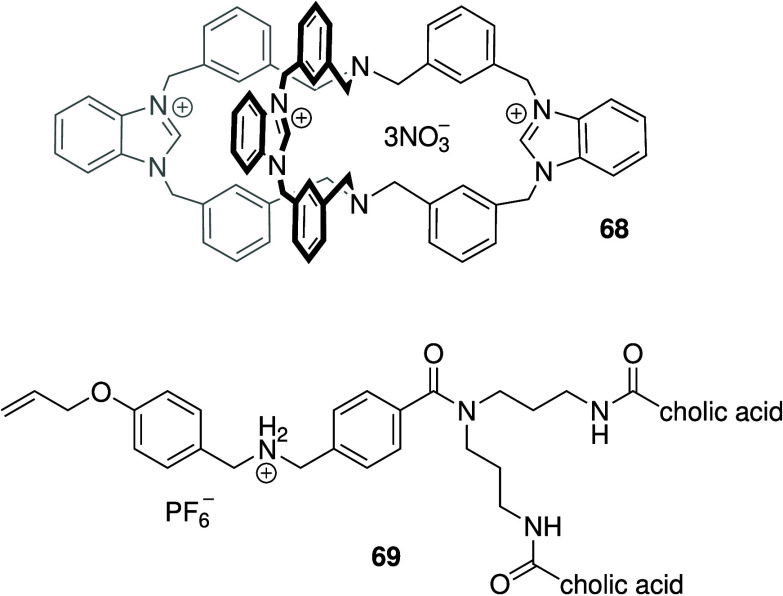
Cationic Anionophores **68** and **69** with Antibacterial Activity

In addition to purely cationic salts, compounds with protonatable nitrogen atoms have also been employed to prepare anionophores with antibacterial activity. Such compounds can potentially function as H^+^/Cl^–^ transporters, and the cationic charge resulting from protonation could contribute to the selectivity toward the anionic membranes of bacteria. Talukdar and co-workers reported a rigid bis-melamine compound (**70**, Scheme 22), which was shown to bind HCl in the solid state.^226,281^ Liposome-based studies indicated that **70** can function as a H^+^/Cl^–^ transporter, and that anion transport is dependent on pH (with best activity observed at pH = 7.0). Furthermore, **70** showed bactericidal activity against a range of Gram-positive bacteria with MIC values from 0.5–2 μg/mL, and also displayed antibiofilm activity in clinically relevant MRSA bacteria (methicillin-resistant *S. aureus*). Mechanistic studies in bacteria suggest that **70** induces membrane depolarization and increases membrane permeability, confirming a membrane-related mode of action. The same group also reported on the putative antibacterial activity of their highly potent pyridyl-hydrazone H^+^/Cl^–^ transporters that had previously been shown to possess anticancer activity (e.g., **30**, Scheme 10).^282^ Growth curves obtained for a variety of Gram-negative bacteria in the presence of **30** and analogues show a reduction in optical density, but full inhibition of bacterial growth was not observed for any of the compounds. Traditional MIC values were therefore not given. Manna and co-workers reported the antibacterial activity of 4-aminoquinazoline-thiourea derivatives (e.g., **71**, Scheme 22).^283^ It was shown that **71** functions as a H^+^/Cl^–^ transporter in liposomes, and that transport activity is greater at lower pH (presumably due to protonation of the 4-aminoquinazoline moiety). It was also shown that **71** can exhibit antibacterial activity against Gram-positive bacteria, with an MIC value of 2.5 μM against MRSA. Furthermore, staining *S. aureus* with the chloride-sensitive dye MQAE revealed that **71** can increase the intracellular chloride concentration in this bacterium and thus provides evidence for the theory that the anionophore activity of **71** is responsible for the observed antibacterial activity.

**Scheme 22:**
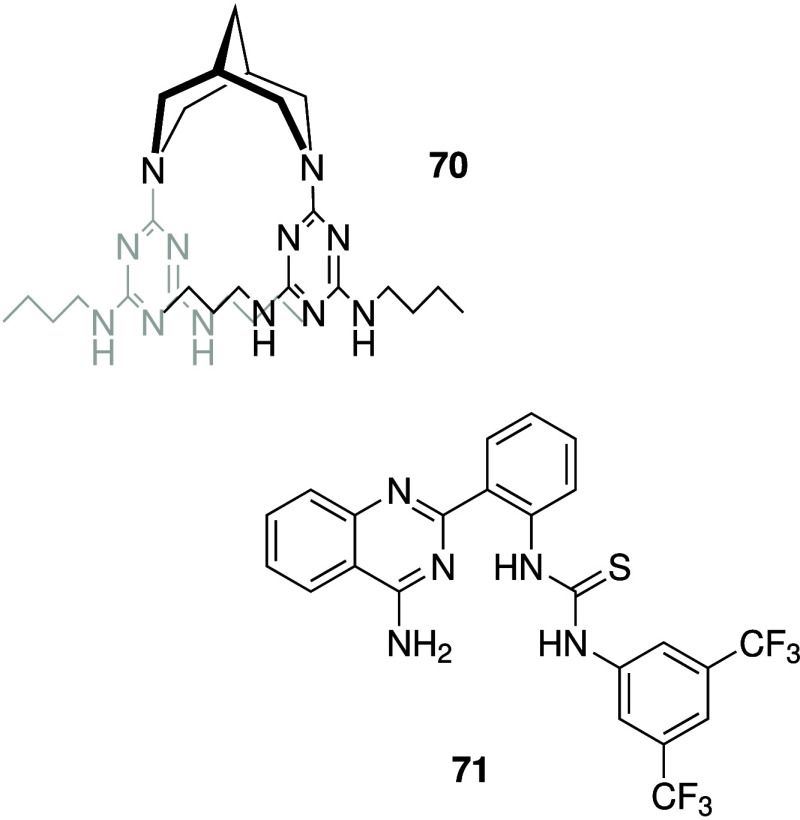
Antibacterial Anionophores with Protonatable Nitrogen Atoms **70** and **71**

Natural products remain the inspiration for most antibiotics, and anion transport-based antibiotics are no exception. Webb and co-workers developed α-aminoisobutyric acid foldamers related to the natural product group of peptaibols whose transport activity can be switched on upon the addition of copper­(II)­chloride (e.g., **72**, Scheme 23).^284^ Liposome and planar lipid bilayer experiments revealed that these foldamers function as ion channels capable of transporting both cations and anions, and do not cause membrane lysis. In addition, the foldamer shows antibacterial activity against *B. megaterium* and displays minimal hemolytic activity against human red blood cells. Quesada and co-workers have long been inspired by the biological activity of prodigiosins and tambjamanes, and thus also investigated the antibacterial activity of a number of indole-decorated tambjamine-like compounds (e.g., **73**, Scheme 23).^285^ Liposome-based studies confirmed that these tambjamines function as anionophores, and bacterial studies revealed that **73** had potent antibacterial activity against a variety of Gram-positive and Gram-negative bacteria (MIC ≤ 12.5 μM). Furthermore, **73** was active against drug-resistant clinical isolates of these bacterial species and showed minimal hemolytic activity, making it a promising new antibiotic.

**Scheme 23:**
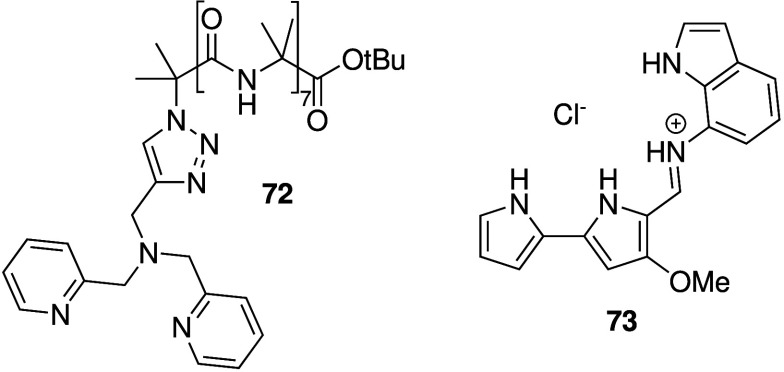
Natural Product Analogues **72** and **73** with Antibacterial Activity

As tambjamines (and prodigiosins) appear to display both anticancer and antibacterial activity, it comes as no surprise that many of the structural motives that have been found in anionophores with anticancer activity (e.g., ureas, thioureas, squaramides, ...) have also been tested for antibacterial activity. Gale, Roelens and Sessler reported a series of aminopyrrolic compounds (e.g., **74**, Scheme 24) with antibacterial activity against methicillin-resistant *S. aureus*.^286^ Liposome-based studies suggested that these compounds can facilitate cation/anion cotransport and transport activity was found to correlate roughly with antibacterial activity. Furthermore, it was also shown that the known antibiotics thiocarlide and trichlorocarbanalide (Scheme 24) can function as chloride transporters in liposomes, and *vice versa* it was found that the known *tren*-based thiourea transporters (analogues of compound **13**, Scheme 3) display potent antibacterial activity. These findings show that even simple anionophores have potential as antibacterial agents. This was later further corroborated by the Busschaert group, who studied the antibacterial mechanism of a simple monourea (**75**, Scheme 24), which showed MIC values against various Gram-positive bacteria <6.5 μM.^287^ Bacterial cytological profiling was used to elucidate the mode of action of **75**. It was found that it functions through a membrane-related mechanism, but with a mechanism distinct from the cationophore calcimycin or the pore-former nisin. Liposome-based studies then revealed that the most likely mode of action of this simple urea is transmembrane anion transport. Another (thio)­urea with antibacterial properties was recently reported by Garcia-López.^288^ They reported a rotaxane with a thiourea-containing ring (**76**, Scheme 24) that was shown to shuttle chloride anions across EYPC liposomes. Low concentrations of this rotaxane (2 μM) were able to fully inhibit the growth of *S. aureus* bacteria, but only in the presence of 1 M NaCl or 20 μM arachidonic acid to increase membrane fluidity, suggesting a transmembrane transport related mechanism.

**Scheme 24:**
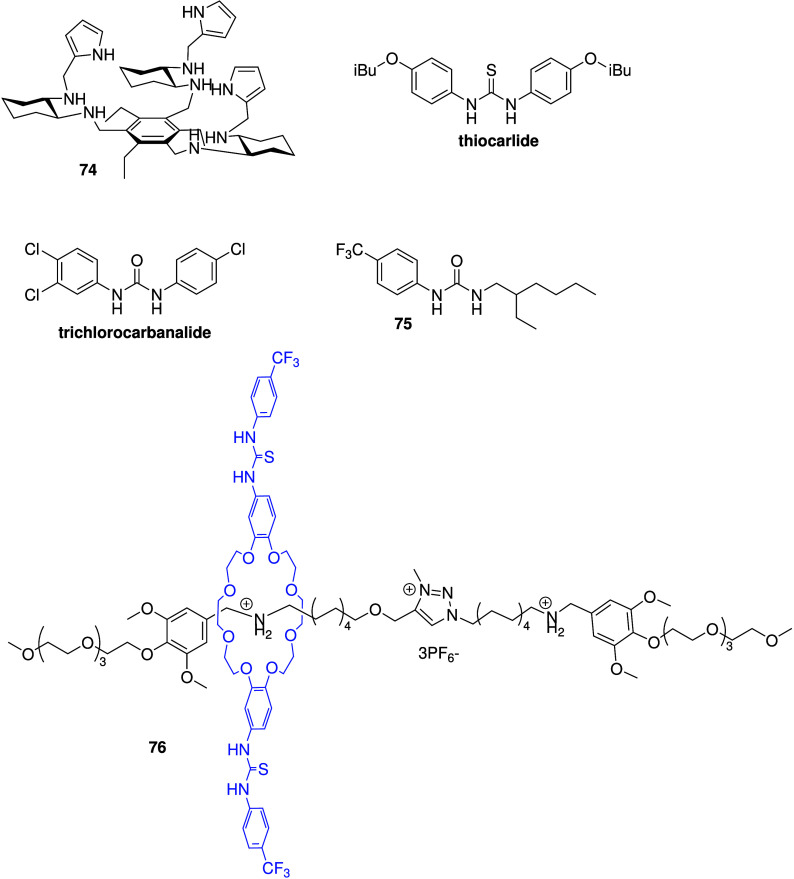
Various Anionophores with Antibacterial Activity

Other classic anionophore motives have also been investigated for antibacterial activity. Elmes and colleagues have reported squaramide-type compounds with putative antibacterial activity, called “squindoles” (e.g., **77**, Scheme 25).^289^ Liposome studies suggested that **77** can facilitate both Cl^–^ uniport and H^+^/Cl^–^ symport. While a reduction of bacterial growth was reported against MRSA, complete growth inhibition was not achieved at any of the tested concentrations and traditional MIC values are therefore not reported (instead IC_50_ values were used, which are less common for bacteria). Nevertheless, detailed mechanistic studies using fluorescence microscopy and proteomic analysis did suggest that **77** can increase the intracellular Cl^–^ concentration and activate a variety of stress response pathways in the bacteria. Chmielewski and co-workers also reported on the antibacterial activity of their (thio)­amidocarbazoles (e.g., **78**, Scheme 25). Previous studies had already shown that these compounds function as potent anion transporters for chloride,^290–292^ organic anions,^133^ and amino acids,^293^ and are also able to facilitate H^+^/Cl^–^ transport and dissipate pH gradients in liposomes. Compound **78** was found to have potent antibacterial activity against Gram-positive bacteria (*B. subtilis* and *S. aureus*), with MIC values <1 μM, but not against Gram-negative bacteria.^294^

**Scheme 25:**
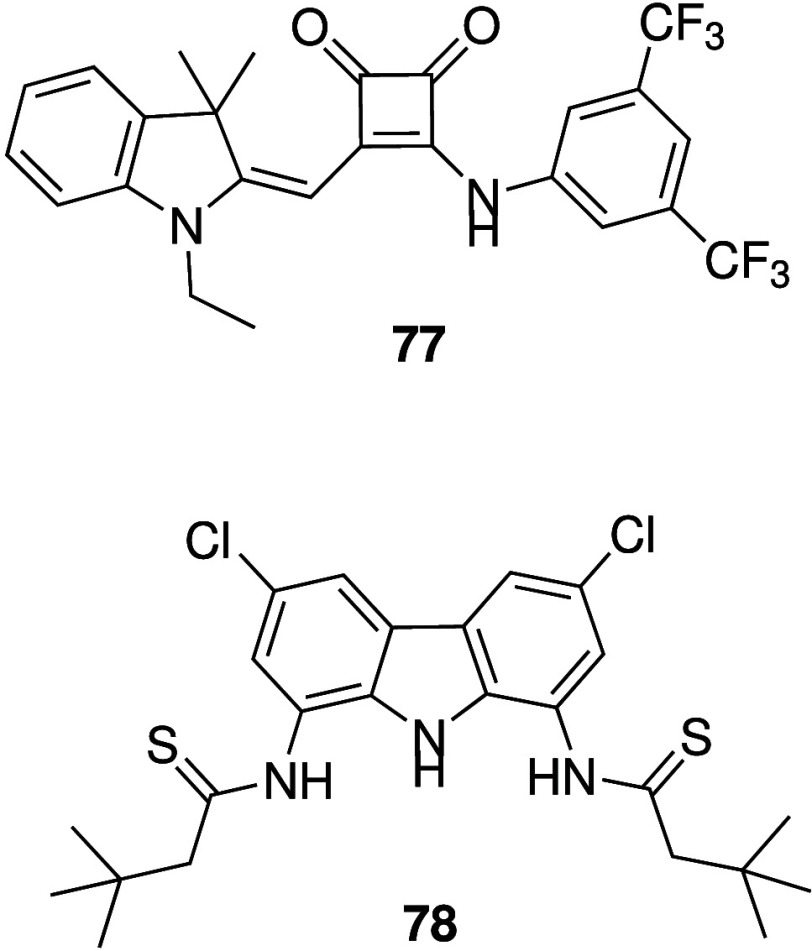
Various Anionophores with Antibacterial Activity **77**–**78**

The above-mentioned examples provide increasing evidence that synthetic anionophores can be used as antibacterial agents. At this point, most reports only show functionality in Gram-positive bacteria (which only have a single cell membrane) and not against Gram-negative bacteria (which have two cell membranes). Figuring out how to overcome the barrier of the outer membrane in Gram-negative bacteria and be able to use anionophores against these types of bacteria will be an important future direction. However, in order to determine which structural features or properties are most important for good antibacterial activity, it will be necessary to ensure that experiments (both liposome-based and bacterial assays) are uniform and performed under the same conditions so that results from different research groups can be compared. We recommend the use of MIC values (minimum inhibitory concentrations) obtained via broth microdilution methods starting with a bacterial concentration of 5 × 10^5^ CFU/mL, as this is most accepted by microbiologists.^295^

In addition to the above-mentioned transmembrane chloride transporters, there have been a few recent reports that suggest that increasing fluoride (F^–^) influx can also be a potential strategy for antibacterial and antibiofilm activity. In 2014, Breaker and co-workers showed that Gramicidin D, a well-known pore-forming antibiotic, can enhance the antibacterial activity of F^–^ against *B. subtilis* presumably due to transport of this anion through the Gramicidin D pore.^296^ Similarly, Silva and co-workers recently found that valinomycin and monensin are able to enhance the antibacterial activity of fluoride.^297^ Because valinomycin and monensin are better known for their cation transport (cationophore) ability, this result is somewhat surprising. However, there have been several reports of anion transport facilitated by these traditional cationophores,^298–301^ and the authors used molecular dynamics modeling to suggest that the observed F^–^ influx in bacteria is due to ion-pair transport facilitated by these ionophores. It is tempting to think that real anionophores (instead of cationophores) might be better suited for F^–^ transport in bacteria. Given the recent reports of synthetic molecules that have shown F^–^ transport in liposomes,^127–131^ this is certainly an avenue worth investigating. In this regard it is worth noting that a variety of disubstituted ureas have been shown to display antibacterial and antibiofilm activity in combination with fluoride.^302–305^ For example, Zhang, Wu and co-workers reported that fluorinated *N,N*-diphenylurea **79** (Scheme 26) can enhance the antibacterial and antibiofilm activity of fluoride against *S. aureus*. While the authors do not mention that this could be due to anionophore activity (they propose binding to a fluoride ion channel protein), Busschaert et al. have shown that compound **79** functions as a potent anion transporter in liposomes.^197^ It is therefore possible that the transmembrane anion activity of **79** is responsible for the observed synergistic effects between **79** and F^–^.

**Scheme 26:**
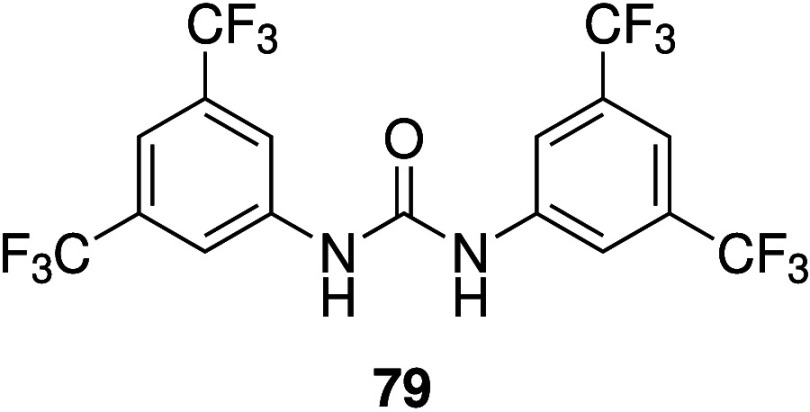
*N*,*N*′-Diphenylurea **79** with Putative Antibacterial and Antiviral Activity

#### 2.4.2 Antifungal Agents

Whereas the need for novel antibiotics is well appreciated, there is arguably an even greater need for novel antifungal agents. Mortality rates for invasive fungal infections are larger than for bacterial infections (often >50%), and approximately 1.5 million people die of invasive fungal infections every year.^306^ In addition, the development of antifungal agents is more challenging than the development of antibacterial agents, because fungi are eukaryotic cells and are more closely related to human cells, leading to challenges in selectivity. Currently, there are only 3 classes of systemic antifungal agents (the fourth class, fluoropyrimidines, is only used in combination therapies), and all of them have severe limitations with regards to pharmacokinetics, toxicity, target spectrum, or resistance.^307^ One of the oldest, and still most widely used, antifungal agents are the polyene macrolides (e.g., amphotericin B–Scheme 27). These are lipophilic macrocyclic compounds that bind to ergosterol and subsequently form pores in fungal membranes,^308^ leading to strong fungicidal activity (it has also been suggested that they can act as an ‘ergosterol sponge’, i.e. remove ergosterol from fungal membranes and thereby destabilize the membrane).^309,310^ Unfortunately, the selectivity for ergosterol (the fungal sterol) over cholesterol (the human sterol) is limited, leading to severe side effects and toxicity in humans. Much effort has therefore been put into improving the properties of the polyene macrolides.^311^ In general, this has focused on the preparation of Amphotericin B analogues or delivery systems with reduced toxicity. For example, Regen and co-workers reported that appending amphotericin B with one or more facial amphiphile (cholic acid) can reduce the hemolytic and cytotoxic behavior of amphotericin B, while retaining its antifungal activity.^312–314^

**Scheme 27:**
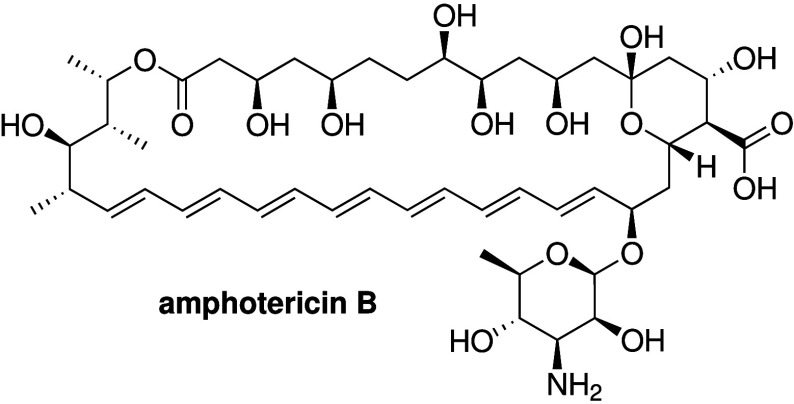
Structure of Amphotericin B

Given the fact that polyene macrolides form ion channels, it might be possible to develop synthetic anion transporters with antifungal activity. This is essentially a problem of creating transporters with membrane selectivity, or transporters that can be switched on locally at the site of infection. However, there are no examples of synthetic anion transporters that have been tested for antifungal activity and there is therefore great potential for the field to move in this direction. In addition, Li and Breaker have shown that the activity of antifungal agents that destabilize the cell membrane (amphotericin B and Novexatin) can be enhanced by the presence of F^–^,^315^ suggesting that both transmembrane Cl^–^ and F^–^ transporters have potential to treat fungal infections.

#### 2.4.3 Other Infectious Diseases

As the Covid-19 pandemic has clearly shown, there is a great need for potent antiviral drugs. At the moment, our best protection against viruses is to prevent infections using vaccination.^316^ Unfortunately, there are very few antiviral drugs that can provide a cure when a viral infection does occur (a notable exception is the cure for Hepatitis C).^317^ Because viruses are not living organisms with an active metabolism, it is extremely challenging to develop antiviral compounds. However, most human viruses have an external membrane,^318^ and many membrane-active natural products have previously been shown to possess antiviral activity. This includes the many natural cation- or anion-transporting molecules discussed in the previous sections, such as prodigiosins,^319^ amphotericin B,^320^ squalamine,^321^ and antimicrobial peptides.^322^ However, the exact mechanism of their antiviral activity is not always clear and it has not been shown that their ionophore or ion channel activity is the reason for the antiviral activity. There is thus great potential to investigate the effect of natural and synthetic anionophores on viral infections. As with all infectious diseases, one of the biggest challenges related to anionophores as potential antiviral drugs is selectivity. Viral membranes are derived from the host and membrane composition is therefore strongly related to the composition of human membranes. However, viral membranes display much more curvature and it has been suggested that this can provide a means to achieve selective membrane-targeting antiviral drugs.^323^ On the other hand, a recent report by Banerjee and co-workers suggested a new reason for why synthetic anionophores could be potential antiviral agents.^324^ An initial *in vitro* screening had identified *N,N*′-diphenylureas, especially those containing electron withdrawing groups such as CF_3_ (e.g., **79**, Scheme 26), as potential antiviral agents against IAV (influenza A) and SARS-CoV-2. Amazingly, *in vitro* antiviral activity was observed at concentrations <1 μM, whereas toxicity to human cells was only observed at much higher concentrations, and *in vivo* antiviral activity was also demonstrated in mice. Mechanistic studies showed that the viral particles still bind to cellular membranes, but uptake is inhibited. By examining other potential cargoes, it was shown that **79** specifically inhibits clathrin-mediated endocytosis and macropinocytosis. Because **79** has previously been shown to transport chloride across liposomal membranes,^197^ the authors tested changes in ion concentrations induced by **79** in A549 cells. It was shown that **79** can alter chloride homeostasis and leads to intravesicular chloride accumulation, and at the same time inhibits acidification of endosomes. The authors therefore hypothesized that the altered ion homeostasis induced by **79** interferes with the endocytosis process and **79** thus functions as an antiviral agent via endocytosis inhibition. While this is the only example that has shown this effect and the hypothesis will need to be verified, it suggests that there is potential in testing synthetic anionophores for antiviral activity, especially given the fact that other protonophores that can deacidify endosomes have also been suggested as antivirals (e.g., niclosamide, Scheme 28).^197^

**Scheme 28:**
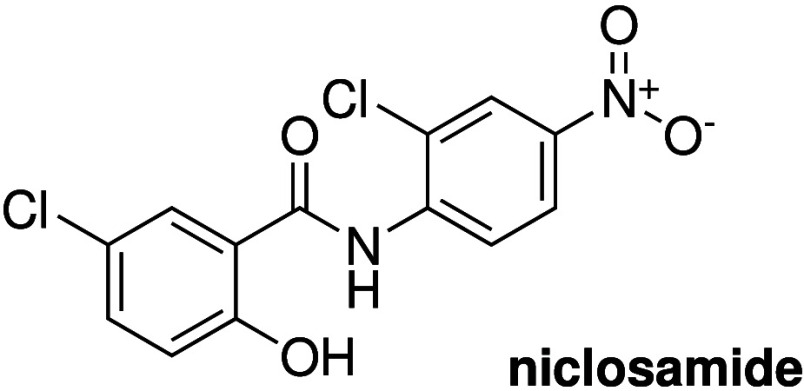
Structure of the Anthelmintic Drug Niclosamide

Arguably the most neglected infectious diseases are those caused by protozoa and other parasites (e.g., malaria, Chagas disease, Leishmaniasis), because they are most common in lower income countries. As with the other infectious diseases, antiprotozoal activity has been observed for natural products with known anionophore or ion channel ability, such as prodigiosins,^253^ tambjamines^325^ and amphotericin B.^326,327^ Synthetic anionophores, in contrast, have not been investigated yet and this represents another underexplored application of anion transporters. However, there is reason to believe that they could be useful in this regard. Niclosamide (Scheme 28), for example, is a commercial anthelmintic drug (treatment of parasitic worms) whose mode of action is most likely mitochondrial uncoupling.^328,329^ Gale and co-workers have recently shown that many anion transporters also function as mitochondrial uncouplers due to fatty acid flip-flop mediated H^+^ transport.^185–188^ Furthermore, niclosamide is a member of the salicylanilide family and a variety of salicylanilides have been shown to function as H^+^/Cl^–^ transporters by Talkudar and co-workers.^242^ Taken together, this information suggests that there is merit in testing the ability of anionophores (especially H^+^/Cl^–^ transporters) for their ability to treat these neglected diseases.

### 2.5 Drug and Probe Delivery

As is clear from the discussions above, most synthetic anionophores have been developed to transport chloride anions across lipid bilayers. However, there are many organic anions that cannot cross membranes unassisted and there is merit in developing synthetic systems that transport these anions as well. This could be useful for transporting anionic probes to facilitate cell imaging, or to transport anionic drugs. The latter can be particularly useful as the inability to reach the intracellular target is often one of the main bottlenecks in drug development. This problem can be expected to increase in the future, with the increasing importance of biologics (e.g., DNA/RNA-based drugs). Methods that can facilitate cellular uptake of these, often highly charged, biologics are therefore highly desired.

Probably the best-known systems that can facilitate the translocation of charged drugs across membranes are the cell penetrating peptides (CPPs). CPPs are small peptides that have been shown to help the uptake of various cargoes, ranging from small-molecule drugs, to nucleic acids, peptides, proteins and nanoparticles.^330–334^ While their mechanism of action is not always fully understood, it is generally believed that they work *via* either endocytosis or direct translocation. Endocytosis involves the internalization of compounds by engulfing them in plasma membrane and forming a vesicle. This is an energy-dependent cell-based uptake mechanism and does not really involve ‘membrane transport’ as defined throughout the rest of this review. On the other hand, direct translocation can involve the formation of pores in the membrane (e.g., barrel-stave model) or membrane destabilization (e.g., carpet model, and inverted micelle model), followed by translocation into the cell.^332^ Because this mechanism involves the translocation of a covalent or noncovalent peptide-drug complex through a lipid bilayer, it is more closely related to the transmembrane chloride transport facilitated by small molecules discussed in the previous sections. Peptides that are believed to function [partially] in this way are Penetratin and Tat.^335,336^ Although the study of CPPs is a large research field, it falls beyond the scope of this review, which focuses on synthetic anionophores. Readers are referred to the various reviews of CPPs that can be found in literature.

Given the success of CPPs, it is no surprise that some synthetic peptides with chloride transport ability have also been tested for their drug delivery properties. For example, Tomich and colleagues have used their peptides originally designed to mimic the second transmembrane segment of the α-subunit of the postsynaptic glycine receptor (GlyR–see section 2.1) for drug delivery. One of the channel-forming peptides (NC-1059: KKKKAA­RVGLGI­TTVLVT­TIGLGVRAA) was shown to also open paracellular pathways, resulting in an increase in the permeation of carboxyfluorescein, various FITC-labeled dextrans of different sizes and the anionic drug methotrexate across *in vitro* corneal epithelial monolayers (albeit to a small extent).^81,82,337^

Another anion transport group that has become interested in CPPs and drug delivery is Stefan Matile and co-workers. While working on channel-forming artificial β-barrels for chloride transport, they noticed that barrels appended with arginine (containing a positively charged guanidinium group) were selectively transporting *cations* over anions (Scheme 29).^338^ This was corroborated by results from other groups that had shown that arginine-rich peptides can form *cation* selective channels.^339–341^ Matile reasoned that the high p*K*_a_ of guanidinium implies that it is always positively charged and therefore always has strongly bound (but dynamic) counteranions for stability. These counteranions change the overall charge of the artificial β-barrels from positive to negative, explaining the cation over anion selectivity for transmembrane transport. Given that many CPPs are essentially polyarginines, Matile and co-workers investigated whether the drug delivery ability of CPPs could also be due to this ‘counterion effect’.^342,343^ They hypothesized that polyarginines are covered with counterions and bind to human membranes via counterion exchange with negatively charged lipids. This leads to accumulation on human membranes, followed by translocation inside the cell via transient pores formed by the peptides or other mechanisms (and release to the cytosol via another counterion exchange event). If this is true, the original counterion of the polyarginines CPPs should influence their drug delivery ability and Matile and co-workers found that amphiphilic pyrenebutyrate analogues were particularly good at improving drug delivery.^344–346^ Using this “pyrenebutyrate trick” the Matile group and others have been able to facilitate the uptake of fluorophores, peptides,^347^ proteins^348–350^ and bioactive oligonucleotides.^351,352^

**Scheme 29:**
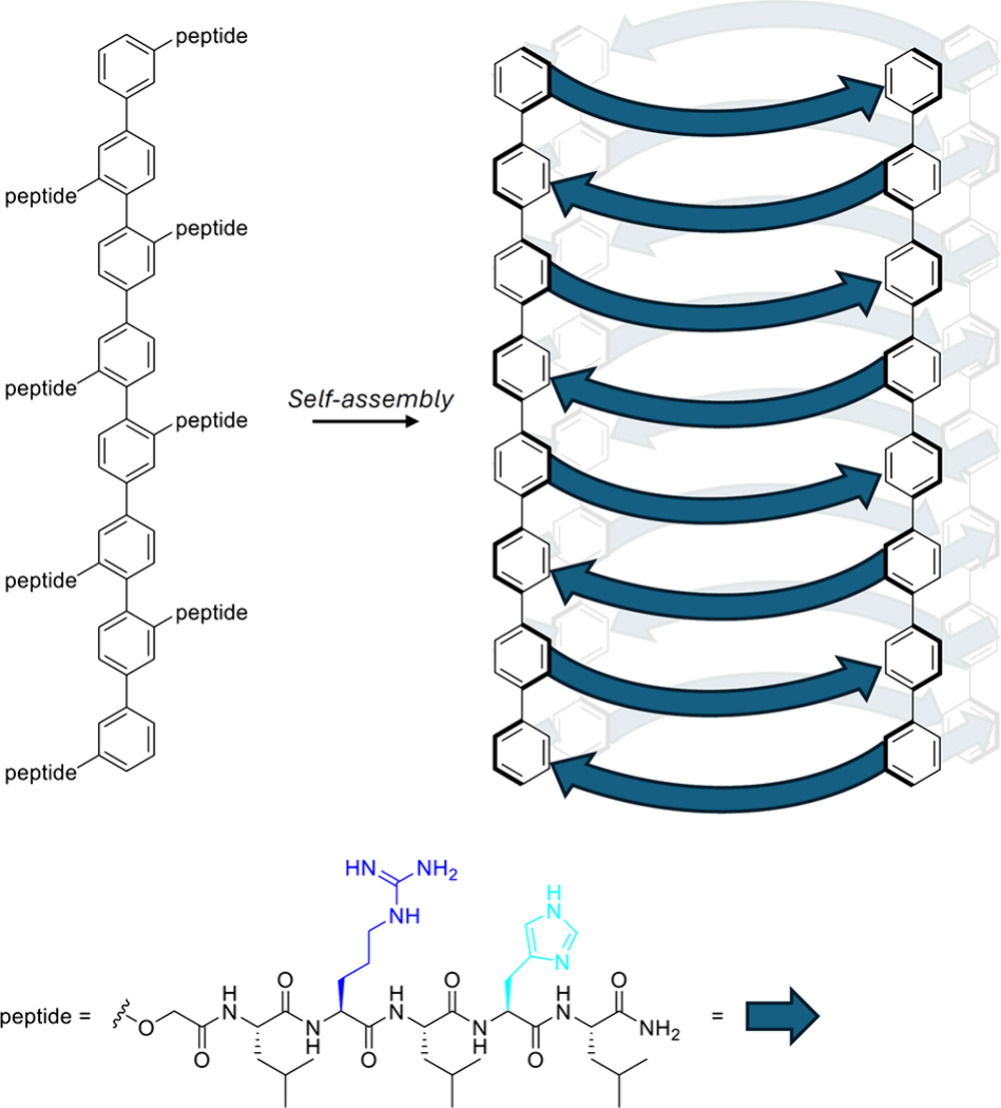
Scheme of the Self-Assembled Artificial β-Barrel Containing Arginine Residues Reported by Matile and Co-workers to Be a Cation-Selective Ion Channel

After their success with CPPs, the Matile group became interested in the thiol-mediated uptake of drugs, macromolecules and even viruses.^353–355^ Thiol-mediated uptake relates to a delivery method that involves dynamic covalent exchange chemistry with thiols present on the outside of cells. This attaches the transporter covalently to the cell and translocation to the inside of the cell can possibly be achieved via endocytosis, “walking” or “hopping” along disulfide tracks in membrane proteins, transient membrane defects or other unknown mechanisms. Release on the inside of the cell should be possible via exchange chemistry with glutathione or other sulfur species. Originally, the Matile group worked with polymeric cell-penetrating poly­(disulfides) (e.g., **80**, Scheme 30), which still have the guanidinium side-chains of CPPs.^356^ Similar poly­(disulfides) had already been used commonly for gene delivery,^353,357–361^ and Matile and others were able to use the poly­(disulfides) for the delivery of hydrophilic fluorophores,^362,363^ proteins,^364,365^ quantum dots,^366^ oligonucleotides,^367,368^ antibodies,^369^ CRISPR-Cas9 machinery,^370^ and drug-loaded nanoparticles^371,372^ into living cells. Later on, the group realized that a polymer is not necessary and that single disulfide residues are sufficient, provided that they are reactive enough, such as in asparagusic acid (**81**),^373^ epidithiodiketopiperazines (**82**),^374^ and benzopolysulfanes (**83**)^375^ (Scheme 30). In more recent work, they expanded this idea to other functionalities that can dynamically exchange with thiols (now called ‘cascade exchangers’), such as 1,2-diselenolanes (**84**),^376^ selenenylsulfides (**85**),^377^ certain Michael acceptors (**86**),^378,379^ and cyclic thiosulfonates (**87**)^380^ (Scheme 30). While their exact mechanism of entry differs (some transporters end up in the endosomes, some in the cytosol and some in the nucleus), they do have some features in common. Delivery is not inhibited by standard endocytosis inhibitors, but is usually affected by reagents that can react with thiols (e.g., Ellman’s reagent). This shows that all these transporters use thiol-mediated uptake via reactions with exofacial thiols on various transmembrane proteins. This delivery mechanism appears very powerful and Matile and others have been able to use this method for the delivery of peptides,^381^ PNA,^382^ proteins,^383^ quantum dots,^383,384^ liposomes^385^ and polymersomes.^386^ Interestingly, many viruses (e.g., HIV)^387^ also use thiol-mediated uptake for entry into cells and many of the aforementioned compounds could therefore compete with viruses for exofacial thiols and thus inhibit viral entry.^380,388^

**Scheme 30:**
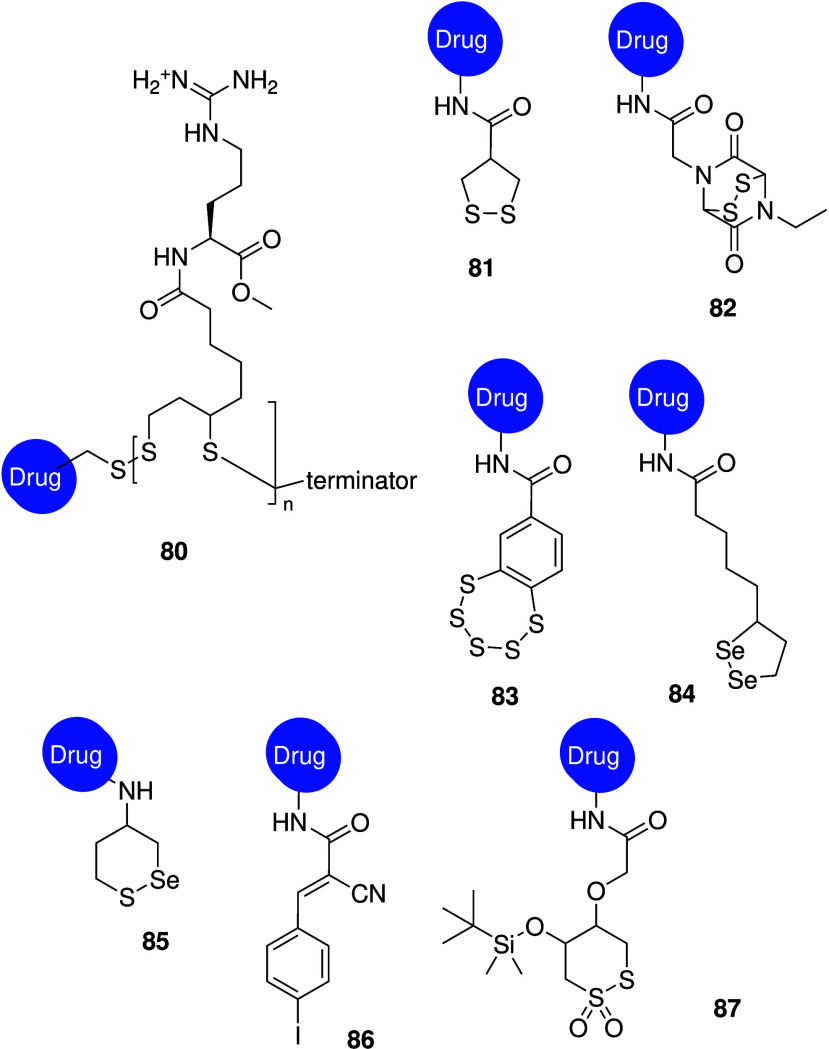
Tethers for Thiol-Mediated Uptake of Drugs Developed by Matile and Co-workers

Disulfides do not only have potential for thiol-mediated uptake, but are also a useful handle to covalently link cargos to potential carriers via a cleavable linker. Regen and co-workers have extensively used this method to use “molecular umbrellas” for the transmembrane transport a various hydrophilic species.^389–391^ In their molecular umbrella design, a hydrophilic moiety is tethered to a scaffold containing two, four or eight steroid groups as facial amphiphiles (Scheme 31). In a nonpolar environment such as the membrane, the umbrellas are expected to adopt a shielded conformation whereby the hydrophilic cargo is protected from the environment by the sterols. On the other hand, in an aqueous environment, the umbrellas flip and expose the hydrophilic cargo and the polar side of the sterol to the environment. Transmembrane transport is therefore thought to occur by flipping between these two conformations depending on the environment. Once across the membrane, the umbrella is in its exposed conformation, and the disulfide linker is available for cleavage by glutathione. Using this concept, Regen and others have been able to facilitate the transport of small-molecule drugs,^392^ AMP and ATP,^134,393^ peptides^394–396^ and oligonucleotides.^397,398^ It might be tempting to think that these umbrellas function by thiol-mediated uptake. However, Matile and co-workers had shown that single disulfides without ring strain are not good substrates for thiol mediated uptake. Furthermore, most of Regen’s experiments were conducted in liposomes which do not have any thiols or proteins able to facilitate thiol-mediated uptake, and the molecular umbrella mechanism of transport is therefore more likely. In some of their later work, Regen and colleagues have been able to use a single molecular umbrella (containing 8 sterol amphiphiles) to facilitate the delivery of highly anionic siRNA *in vitro* in HEK293 cells and *in vivo* using intravitreal injection in rats.^399^

**Scheme 31:**
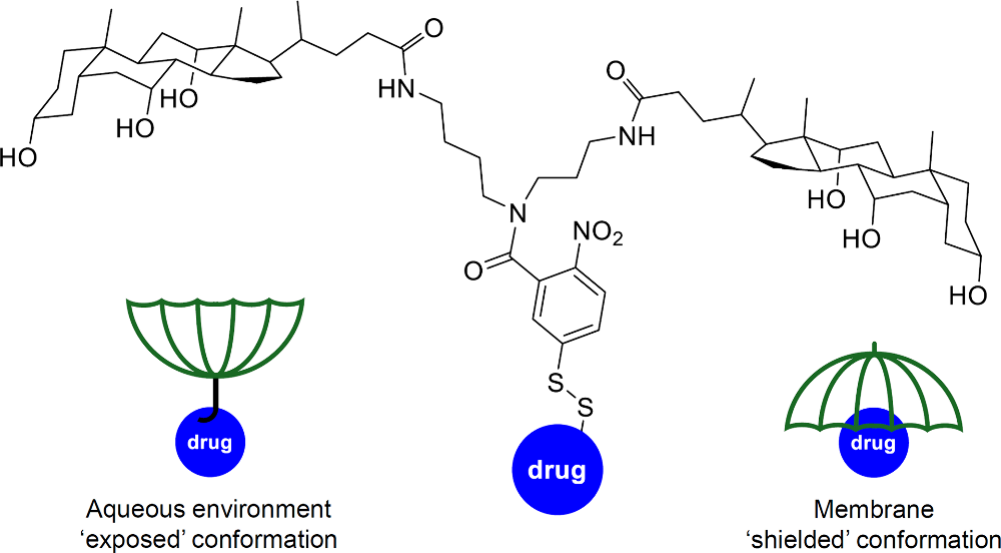
Regen’s Molecular Umbrellas

Most CPPs, as well as the thiol-mediate uptake compounds and molecular umbrellas, require covalent attachment of the delivery agent to the drug or macromolecule that needs to be transported into the cell. Examples of self-assembled or unimolecular ion channels or carriers that involve noncovalent interactions with their target, have been much less explored for the delivery of anionic drugs. Technically, lipophilic counter cations of anionic drugs,^400–403^ as well as the many cationic lipids^404–407^ and cationic polymers^408–411^ used for gene delivery can be classified in this category. However, they rely solely on nonspecific Coulombic interactions and hydrophobic effects, and usually function via endocytosis. Readers are therefore referred to reviews on these specific delivery systems for more information.^412–415^ More “supramolecular chemistry” examples of this polycation approach for oligonucleotide or gene delivery include the use of various cationic calixarenes and cyclodextrins,^416–430^ Matile’s dynamic amphiphiles (e.g., **88**),^431^ an octaarginine appended molecular umbrella (**89**),^432^ and a variety of the ‘Schmuck cation’-based gene delivery systems (**90**) (Scheme 32).^433–439^

**Scheme 32:**
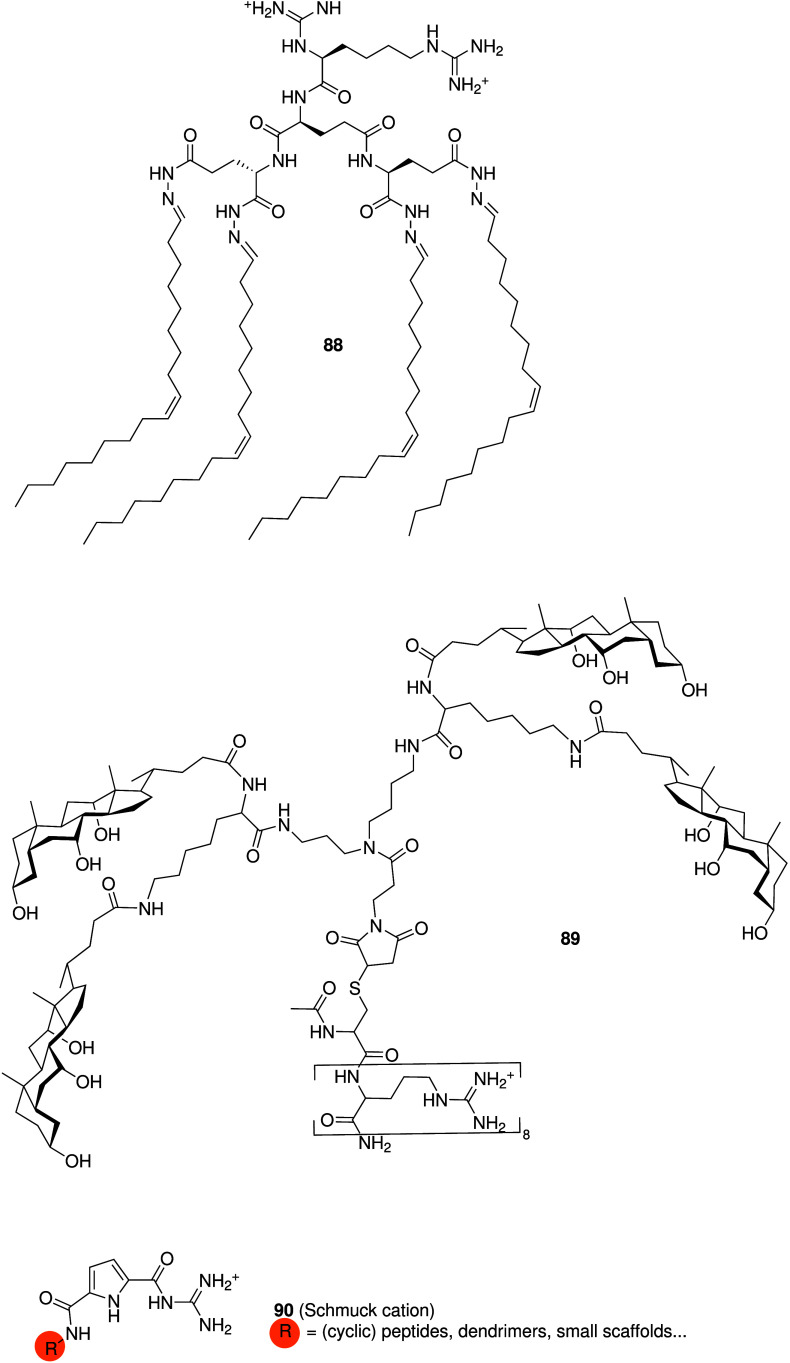
Polycations Used for Gene Delivery

There are only a few examples of anionic drug or gene delivery by synthetic anionophores comparable with the ones reported in the previous sections. Eskandani et al. have reported a unimolecular ion channel based on PEGylated β-cyclodextrin that was able to facilitate the transmembrane movement of single stranded DNA across planar lipid bilayers (**91**, Scheme 33), suggesting that the internal cavity is large enough for single-stranded DNA to pass through.^440^

**Scheme 33:**
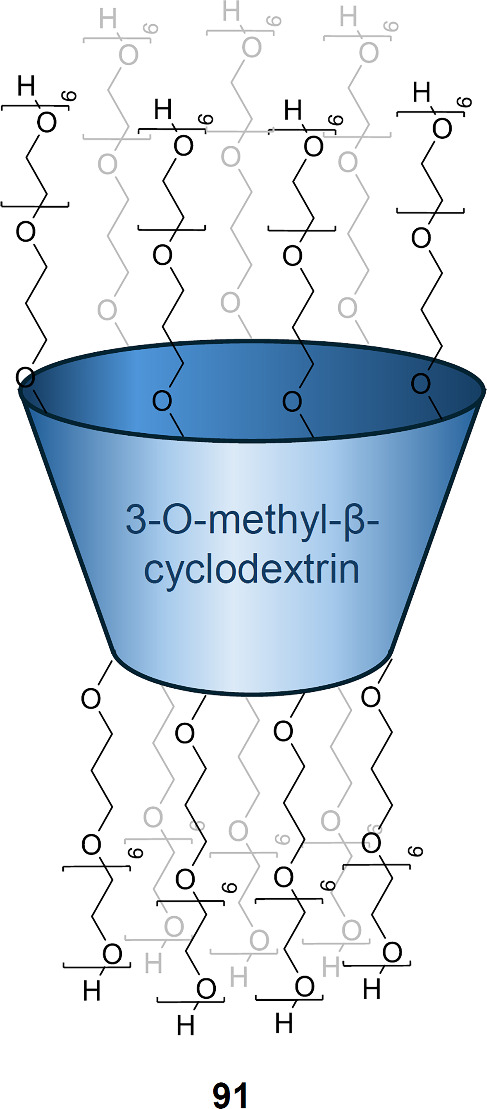
Unimolecular β-Cyclodextrin Channel **91** for ssDNA Transport

A few small molecules have also been reported for the delivery of anionic drugs. In 2008, Hamachi and co-workers showed that lipophilic compounds containing Zn-dpa (2,2′-dipicolylamine) recognition motifs were able to facilitate the uptake of phosphorylated peptides and fluorophores into HeLa cells (**92**, Scheme 34).^441^ Mechanistic studies suggested that uptake occurred mainly through endocytosis. Presumable **92** forms complexes with the phosphorylated peptides and coat them with hydrophobic residues. These hydrophobic residues ensure binding to the membrane, followed by endocytosis. A few years later, in 2012, Gokel and co-workers showed that certain isophthalamide and dipicolinamide derivatives (e.g., **93**, Scheme 34) are able to transport small (2.6 kb) and large (>20 kb) plasmids into competent JM109 *E. coli* cells.^442^ Given that isophthalamide derivatives are established transmembrane chloride transporters,^87,443–447^ these results show promise for the use of many of the chloride transporters discussed in the previous section for the delivery of anionic drugs and even DNA into cells.

**Scheme 34:**
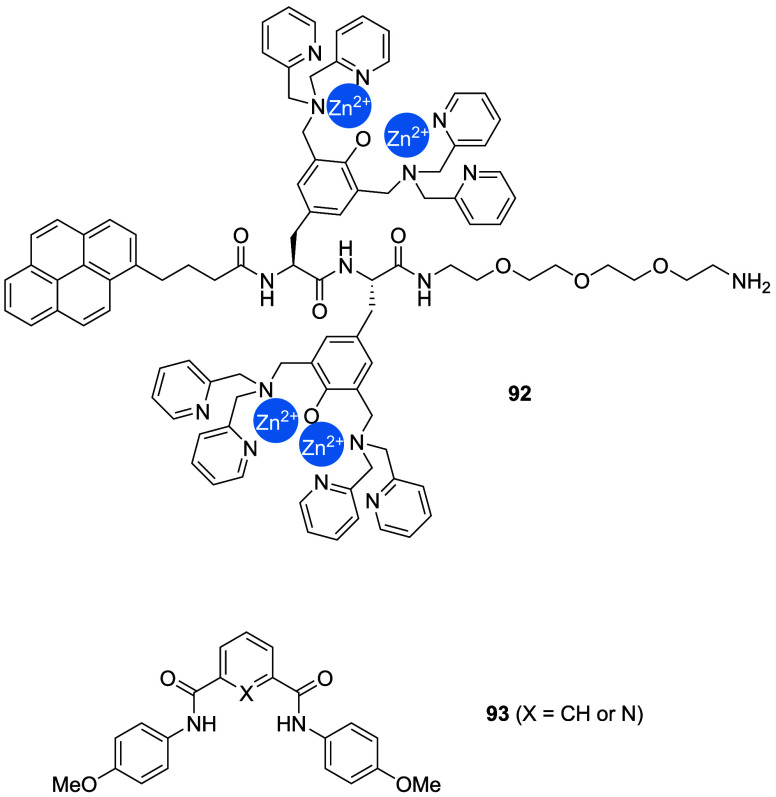
Small Molecule Transporters for Phosphorylated Peptides (**92**) or Plasmid DNA (**93**)

In addition to highly charged phosphorylated species such as DNA, hydrophilic small molecule drugs with negative charges can also suffer from limited membrane permeability and therefore limited bioavailability. In recent years, a number of groups have therefore reported on the ability of synthetic anionophores to transport organic anions, including a variety of anionic drugs. In 2020, Chmielewski and co-workers reported that their carbazole-based anionophore (e.g., **78**, Scheme 25) is able to transport aspirin and other organic anions across the membranes of 7:3 POPC:cholesterol liposomes.^133^ Soon thereafter, Busschaert and co-workers showed that simple *N,N*-diphenylureas (e.g., **94**, Scheme 35) are able to increase the membrane permeability of a variety of small-molecule anionic drugs at concentrations where they do not facilitate chloride transport.^448^ A total of 14 drugs were investigated (amoxicillin, aspirin, bezafibrate, carbenicillin, diatrizoate, furosemide, gemfibrozil, ketorolac, naproxen, penicillin G, salicylate, tolmetin, valproate, valsartan), the majority of which could be transported by the ureas. More recently, Torres-Huerta and Valkenier demonstrated that other synthetic anionophores (such as **95** and **96**, Scheme 35) are also able to transport organic carboxylates, including salicylate (2-hydroxybenzoate) across lipid bilayers.^136^ The three aforementioned examples have so far only shown the possibility of transporting drugs across liposomes. For real-life applications it will be necessary to investigate whether synthetic anionophores can also increase the uptake of anionic drugs in cells. Given the fact that a number of synthetic anion transporters have already been shown to transport other types of carboxylates,^101,169,449–451^ it is conceivable that such a molecule can be found. These anionophores could then find applications as excipients in drug formulations to increase the uptake of hydrophilic anionic drugs, or as a stimuli-responsive drug delivery system by incorporating a switchable anion transporter into liposomes encapsulating a hydrophilic drug.

**Scheme 35:**
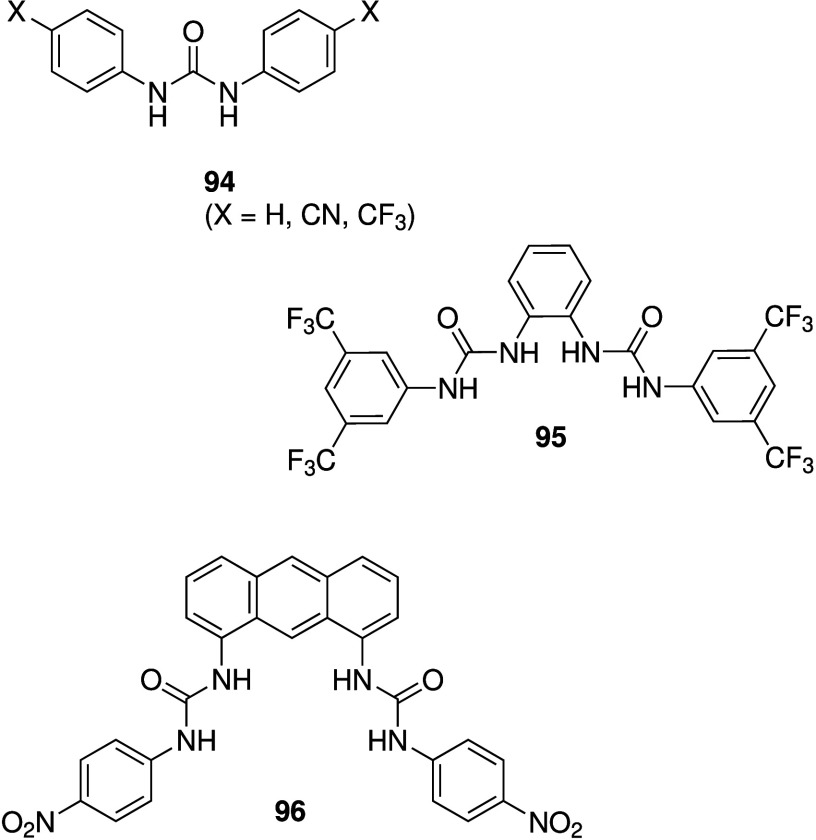
Small Molecule Transporters **94**–**96** for Anionic Drugs

In many drug delivery papers, experiments are performed with fluorescent probes as a model for a hydrophilic drug (including the anionic probe carboxyfluorescein). However, probes play a fundamental role in biological research to investigate cellular pathways, mechanism of drugs, etc. Like any molecule, if the probes are too hydrophilic, they cannot cross the cell membrane and their use will be limited. This can potentially be overcome using prodrugs or physical methods such as microinjection or osmotic stress. However, these methods are not without drawbacks and the use of synthetic transporters for such probes could prove to be beneficial. This can be beautifully demonstrated by the work of Kraus and co-workers.^452^ They developed a synthetic transporter for the intracellular delivery of highly anionic fluorescently labeled nucleoside triphosphates. Their design was based on appending per-6-amino-β-cyclodextrin, which can strongly bind to ATP, with an octaarginine-type CPP (**97**, Scheme 36). It was shown that **97** is able to transport fluorescently labeled dUTP in a variety of human cell lines as well as bacteria, and that this transport was not affected by the presence of other organic anions. Furthermore, they were able to show that the delivered fluorescently labeled dUTP is incorporated into DNA and can be used for cell-cycle analysis. A similar approach was used by Mascareñas, Montenegro and colleagues for the intracellular delivery of a variety of anionic probes.^453^ They tethered a cationic organic cage that had previously been shown to bind to pyranine^454–456^ with a tetra-arginine CPP-like peptide (**98**, Scheme 36). This new cage was shown to facilitate the intracellular delivery of pyranine and various sulfonated Alexa dyes in human cell lines. In addition, the delivery of the highly anionic pyranine dye allowed for the determination of the pH of the cytosol (7.5), early endosomes (7.0) and late endosomes (6.5), highlighting the usefulness of transporters for anionic probes. Another small-molecule transporter for pyranine has recently been reported by Yongju Kim and co-workers.^457^ Bisimidazole compound **99** (Scheme 36) was shown to form vesicles in aqueous solutions in the presence of pyranine due to Coulombic interactions and π-π stacking between **99** and the anionic dye. Confocal microscopy revealed that these vesicles enhanced the uptake of pyranine into MCF-7 cells.

**Scheme 36:**
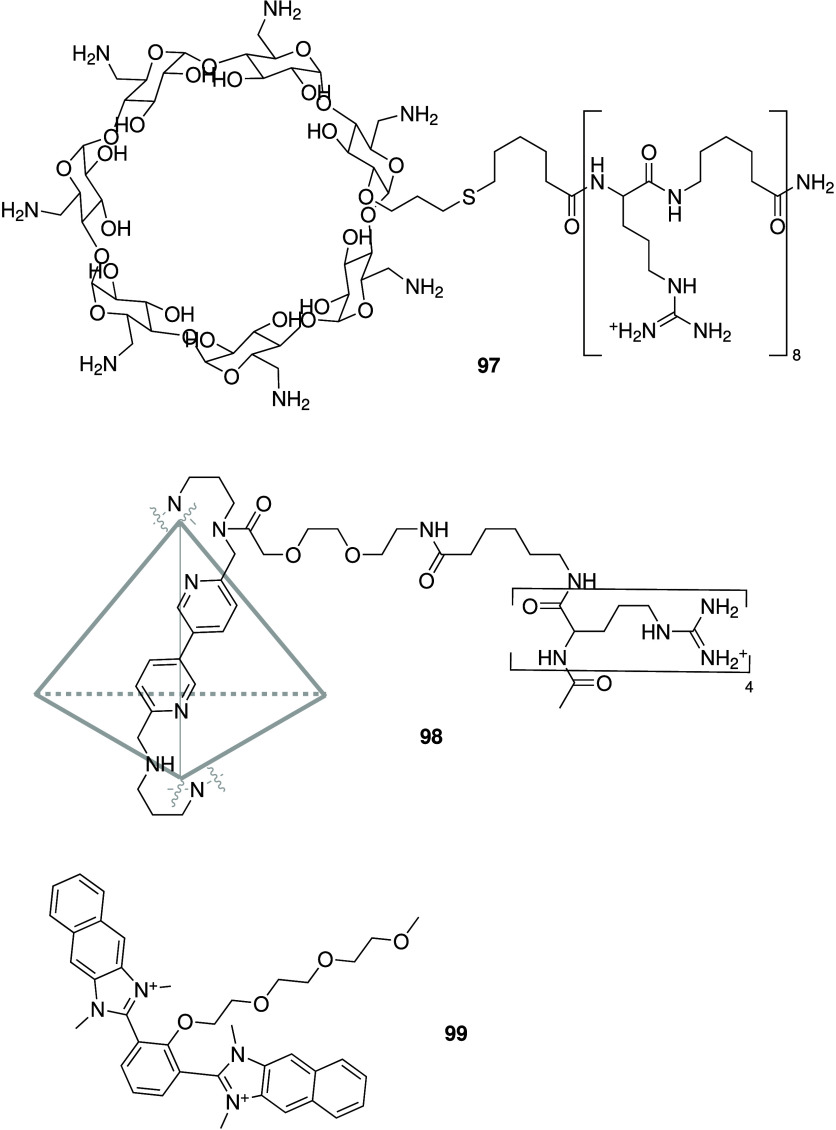
Transporters **97**–**99** for Fluorescent Probes

An entirely different approach on the use of ionophores for imaging was recently reported by Langton and Davis.^458^ They reported MRI contrast agents that were encapsulated in POPC-cholesterol liposomes, which turns down the water flux and thus contrast. However, the addition of valinomycin and a *tren*-based tris-thiourea can restore the water flux due to KCl transport, and this leads to changes in image contrast. Even though we are still far from commercial applications, the aforementioned examples demonstrate that the therapeutic use of synthetic anionophores extends beyond the treatment of channelopathies, and includes potential anticancer, antibacterial, antiviral and drug/probe delivery applications. From the discussion above, we presume that anticancer/antibacterial/antiviral applications benefit from H^+^/Cl^–^ transport due to the increased toxicity of changing pH gradients in cells, while cystic fibrosis therapeutics would benefit from electrogenic Cl^–^ transport that does not disrupt cellular pH. Furthermore, the treatment of other channelopathies and drug/probe delivery applications will require anionophores that can transport anions other than chloride, preferentially without altering cellular pH and Cl^–^ concentrations.

## 3 Nonmedical Applications

### 3.1 Biomedical Research and Chemical Biology

In some of the early papers on anion transport mediated by synthetic small molecules, it was already noted that synthetic anionophores could find use in biomedical research.^12,459^ This was mostly inspired by the widespread use of cationophores, such as valinomycin, in biological research. Valinomycin is a selective electrogenic K^+^ transporting antibiotic that is often used in biochemical research due to its known ability to change K^+^ concentrations and alter membrane potentials.^460–463^ For anions, the most widespread used ionophore in biomedical research is tributyltin-chloride. This compound is an electroneutral Cl^–^/OH^–^ exchanger^464,465^ and is therefore combined with an electroneutral cation exchanger such as nigericin (a Na^+^/H^+^ or K^+^/H^+^ exchanger).^466^ The combination nigericin/tribultyltin-chloride is often used to alter intracellular chloride concentrations in order to calibrate fluorophores for the determinations of intracellular chloride concentrations.^467–469^ However, tributyltin-chloride is also a known toxic environmental pollutant^470,471^ and is therefore not an ideal candidate as a biomedical research tool. Furthermore, tributyltin-chloride is an electroneutral transporter and an electrogenic chloride transporter is therefore still missing. An electrogenic chloride transporter would allow altering the membrane potential without affecting metal cation concentrations or pH, which then allows the study of the effect of altering chloride concentrations on various cellular pathways or can be used in the study of natural ion channels.

This gap has led to a quest for a “valinomycin for chloride”.^299,472^ In their seminal paper, Davis and Gale developed a number of new assays to determine if a synthetic anion transporter functions as an electroneutral or electrogenic transporter, and if it can selectively transport Cl^–^ over OH^–^ (or H^+^).^184,473^ For any application of anionophores it is paramount to know the exact transport mechanism and selectivity, and we therefore recommend anyone in the field to perform these standard assays. They discovered that most synthetic anion transporters also facilitate electrogenic H^+^ transport (via protonation/deprotonation events) or OH^–^ transport (via complexation of OH^–^). However, the group was able to identify some transporters with the desired Cl^–^ over OH^–^/H^+^ selectivity, especially compounds with less acidic hydrogen bond donors and efficient chloride encapsulations (e.g., **100**, Scheme 37). **100** was also shown to transport I^–^ in Fischer rat thyroid cells, but it did not show deacidification of lysosomes and showed only mild cytotoxicity (IC_50_ = 43 μM) in human A549 cells, consistent with its Cl^–^ over OH^–^/H^+^ selectivity. Unfortunately, further biochemical studies on the effect of the anion transport facilitated by **100** on cellular function has not yet been reported. Since this paper, there have been a few reports of other electrogenic anionophores with good Cl^–^ over OH^–^/H^+^ selectivity, most notably some of Langton’s halogen and chalcogen bonding anionophores (e.g., **101** and **102**, Scheme 37).^474,475^ These studies have also shown that the Cl^–^ over OH^–^/H^+^ selectivity can be dependent on the lipid composition (in this case membrane thickness) and therefore more studies will be needed to find synthetic anionophore that are pure electrogenic Cl^–^ transporters under various conditions. Furthermore, the biological activity of **101** and **102** has not yet been investigated and it will remain to be seen if any of these synthetic transporters will be useful for biomedical research.

**Scheme 37:**
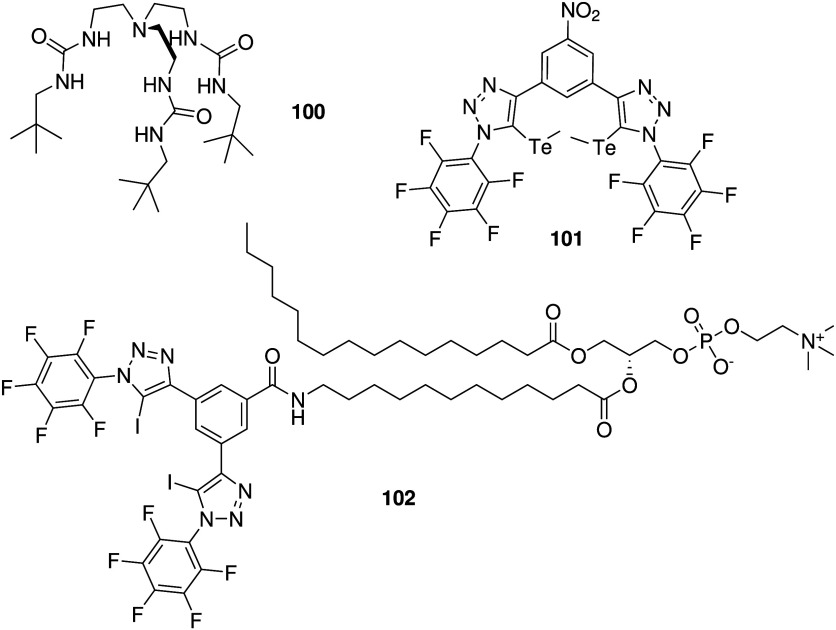
Electrogenic Cl^–^ Transporters **100**–**102** with Good Cl^–^ > OH^–^(H^+^) Selectivity

One of the reasons to use synthetic anionophores as biochemical research tools is to establish the downstream effects of changing intracellular chloride concentrations. Most studies on the cellular effects of anionophores have focused on determining the anticancer mechanism of these compounds (see section 2.3). To the best of our knowledge, there have been only 2 reports that studied in more detail the downstream cellular and physiological effects of synthetic transmembrane anion transporters. Suzuki and co-workers tested the effect of Tomich’s chloride transporting peptide (**2**, NK_4_-M2GlyR, see Scheme 1) on microperfused rabbit renal proximal tubules.^476^ They found that the change in Cl^–^ conductance caused by the peptide also caused pronounced fluid reabsorption via a transcellular route, as well as an increase in cation (Na^+^ and Ca^2+^) translocation via a paracellular route. These results suggest that chloride gradients could be important as an osmotic driving force. The other report came from Yang and co-workers on the effect of their α-aminoxy chloride channel **6** (see Scheme 2).^477^ It was shown that **6** is able to alter the membrane potential of MDCK cells, presumably due to its chloride transport ability. Because the membrane potential has an effect on a variety of cellular functions through voltage-gated ion channels, Yang and co-workers investigated the downstream effect of **6**. They performed a series of tests on rat thoracic aortic smooth muscle cells. When the cells were exposed to a solution with a high K^+^ concentration, the cells were depolarized and the intracellular Ca^2+^ concentration increased through the action of voltage-gated Ca^2+^ channels. Subsequent addition of **6** led to repolarization of the cells (due to chloride transport) and a decrease in intracellular Ca^2+^ concentration (due to deactivation of voltage-gated Ca^2+^ channels induced by changing the membrane potential). Because smooth muscle contraction is linked to Ca^2+^ concentrations, they also performed similar experiments with mouse thoracic aortic rings. First, the model organ was constricted with a solution containing high K^+^ concentrations, and it was shown that the subsequent addition of **6** led to complete relaxation of the aortic ring. Interestingly, both reports showed a change in Ca^2+^ concentrations as a result of chloride transport. Furthermore, tributyltin-chloride has also been shown to alter intracellular Ca^2+^ concentrations,^478,479^ indicating that this might be a general effect of chloride transport.

As there are only 2 reports of the use of synthetic anionophores in biochemical studies, there is much room for further exploration. We therefore encourage researchers working in this field to establish collaborations with biochemists, chemical biologists or other scientists who are interested in studying the effect of altering chloride concentrations. Given that chloride has been suggested to function as a secondary messenger^480^ and altered chloride homeostasis has been observed in conditions other than channelopathies,^481–483^ there could be much to discover. As a final case to illustrate the potential use of anion transporters on fundamental research, consider the case of optogenetics.^484–486^ Originally, optogenetics involved the genetically engineering of certain neuronal cells to express specific opsins, which are a class of light-activatable ion channels or pumps. In general, cation-selective channel rhodopsins are used to excite neurons and anion-selective halorhodopsins are used to inhibit neurons. This technique thus allows spatial and temporal control of neuronal activity and this has helped to understand some of the neural circuit problems in depression, Parkinson’s disease, and other neurological or psychiatric conditions.^487–489^ While the field has now expanded to other cell types and include other types of engineered light-switchable proteins,^490–492^ the story of optogenetics proves the power that control of ion transport can have on fundamental research.

### 3.2 Artificial Cells and Biomimicry

Artificial cells (sometimes also called protocells) are one of the major goals of synthetic biology. In the top-down approach, one of the aims is to find a minimal cell by knocking down genes to create the minimal genome necessary for life.^493,494^ In the bottom-up approach, the aim is to generate artificial cells from scratch, trying to mimic particular cellular functions in a synthetic system and then combining them together.^495–497^ Such artificial cells could provide information about the origin of life,^498–500^ help understand particular cellular functions,^501^ or could be used as therapeutics (e.g., drug delivery),^502,503^ molecular robots,^504,505^ nanoreactors/nanofactories (see also section 3.4)^506^ or biosensors (see also section 3.3).^507–509^ While there are several definitions of artificial cells, compartmentalization is always an essential part of the definition.^510–512^ This implies that artificial cells should have a defined boundary (i.e., membrane) and potentially have internal organelle-like structures. However, this should not just be a passive membrane. Biological membranes are specialized systems, that are made semipermeable through many membrane-bound proteins to allow nutrient flux, ion gradients and signal transduction. An important part of bottom-up synthetic biology is therefore to generate functional membranes.^513–515^ In most synthetic biology cells this is achieved using biological building blocks, such as phospholipids combined with pore-forming proteins (e.g., α-hemolysin)^516^ or DNA nanopores.^517^ However, there is merit in building artificial cells from entirely abiotic synthetic systems.^518,519^ Such abiotic systems could provide functionality not found in and orthogonal to biological systems, and are often more stable and cheaper than biological building blocks.

It is not hard to see the parallel between bottom-up synthetic biology and the pursuit for synthetic transmembrane anion transporters. In essence, every synthetic anionophore tested in liposomes is trying to mimic a biological function and could thus be considered a [very] minimal artificial cell. However, most anionophores only allow uncontrollable, passive transport of anions along the electrochemical gradient. Biological protein transporters, on the other hand, are tightly regulated. Their transport is directional and can be along the electrochemical gradient (ion channels, passive transport), or against the gradient (ion pumps, requires energy).^14^ Furthermore, most transport proteins and ion channels are gated and can be switched on and off with various triggers, such as pH, voltage, volume, light, ligands, or other ions (most notably Ca^2+^).^520,521^ Synthetic systems that can mimic this gated transport activity are therefore better suited to be incorporated into artificial cells. As a result, there has been an increasing interest in the development of switchable anionophores in recent years, as exemplified by the reviews dedicated to the subject.^522–524^ As mentioned above, the quest for gated or switchable anion transporters is often driven by a desire for selectivity in anticancer activity, but is also useful for the development of artificial cells. In the sections below we will therefore focus on reports of switchable anion transporters without specific biological activity, and focus on the biomimicry aspect.

#### 3.2.1 pH-Dependent Synthetic Anion Transporters

One of the most commonly used triggers to achieve switchable anion transport is pH.^525^ This has largely been driven by the different pH homeostasis in cancer cells versus healthy cells, but could also provide interesting pH gated synthetic anion transporters for artificial cell applications. Many synthetic anion transporters use nitrogen-based hydrogen bond donors and pH switching could therefore occur due to (de)­protonation of these nitrogen atoms. By fine-tuning the p*K*_a_ of the compound, switching can be achieved within a physiological relevant region (pH 5–8). In general, there are two methods to achieve this switching. In the first method, an anionophore with NH or OH hydrogen bond donors can be deprotonated at high pH. Such compounds will be active and neutral at low pH, but become negatively charged and thus inactive at high pH due to charge repulsion. This type of pH switch has been observed in (oxo)­thiosquaramides (e.g., **103**),^217,526^ amidosquaramides (e.g., **104**),^527^ diamidocarbazoles (**105**),^292^ phenol-containing isopthalamides (**106**),^444^ sulfonamides (e.g., **107**),^228^ and carboxylate-gated anion channel **108** (Scheme 38).^528^ Interestingly, in the case of **107** and **108** the deprotonated anionic compound was able to transport K^+^ instead of Cl^–^ at high pH (and *vice versa* at low pH).

**Scheme 38:**
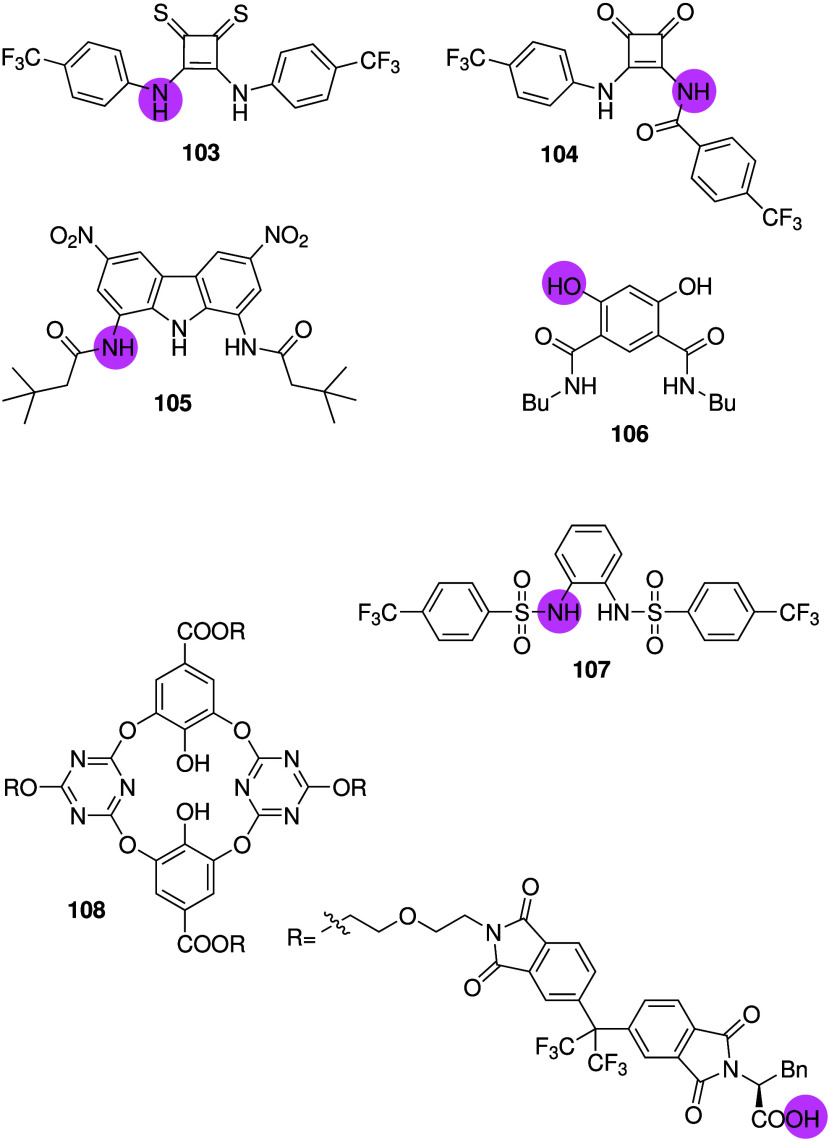
Structures of Anionophores Whose Activity Can Be Switched off at High pH Due to Deprotonation; The Putative Deprotonation Site Is Highlighted in Magenta

The second method for pH switching is the use of protonatable nitrogen atoms. In this approach, compounds are neutral and inactive at high pH, but become positively charged and active when protonated at low pH. Examples of this approach include phenylthiosemicarbazones (e.g., **109**),^218^ Talukdar’s triazine cages (e.g., **110**),^529^ and aminocyclodextrin channel **111** ^530^ (Scheme 39). It must be noted, however, that for compounds with multiple protonatable sites such as **110** and **111** pH-switching is more complicated. In this case, there will be multiple positive charges on the transporter at low pH, which can reduce transport ability because the transporter becomes too polar or because anion binding becomes too strong. As a result, the anion transport ability of **110** was optimal at pH 7.0, and decreased going toward higher and lower pH. On the other hand, for **111** anion transport ability was best at high pH (pH 10) and gradually decreased as the pH decreased.

**Scheme 39:**
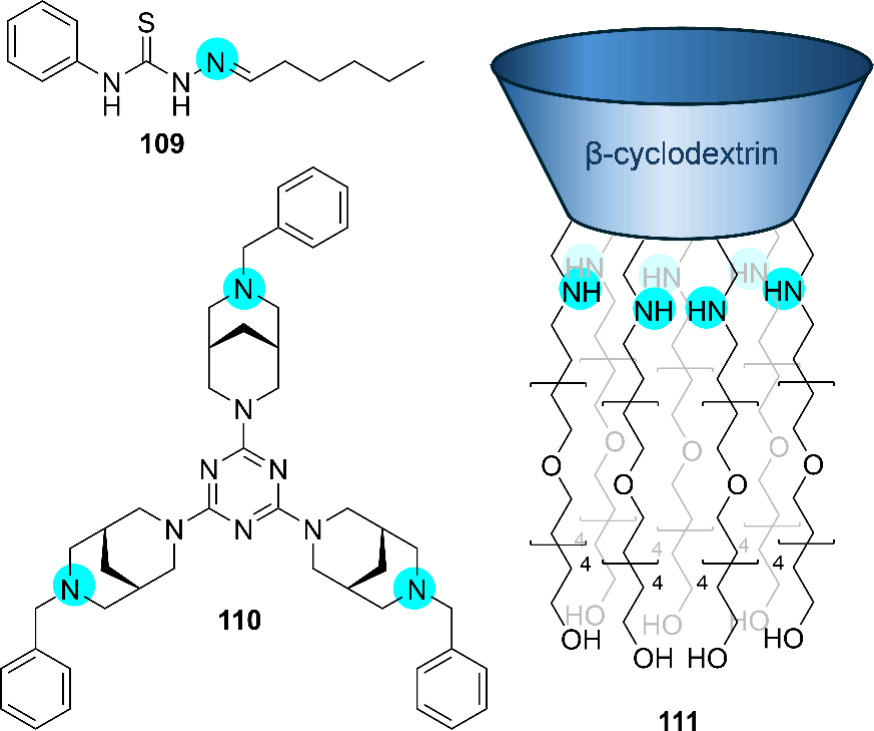
Structures of Anionophores Whose Activity Is Regulated by Protonation of Basic Nitrogen Atoms; Putative Protonation Sites Are Highlighted in Cyan

#### 3.2.2 Light-Activatable Synthetic Anionophores

Another commonly used method to switch the anion transport activity of synthetic anionophores is light. Light has the advantage that it is completely bio-orthogonal and that it can provide great spatiotemporal control over the switchable transporter. Given the success of the protein-based light-switchable ion channels and pumps in optogenetics (see section 3.1), it is certainly an interesting biological property to mimic with synthetic systems. Probably the best-known molecular photoswitch is azobenzene and its derivatives. Upon irradiation with the correct wavelength, azobenzene can undergo an (*E*)-to-(*Z*) isomerization resulting in a large overall change in conformation. Furthermore, the properties of the switch can be easily optimized by the addition of substituents or by replacing one of the aromatic rings with heteroaromatic rings. It therefore comes as no surprise that there are many photoswitchable anionophores reported based on the azobenzene scaffold. For example, Jog and Gin tethered an azobenzene unit to the upper rim of their β-cyclodextrin ion channel **111** (Scheme 39) to function as a gate.^531^ In the relaxed (*E*) conformation, the azobenzene unit is bound inside the β-cyclodextrin cavity and anion transport is diminished. Upon switching to (*Z*)-azobenzene upon irradiation, the azobenzene unit dissociates and the channel opens. The groups of Kyu-Sung Jeong and Matthew Langton have independently worked on anionophores containing a central azobenzene unit appended with (thio)­ureas^532^ or squaramides^224,225^ (e.g., **112**, Scheme 40). Only in the (*Z*) form can the tethered groups cooperatively bind to anions, which increases transmembrane transport ability. One of the best compounds reported in this series, **112**, showed an 8x enhancement of chloride transport by the (*Z*) isomer over the (*E*) isomer and could be reversibly switched ON and OFF by alternating irradiation with red (625 nm) and blue (455 nm) light. The Langton group have also incorporated azobenzene units in their relay-based anion transport systems.^18,533,534^ Talukdar and co-workers have reported a number of “opposite” azobenzene switches, where the (*E*) isomer is more active than the (*Z*) isomer. In one system, an azobenzene appended with amide functionalities (**113**, Scheme 40) was shown to bind and transport anions better in the planar (*E*) conformation than the (*Z*) conformation.^220^ In another report, they showed that **114** (Scheme 40) can self-assemble into ion channels in the planar (*E*) conformation due to π–π stacking, but not in the twisted (*Z*) conformation.^535^

**Scheme 40:**
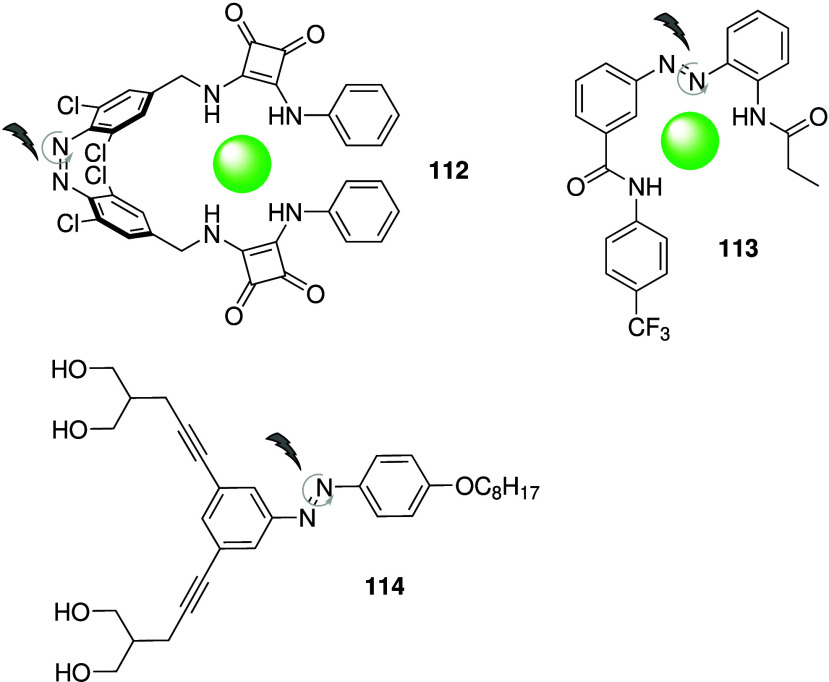
Structures of Azobenzene-Based Photoswitchable Anion Transporters **112**–**114**; All Structures Are Shown in Their Active Conformation with the Green Sphere Highlighting the Chloride Binding Mode

Other molecular photoswitches have also been employed for anion transport. Wezenberg and collaborators have reported stiff-stilbene-based anionophores appended with (thio)­ureas (e.g., **115**, Scheme 41)^223^ or amidopyrroles (e.g., **116**, Scheme 41).^536^ Bis-thiourea **115** was shown to bind chloride more strongly in the (*Z*) conformation than the (*E*) conformation, and as a result is also a better anion transporter in the (*Z*) conformation. Furthermore, (*Z*)-**115** was found to be a potent electrogenic Cl^–^ transporter with excellent Cl^–^ > OH^–^ selectivity and as a result was able to depolarize membranes and create a chloride gradient in liposomes. In contrast, amidopyrrole compound **116** did not show any difference in chloride binding ability between the (*E*) and (*Z*) isomers, but still showed better transport ability in the (*Z*) conformation. Probably this is the result of the more compact shape of the (*Z*) isomer (allowing more facile diffusion in the membrane) and the better anion encapsulation in the (*Z*) isomer. Talukdar and co-workers have reported a number of photoswitchable ionophores based on hydrazone photoisomerization (e.g., **117**–**119**, Scheme 41). Pyridine-containing phenylhydrazone **117** and acylhydrazone **118** were shown to preferentially transport anions in the open (*E*) conformation via electrogenic antiport mechanisms.^219,537^ Irradiation to convert the compounds to the (*Z*) conformation resulted in a decrease in transport activity due to an intramolecular hydrogen bond between pyridine N and the NH hydrogen bond donor which prevents anion binding. As a result, reversible switching could be achieved by irradiation with UV light to facilitate (*E*)-to-(*Z*) isomerization, followed by treating with acid to break the intramolecular hydrogen bond and destabilize the (*Z*) isomer to convert back to the (*E*) isomer. The behavior of thiophene-acylhydrazone **119** was slightly different.^538^ In the (*E*) conformation, **119** is able to facilitate the transport of cations and anions in a Na^+^/Cl^–^ symport mechanism. Presumably, the cation binds to the sulfur atom and acyl group, while the anion can bind to the NH and CH hydrogen bond donors on the other side of the molecule. Upon photoisomerization to the (*Z*) isomer, both cation and anion binding sites are disrupted and Na^+^/Cl^–^ symport is attenuated.

**Scheme 41:**
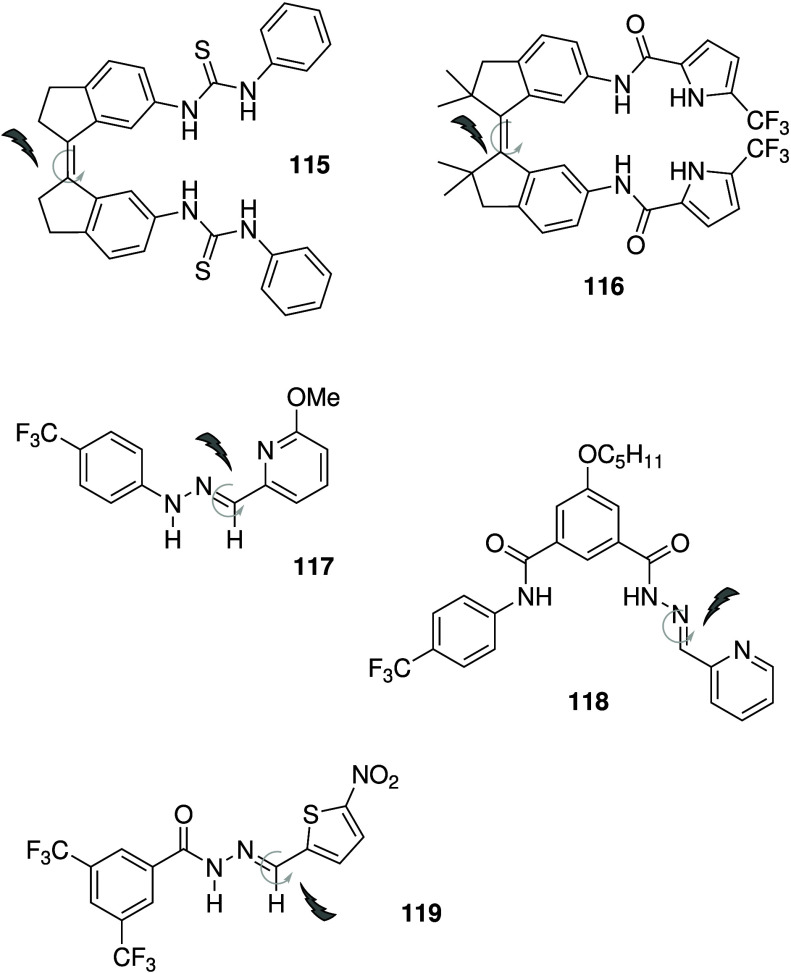
Structures of photoswitchable Ion Transporters **115**–**119**; All Structures Are Shown in Their Most Active Conformation

In addition to molecular photoswitches that function through light-activated conformational changes, light-dependent anion transport can also be achieved using photocaging techniques. In this method, photocleavable groups are attached to the transporters, which can be removed upon irradiation. Because this method involves breaking covalent bonds, it does not represent a reversible switch (where the activity of the transporter can be switch ON/OFF multiple times) but instead is a one-time activation method. It might therefore be more useful for anticancer or drug delivery applications than artificial cells, but there is still potential. A few examples of the photocaging method were already discussed in the anticancer section (see Scheme 18). These examples all relied on blocking the anion binding site of a mobile carrier with a photocleavable linker, but there have been some recent reports on different photocaging strategies. Talukdar and co-workers reported the properties of photocaged transporter **120** (Scheme 42).^539^ It was shown that the photocleavable bulky *ortho*-nitrobenzyl groups distort the planarity of the molecule and prevent it from π-π stacking. However, upon irradiation for 10 min with 365 nm light the bulky groups are removed, and the compound can self-assemble into an anion-selective ion channel. Another unusual mechanism of photocaging was reported by Bickerton and Langton.^540^ They appended a long lipophilic anchor to two different mobile carriers (**121** and **122**, Scheme 42). The lipophilic tail is attached far away from the anion binding site and does not influence anion binding ability. However, it strongly anchors the molecules to the membrane and diminishes their ability to diffuse within the membrane, thereby switching OFF the anion transport activity. Photoirradiation with a 365 nm LED cleaves the anchor and releases the mobile carrier, leading to a strong enhancement of transmembrane anion transport in POPC liposomes.

**Scheme 42:**
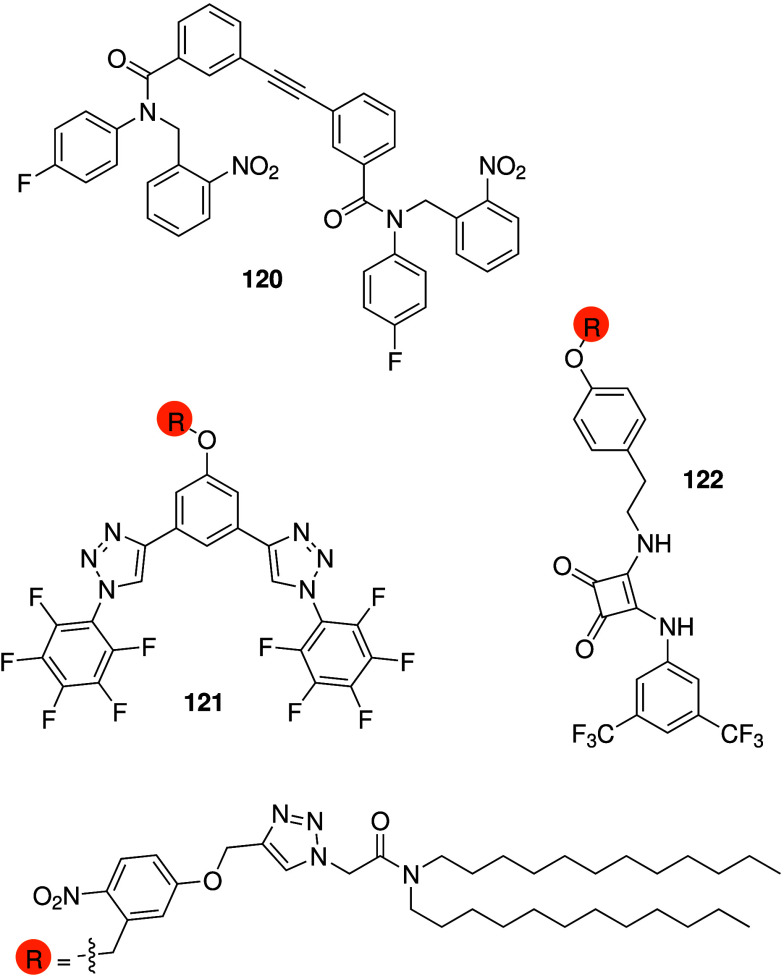
Structures of Photo-Caged Anion Transporters **120**–**122**; All Structures Are Shown in Their Caged Form

#### 3.2.3 Voltage-Dependent Synthetic Anion Transporters

Voltage-gated chloride channels are one of the main classes of biological chloride transporting proteins.^541^ For example, CLC-1 is the main chloride channel in skeletal muscle and is a voltage-gated channel that is deactivated at negative voltage. This is particularly interesting because in skeletal muscle the resting conductance is dominated by Cl^–^ and not K^+^ like other tissues, and mutations in CLC-1 are the cause of *myotonia congenita*. The more widely expressed CLC-2 channel is also a chloride channel activated by hyperpolarization. It is therefore no surprise that supramolecular chemists have tried to mimic this voltage-gated chloride transport behavior. While voltage-dependent studies on proteins are usually performed in polarized planar lipid bilayers, in order to test voltage-dependence in unilamellar vesicles the most common method appears to be the use of a K^+^ concentration gradient combined with valinomycin (an electrogenic K^+^ transporter) to generate the desired membrane potential (i.e., voltage).^542^ The resulting chloride transport can then be measured by common assays such as the HPTS assay.

Matile and co-workers were the first to report voltage-dependent chloride transport by synthetic systems. They reported a rigid-rod β-barrel forming synthetic chloride channel appended with an electron-donating group on one end and an electron-withdrawing group at the other end (push–pull system) (**123**, Scheme 43).^543,544^ As a result, the compound possesses a large axial dipole. It was shown that **123** shows enhanced partitioning, and thus improved chloride transport, in liposome membranes with inside-negative potentials. It was postulated that this is due to dipole-potential interactions that align molecules of **123** in a parallel fashion to form chloride-transporting tetrameric parallel β-barrels (despite dipole–dipole repulsion within the tetramer). In a later paper, they reported urea/amide macrocycles that also possess a strong molecular dipole and therefore also displayed strong voltage-dependent anion transport (e.g., **124**, Scheme 43).^19^ In this case, they reasoned that the macrocycles stack in a parallel membrane-spanning tetrameric nanotube through dipole–dipole interactions, and this stack is stabilized by increasing membrane potentials. Because anions are too large to go through the center of the nanotubes, Matile and co-workers suggested a Jacobs-ladder type mechanism to explain the voltage-dependent anion transport activity.

**Scheme 43:**
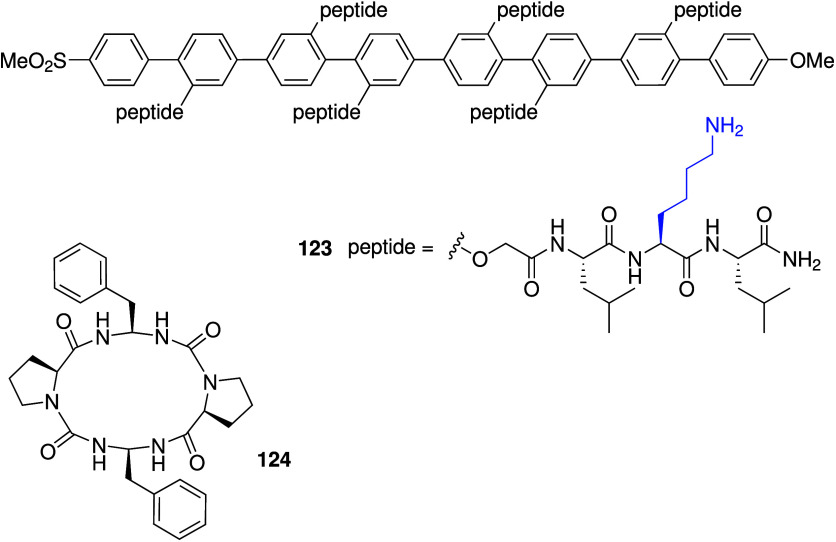
Structures of Voltage-Dependent Anion Transporters **123**–**124** Reported by Matile and Co-workers

Gale and co-workers recently reported a tetra-urea macrocycle with voltage-dependent behavior via a very different mechanism (**125**, Scheme 44).^40^ They found that tetra-urea **125** functions as a pure H^+^/Cl^–^ transporter in POPC liposomes, with no observable electrogenic uniport Cl^–^ activity. They reasoned that **125** binds very strongly to the phosphate headgroup of POPC lipids and therefore cannot diffuse throughout the membrane in its neutral form. Instead, only deprotonated **125** or the **125**·Cl^–^ complex can diffuse through the membrane and move from one interface to the other. This results in the observed H^+^/Cl^–^ transport, but also implies that transport only occurs through the movement of negatively charged species. As a result, this movement is dependent on the membrane potential and the transport activity of **125** was found to be diminished by both positive and negative potentials.

**Scheme 44:**
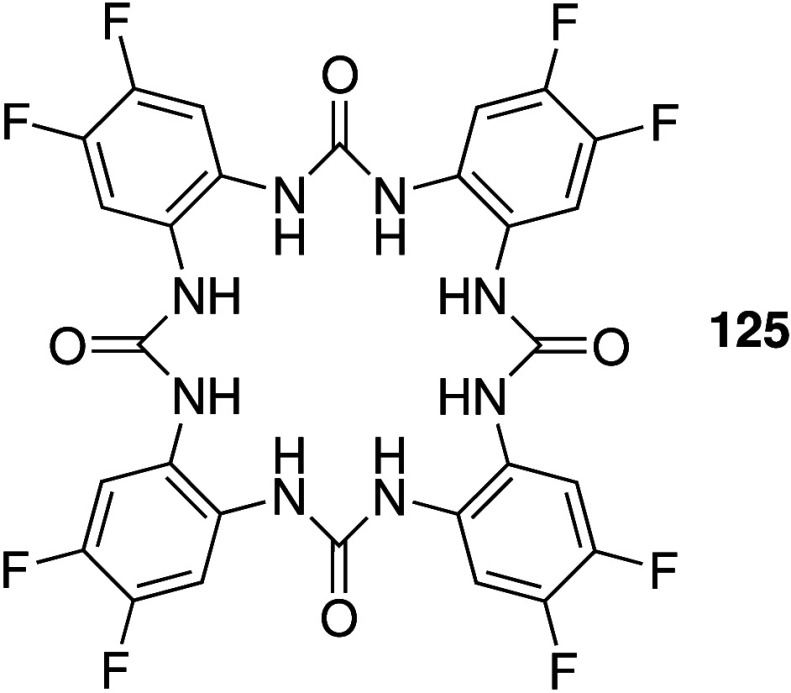
Structure of Voltage-Dependent Anion Transporter **125** Reported by Gale and Co-workers

#### 3.2.4 Ligand-Gated Synthetic Anion Transporters

Ligand-gating is another important regulatory system in biology whereby ion channels can be opened or closed in response to a chemical entity, such as a hormone or neurotransmitter. In terms of anion transport the best-known examples are probably the GABA_A_ receptors, which are ligand-gated chloride channel that are opened upon binding the neurotransmitter GABA.^545,546^ The GABA_A_ receptors play a crucial role in neuronal inhibition and are the target of many important classes of drugs, such as benzodiazepines and barbiturates. It thus comes as no surprise that achieving ligand-gated anion transport by synthetic anionophores has long been a target for biomimetic supramolecular chemistry. Again, the earliest examples came from Stefan Matile’s group. In their work on rigid-rod β-barrels appended with peptides (see Scheme 29 and Scheme 43), they had already shown that careful design of the peptide sequence allows blocking of the pore and inhibition of ion channel activity by various substrates (see also section 3.3). Furthermore, by appending cationic arginine residues to the outer surface the β-barrel channel activity could be inhibited due to insufficient membrane partitioning, but could be switched ON by adding an appropriate lipophilic counteranion.^547,548^ However, none of this work was performed on anion-selective rigid-rod β-barrels. To design an anion-selective pore with ligand-gating behavior, the Matile Group appended their octiphenyl rigid rods with naphthalenediimide (NDI) derivatives (**126**, Scheme 45).^549,550^ Self-assembly through π–π stacking of the NDI groups leads to a twisted, helical barrel that is essentially closed and shows no ion channel activity. The addition of dialkoxynaphthalene (DAN) derivatives allows intercalation of the electron-rich DAN molecules between the electron-poor NDI units through aromatic donor–acceptor interactions. This ‘ligand binding’ leads to untwisting of the barrel and the formation of a weakly anion-selective channel. Liposome and planar lipid bilayer studies, as well as CD experiments, confirmed the proposed ligand-gating behavior.

**Scheme 45:**
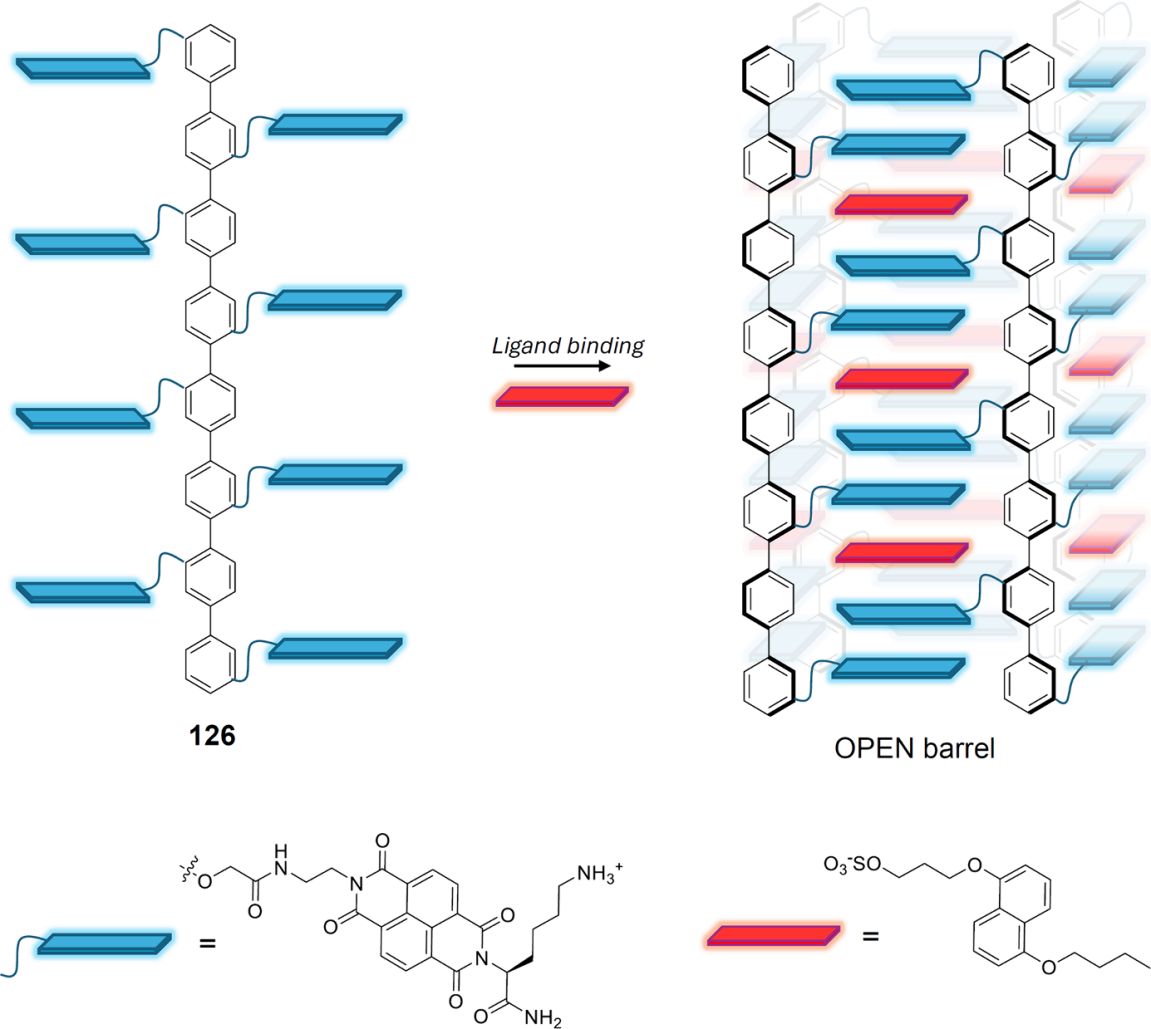
Structure of Ligand-Gated π-Barrel **126** Reported by Matile and Co-workers

Other synthetic anion channels with ligand-gating activity have also been reported. Nitschke, Keyser and co-workers showed that in the presence of Zn­(ClO_4_)_2_ ligand **127** can self-assemble into a pentagonal-prismatic Zn_10_L_15_ cage (Scheme 46a).^551^ It was shown that this cage can function as an anion-selective channel in liposomes and planar lipid bilayers and that transport activity can be switched OFF *in situ* by blocking the central channel with dodecyl sulfate. Other examples of ligand-gated transmembrane chloride transport have been reported by the Schmitzer group. For examples, imidazolium salt **128** was shown to function as an anion channel for chloride transport in liposomes, but this activity could be completely switched OFF by the addition of 2 equiv α-cyclodextrin through the formation of a very polar inclusion complex (Scheme 46b).^552^ In a separate paper, they also showed that compound **67** (Scheme 20), a synthetic anionophore with antibacterial activity (see section 2.4.1), could be switched OFF by the addition of β-cyclodextrin and could subsequently be switched back ON by the addition of a competitive guest such as 1-adamantanol.^273^ This last example represent the only report of a *reversible* ligand-gating strategy for transmembrane chloride transport, which is more relevant from a biomimetic perspective.

**Scheme 46:**
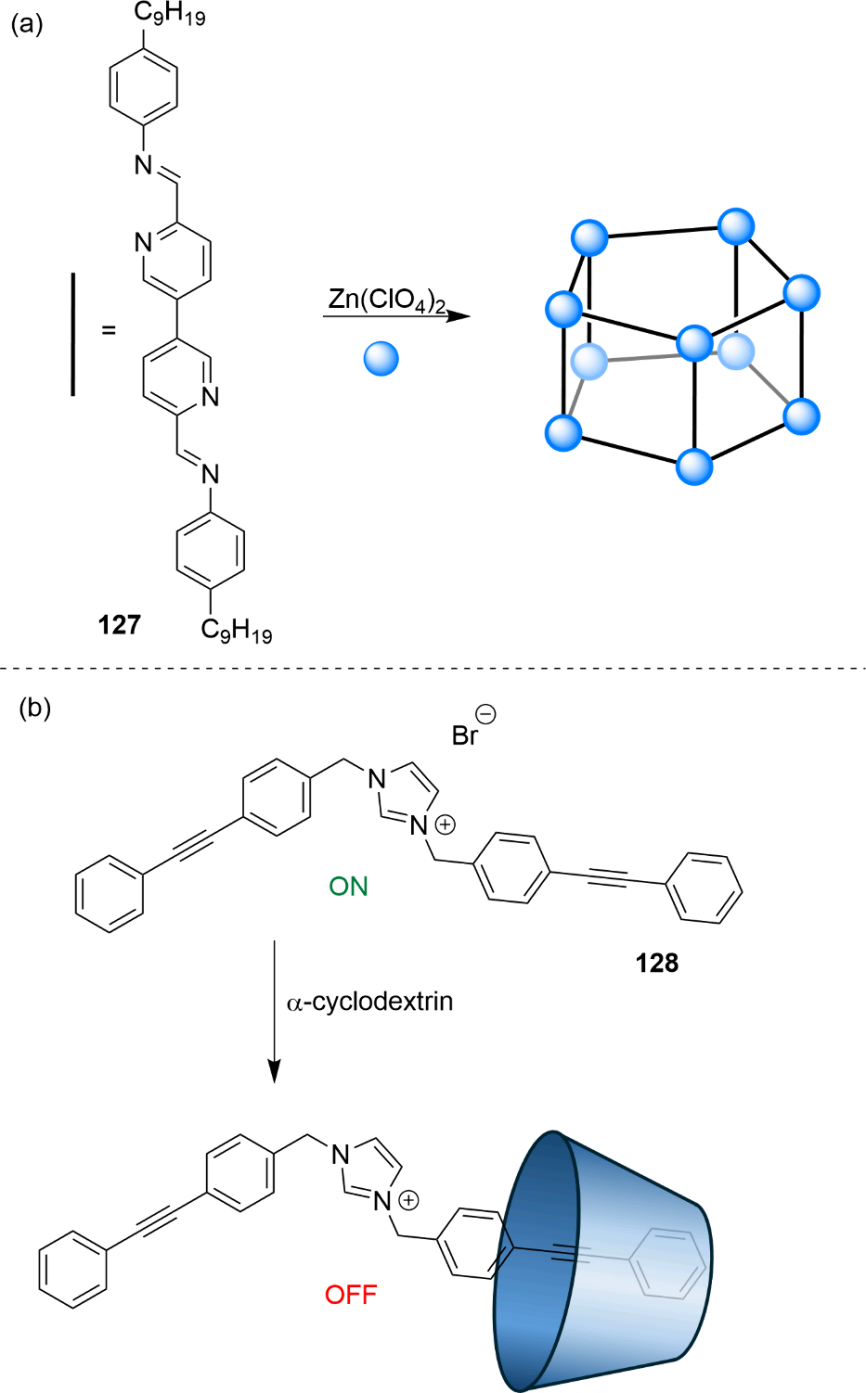
(a) Structure of Ligand **127** Which Can Self-Assemble into a Pentagonal Prismatic Ion Channel; (b) Structure of Imidazolium Salt **128** Which Functions As a Chloride Ion Channel and Can Be Switched OFF by the Addition of α-Cyclodextrin

#### 3.2.5 Redox Dependent Synthetic Anion Transporters

There have been many reports of redox-controlled anion transport by synthetic molecules. This has been largely driven by the fact that cancer cells contain high concentrations of GSH, a thiol-based reducing agent. Several examples of this approach were already mentioned in the anticancer section of this review (section 2.3). Other notable examples of this approach include the GSH-mediated sulfonium reduction reported by Manna (**129**) and Gabbaï (**130**) (Scheme 47). Manna and co-workers first reported that the tripodal thiourea-based pro-transporter **pro**-**129** has increased aqueous solubility compared to active transporter **129**, but also has negligible anion transport ability.^553^ However, incubation with GSH led to regeneration of **129** and restoration of anion transport ability. Furthermore, it was shown that both **129** and **pro-129** can transport chloride anions in human cell lines, but none of the compounds showed significant cytotoxicity. Similarly, Gabbaï and co-workers used sulfonium reduction to achieve redox-control of their stibonium-based anionophores.^554^ Dicationic **pro-130** was inactive as an electrogenic chloride transporter in liposomes, but could be switched ON *in situ* by the addition of GSH to the liposome solution.

**Scheme 47:**
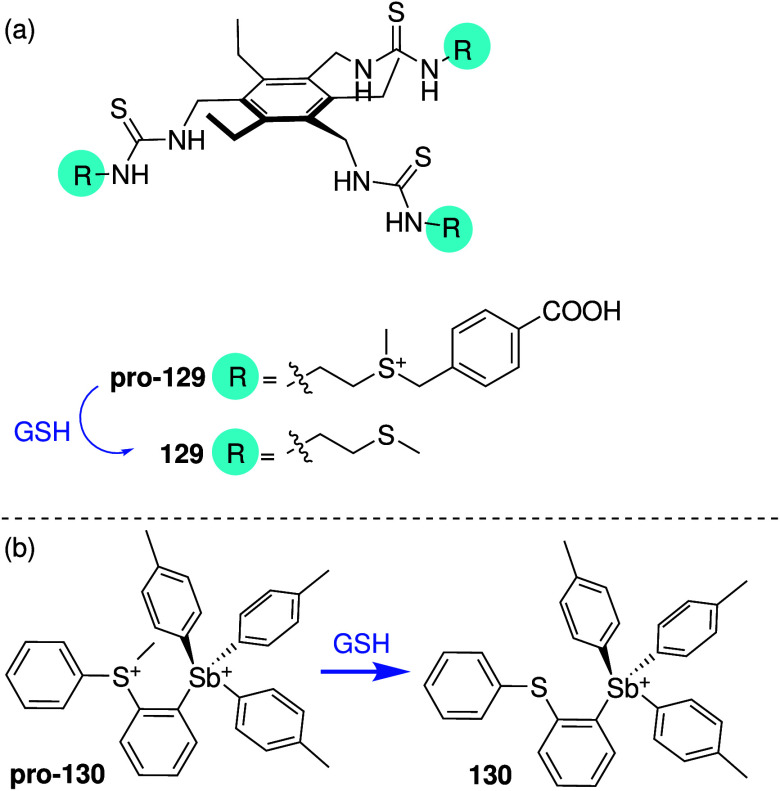
Redox-Controllable Anionophores (a) **129**, and (b) **130**

A different redox-cleavable mechanism has been recently proposed by Langton and co-workers.^555^ They caged an indole-based anion transporter with an azobenzene group that can be reductively removed (**131**, Scheme 48). It was shown that **131** was an active chloride transporter, but caged **pro**-**131** was not due to an unfavorable intramolecular hydrogen bond. However, treatment of **pro-131** with zinc and ammonium formate could remove the azobenzene group and free the active anionophore **131**. Because azobenzene cleavage under reductive environments has been previously explored as a prodrug strategy for activation in the hypoxic environment of solid tumors,^556^ it could provide another method for switchable anionophores with anticancer activity.

**Scheme 48:**
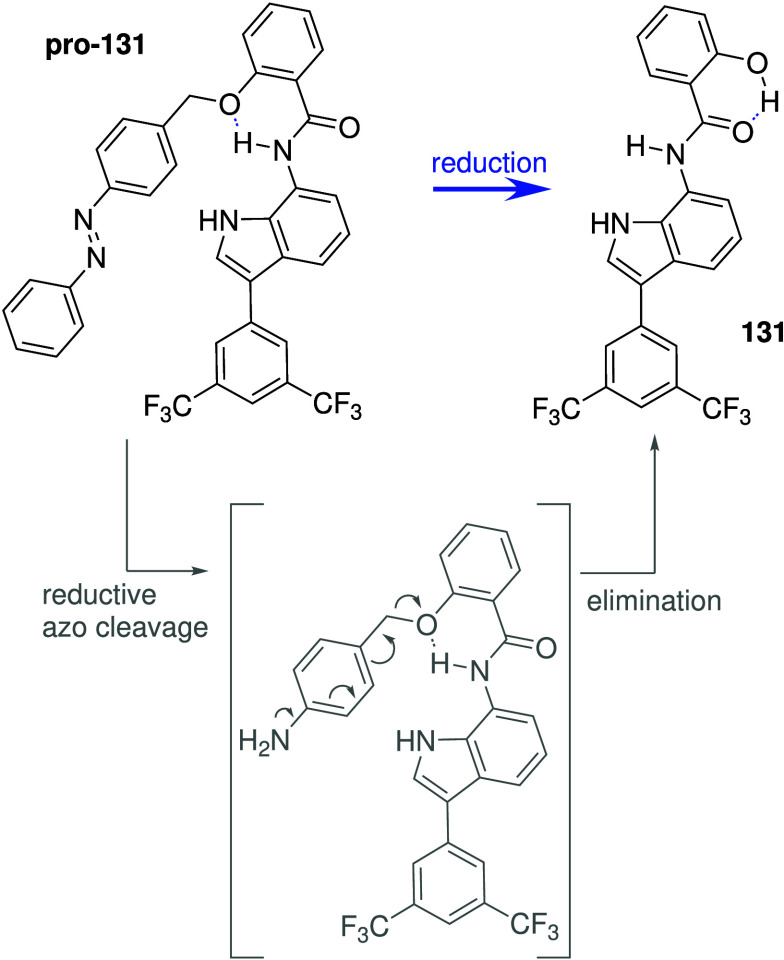
Structure of Redox-Switchable Transporter **131**

All redox-activatable anion transporters mentioned so far involve cleavage of a covalent bond and therefore can only be switched ON once. This is desirable for anticancer drugs, but less useful for other applications such as artificial cells. Langton and co-workers thus developed a chalcogen-bonding anionophore that is more reversible and can go through an OFF–ON–OFF sequence (**132**, Scheme 49).^557^ They showed that **132** is only able to function as an anion transporter in POPC liposomes in the Te­(IV) oxidation state, but not in the Te­(II) or Te­(VI) oxidation states. As a result, *in situ* switching experiments could be performed in liposomes whereby transport was initially switched OFF using **132**-**Te­(VI),** addition of 1 equiv DTT as reducing agent switched ON anion transport through the generation of **132-Te­(IV)**, and addition of further excess DTT switched transport back OFF through the generation of **132-Te­(II).**

**Scheme 49:**
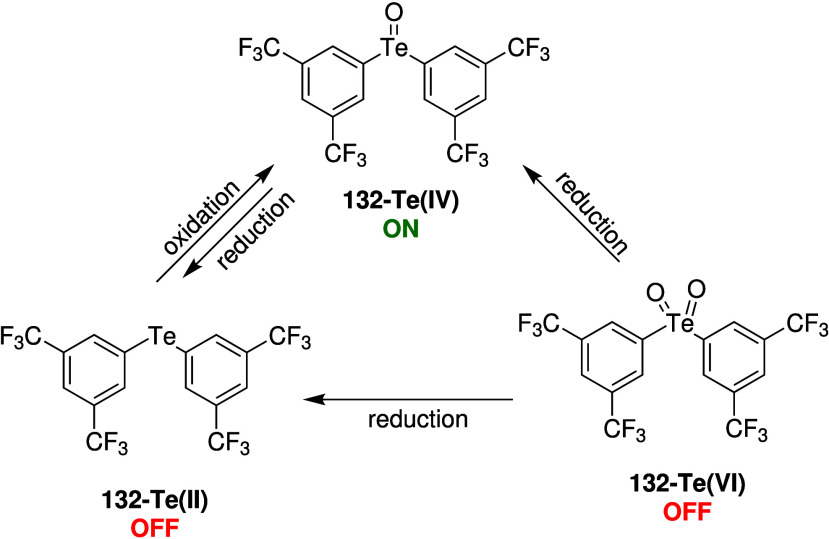
Structure of Redox-Switchable Transporter **132** in Its Various ON and OFF States

#### 3.2.6 Multistimuli Dependent Synthetic Anion Transporters

In recent years there have been a few reports of synthetic anion transporters that can be switched ON/OFF by multiple stimuli. Such multiple stimuli can not only provide better control of their transmembrane transport event and can better mimic biological transport proteins that have many control factors, they also serve as liposome-based logic gates (usually AND gates). For example, Chmielewski and co-workers appended an *ortho*-nitrobenzyl photocaging group to their previously reported pH-switchable anion transporter **105** (Scheme 38), to generate a pro-anionophore that can only be switched on by irradiation with UV light *and* at acidic pH.^558^ In another example, Manna and co-workers used a sulfonium-based tetra-thiourea containing a *para*-nitrobenzyl group for multistimuli reactive anion transport (**133**, Scheme 50).^221^ They reasoned that the active anionophore can be generated by removal of the nitrobenzyl group by either GSH (similar to compound **129**, Scheme 47) *or* by nitroreductase enzymes (which can reduce the nitro group, followed by a cascade reaction to remove the benzyl group). It was shown that the *para*-nitrobenzyl group can indeed be removed by GSH or by treatment with bacterial cell lysate (which contains nitroreductases), and that the generated compound was an active transmembrane chloride transporter.

**Scheme 50:**
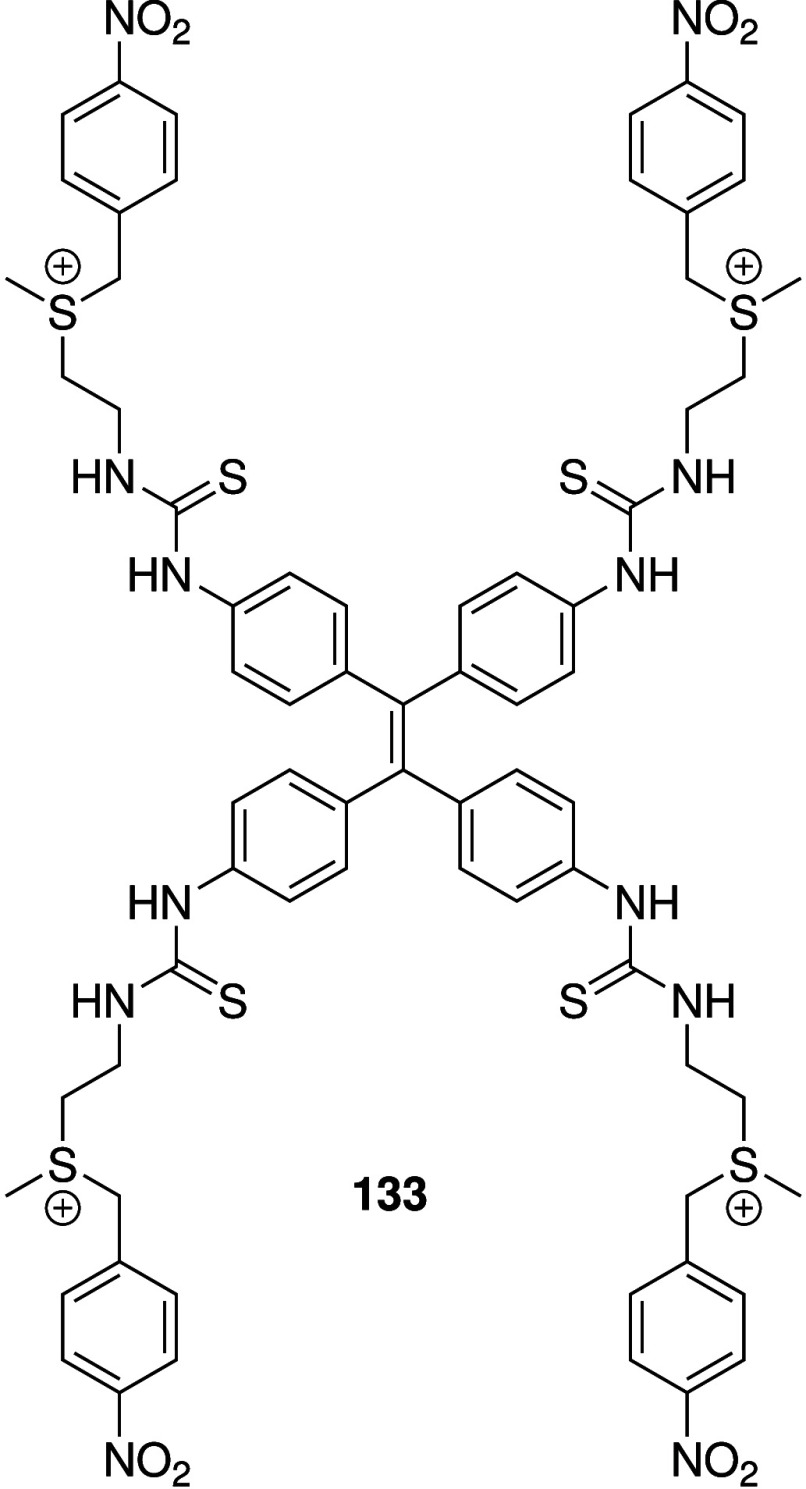
Structure of Pro-Transporter **133**, Which Can Be Switched on by GSH or Nitroreductases

Another example of a dual-stimuli synthetic anion transporter came from the group of Barboiu.^559^ They reported imidazole-based compounds (e.g., **134**, Scheme 51) which were shown to self-assemble into chloride selective anion channels in liposomes and planar lipid bilayers. At low pH activity was enhanced due to better H^+^/Cl^–^ symport, while at high pH transport was also enhanced due to improved OH^–^/Cl^–^ antiport (with little activity observed around neutral pH). Furthermore, at high pH **134** also showed voltage-responsive anion transport, suggesting a dual voltage/pH responsive mechanism for the synthetic anion channel. A very rational design for dual response anionophores was recently developed by Langton and co-workers.^560^ They appended an isophthalamide-based anion transporter with two different cleavable blockers: a light-cleavable *ortho*-nitrobenzyl group and a H_2_S-cleavable *para*-azidobenzyl group (**135**, Scheme 51). Whereas **135** itself was inactive as an anion transporter in liposomes, pretreatment of **135** with 405 nm light *and* NaSH (in any order) could generate an active transmembrane anion transporter. In contrast, irradiation with 405 nm light only, or incubation with NaSH only, failed to produce an active anion transporter, confirming the dual response AND logic gate for anion transport.

**Scheme 51:**
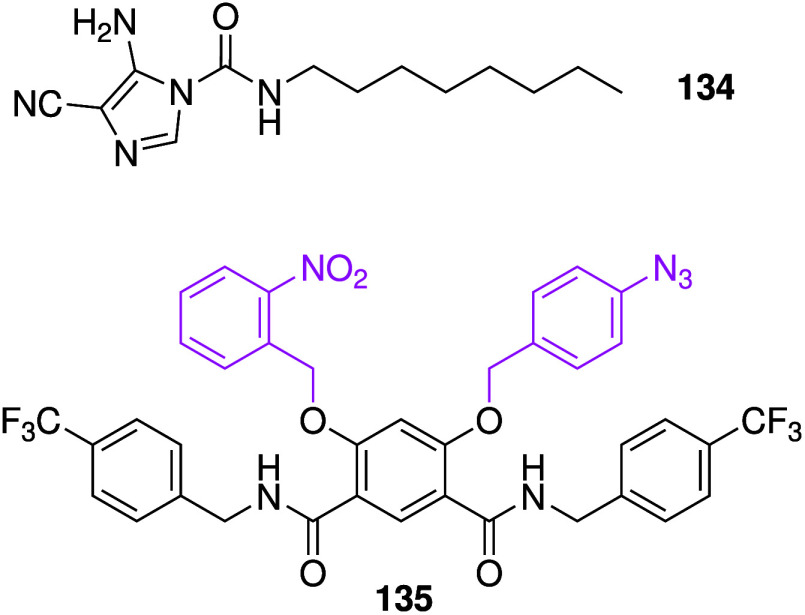
Dual-Response Transmembrane Anion Transporters **134**–**135**; Cleavable Linkers Are Highlighted in Magenta

#### 3.2.7 Active Transport by Synthetic Anion Transporters

The synthetic anionophores mentioned thus far have all only been shown to function as passive transporters, i.e. they allow the transmembrane transport of anions along their electrochemical gradient. However, in biological systems ion pumps can transport ions against their gradient and these proteins are therefore used to create or maintain transmembrane ion gradients and membrane potentials.^561^ The energy required for this active process can come from ATP hydrolysis (e.g., ATPase),^562^ light (e.g., halorhodopsins),^563^ or secondary active transport where transport of an ion against its concentration gradient is coupled to the transport of another ion along its electrochemical gradient (e.g., KCC transporters–which achieve active Cl^–^ transport by cotransporting K^+^ along its electrochemical gradient).^564^ Mimicking such energetic systems with simple synthetic systems is an arduous task. While Shao, Fu and Aprahamian recently reported a light-driven molecular anion pump, it was only tested for transport across bulk organic layers in a U-tube setup.^565^ True transmembrane active anion transport has only been reported twice by the Gale group. In a first report, they used fatty acids to fuel active transport of Cl^–^ in liposomes.^566^ Long fatty acids are known to generate pH gradients across phospholipid bilayers, and the addition of H^+^/Cl^–^ symporters (e.g., prodigiosin, squaramide **36**, or bis-urea **136**, Scheme 52) to liposomes treated with fatty acid thus leads to H^+^/Cl^–^ driven by the pH gradient generated by the fatty acids. This is essentially a type of secondary active transport and allows the generation of transmembrane chloride gradients in systems that were originally lacking a chloride gradient. Liposome studies using HPTS (a pH sensitive dye) or lucigenin (a chloride sensitive dye) showed that a chloride gradient of 35 mM could be generated by the stepwise addition (10 additions) of fatty acid to liposomes containing synthetic H^+^/Cl^–^ symporter **136**.

**Scheme 52:**
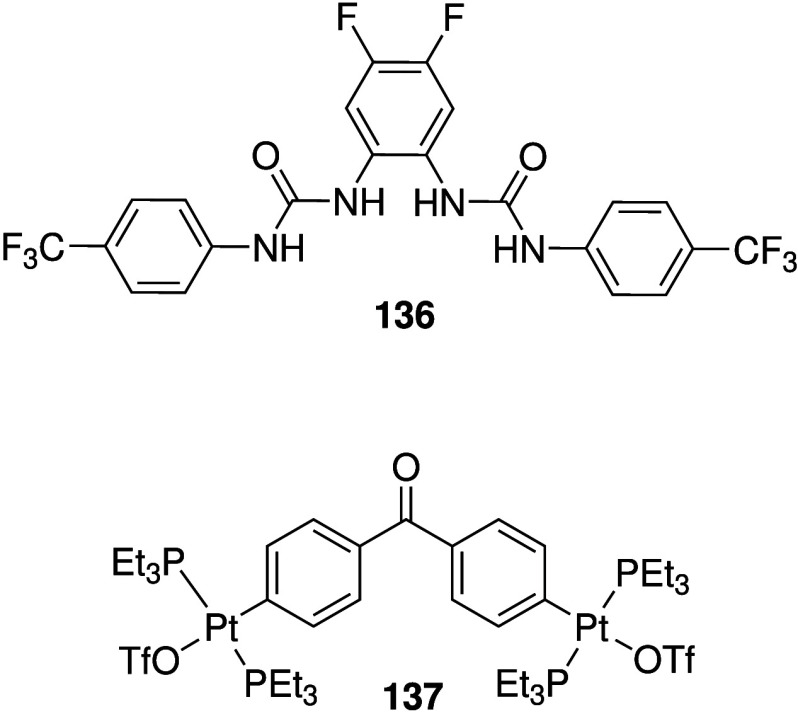
Synthetic Active Anion Transporters **136**–**137**

In the second report of Gale and co-workers, they used a different approach to achieve active transport of OH^–^ in liposomes.^567^ They noticed that Pt­(II) compounds such as **137** (Scheme 52) are able to generate substantial pH gradients in liposomes. After rigorous mechanistic studies the authors reasoned that the Pt­(II) complexes undergo solvolysis in the aqueous solution surrounding the liposomes to produce aqua complexes (by ligand exchange with the weakly bound ^–^OTf anions). Due to the p*K*_a_ of these aqua complexes (estimated around 9), they will exist as an equilibrium between cationic Pt–OH_2_^+^ and neutral Pt–OH complexes. The neutral complex, but not the cationic complex, will then be able to cross the membrane and reach the internal aqueous solution, where it is reprotonated to the cationic Pt–OH_2_^+^ complex and thereby generates OH^–^ in the internal solution. This increases the internal pH and it was demonstrated that a gradient ΔpH > 2 can be generated by 6 stepwise additions of **137** to POPC liposomes.

#### 3.2.8 The Future of Synthetic Anion Transporters in Artificial Cells

The sections above illustrate the progress that has been made regarding the development of synthetic anion transporters that can be regulated with external stimuli and thereby better mimic biological transport proteins. In terms of biomimicry, we could not find any reports of synthetic anionophores that can mimic the behavior of the biologically relevant Ca^2+^-activated Cl^–^ channels or volume-regulated anion channels. In addition, for any of switchable biomimetic anion transport systems mentioned in the previous sections to be considered an artificial cell, the anion transport event should be combined with an interesting ‘intracellular’ function (e.g., bringing metabolites in the cell for an enzymatic reaction, switch on transcription, ...). Recently, Hennig and Nau used synthetic cation transporters for this purpose,^568^ but coupling anion transporters to metabolic reactions inside a liposome has not yet been reported. We encourage researchers working on switchable anion transporters to reach out to collaborators working on artificial cells. In addition, it should be noted that artificial cells do not necessarily consist of phospholipid bilayers as the membrane. Other compartmentalization systems have also been used for artificial cell development, such as membranes made from polymers (polymersomes),^569^ peptides (peptosomes),^570^ proteins (proteinosomes),^571^ or colloidal particles (colloidosomes).^572^ Such abiotic systems often have greater stability compared to liposomes and could attribute novel properties. It would therefore be interesting to see if any of the anionophores covered in this review can also transport anion across these more unusual membranes. As far as we know, there has been only one report of the use of a synthetic anionophore in a nonphospholipid vesicle. Stefan Matile demonstrated that their “dynamic amphiphiles” (similar to compound **88**, Scheme 32) are able to transport DNA into polymersomes, including polymersomes embedded in an agar gel.^573^ There is thus plenty of opportunity for the field of synthetic anion transporters to move toward more advanced biomimicry and artificial cells.

### 3.3 Sensors

Sensing, i.e., the development of methods to detect the presence and/or quantity of a particular analyte, is one of the largest endeavors in supramolecular chemistry.^574^ We believe that there is great potential in the use of artificial transmembrane anion transporter to augment the development of anion sensors. To understand why, it is instructive to look at the field of biosensors. A biosensor is an analytical device that combines a biological recognition element such as an enzyme, antibody, or nucleic acid with a signal transducer to detect and quantify specific analytes.^575,576^ One of the most commonly used biosensor set-ups is the use of transport proteins or DNA nanopores embedded in a planar lipid bilayer (or sometimes a polymer membrane).^577^ By electrically separating two aqueous solutions, these membrane embedded pores create a controlled environment where analyte transport is driven by an electrical potential. When an analyte in the aqueous solution passes through the nanopore, it will temporarily interfere with the ionic current through the pore (Figure 2). The blocking current level, dwell time and translocation frequency provide information about the nature of the analyte and allows single molecule detection.^578,579^ One of the most commonly used nanopores in these biosensors is α-hemolysin, a bacterial exotoxin that can form heptameric transmembrane pores with an inner diameter of 1.4 nm at its narrowest point.^580^ The pore is large enough for a variety of analytes to pass through and this protein (as well as some engineered versions thereof) have been used for the stochastic sensing of a variety of analytes, ranging from inorganic ions and small organic molecules,^581–583^ to peptides and DNA.^584,585^ One of the most spectacular uses of α-hemolysin and other biological nanopores as biosensors has been its incorporation into DNA sequencing technology.^586–589^ This application is based on the fact that the pore of α-hemolysin is large enough to allow the passage of single-stranded DNA (ssDNA) but not double-stranded DNA (dsDNA). The size differences among the four nucleobases produces different specific blocking currents allowing discrimination between different nucleobases, and thus sequencing of a single DNA strand. Since its inception, nanopore-based sensors have become a large field that uses a variety of natural and engineered proteins and DNA nanopores combined with various membranes as a means to detect the presence of a plethora of analytes. As this review focuses on synthetic transporters, we will not discuss these biosensors in detail. They are merely mentioned here to illustrate the power of transmembrane transport in sensor development. Readers are referred to reviews on nanopore-based sensors for more information on the subject.^590–596^

**Figure 2:**
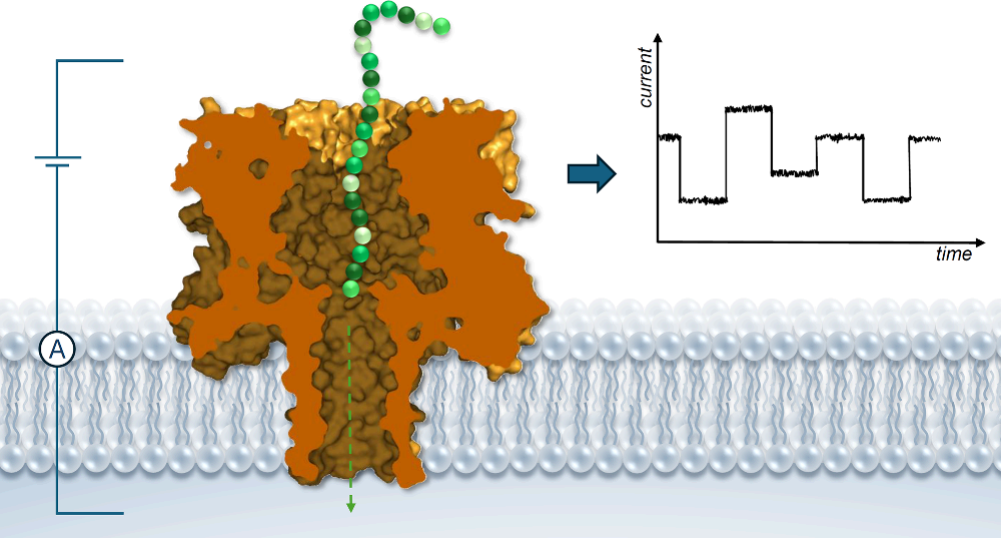
Principle of nanopore-based biosensors: a pore-forming protein is embedded in a membrane and a voltage is applied across the membrane, allowing electrical current to flow through the pore. When an analyte moves through the pore, the current will be affected due to blocking of the pore. This blocking can be measured by monitoring the current over time.

Inspired by the success of natural pore-forming proteins such as α-hemolysin in biosensors, Matile and co-workers tried to use their synthetic pores in a similar fashion to create new sensing systems.^597–599^ Matile’s original synthetic pores are artificial rigid rod β-barrels formed through the self-assembly of *p*-octiphenyl groups linked to short peptide chains (for an example, see Scheme 29). Simple modifications of the amino acids in the peptide chains can vary the chemical and physical properties of both inner and outer pore surface and the barrels can thus be tailored toward a specific sensing application.^597,600,601^ Whereas biosensors typically use current measurements for sensing (see Figure 2), the Matile group introduced a simpler optical method for their transporter-based sensing systems. They designed a variation of the carboxyfluorescein (CF) leakage assay to turn their β-barrels into sensing systems. The fluorescent dye CF is encapsulated into EYPC liposomes at concentrations where it self-quenches. Normally, when the synthetic β-barrels are added to the system CF moves through the barrel and an increase in fluorescence intensity is observed due to dilution of the dye. However, if an analyte can bind to the inside of the pore, CF leakage is blocked and no change in fluorescence is observed. This principle can be applied to monitor enzymatic reactions: if the substrate of the enzymatic reaction binds more effectively to the pore than the product, enzymatic activity gradually removes the blocking agent, allowing the pore to open. Conversely, if products exhibit stronger binding than substrates, the enzymatic reaction produces blocking agents, gradually closing the pore.^597,602–604^ By careful experimental design, the Matile group has also shown that this method can be used to sense specific analytes. In one of their first proof-of-concept examples they developed this method for sugar sensing.^605^ They used an octiphenyl β-barrel with a peptide sequence that places hydrophobic leucine residues on the outside of the barrel, and a histidine-lysine cationic dyad on the inner surface of the barrel. It was shown that this β-barrel could be efficiently blocked by ATP but not ADP. To turn this system into a sensor for sucrose, they employed enzymatic reactions using invertase and hexokinase as cosensors (Figure 3). Incubation of soft drinks with invertase produces fructose and glucose from the sucrose present in the drink. Phosphorylation of these monosaccharides by hexokinase yields glucose-6-phosphate and fructose 6-phosphate, while simultaneously converting ATP (good pore blocker) into ADP (poor pore blocker). The resulting difference in pore blockage efficiency between ATP and ADP leads to CF leakage and allows the determination of the sucrose concentration in various soft drinks (Coca-Cola, Coca-Cola Light, Red Bull, Fanta Orange, and Nestea Lemon). The same cationic β-barrel has also been used by the Matile group for the detection of inositol phosphates (IP_*n*_’s = IP_6_ and IP_7_) in almonds, lentils and soybeans.^606,607^ Note that in each case the selectivity of the sensing system for a particular analyte is mostly regulated by the nature of the enzyme that is added to the system.

**Figure 3:**
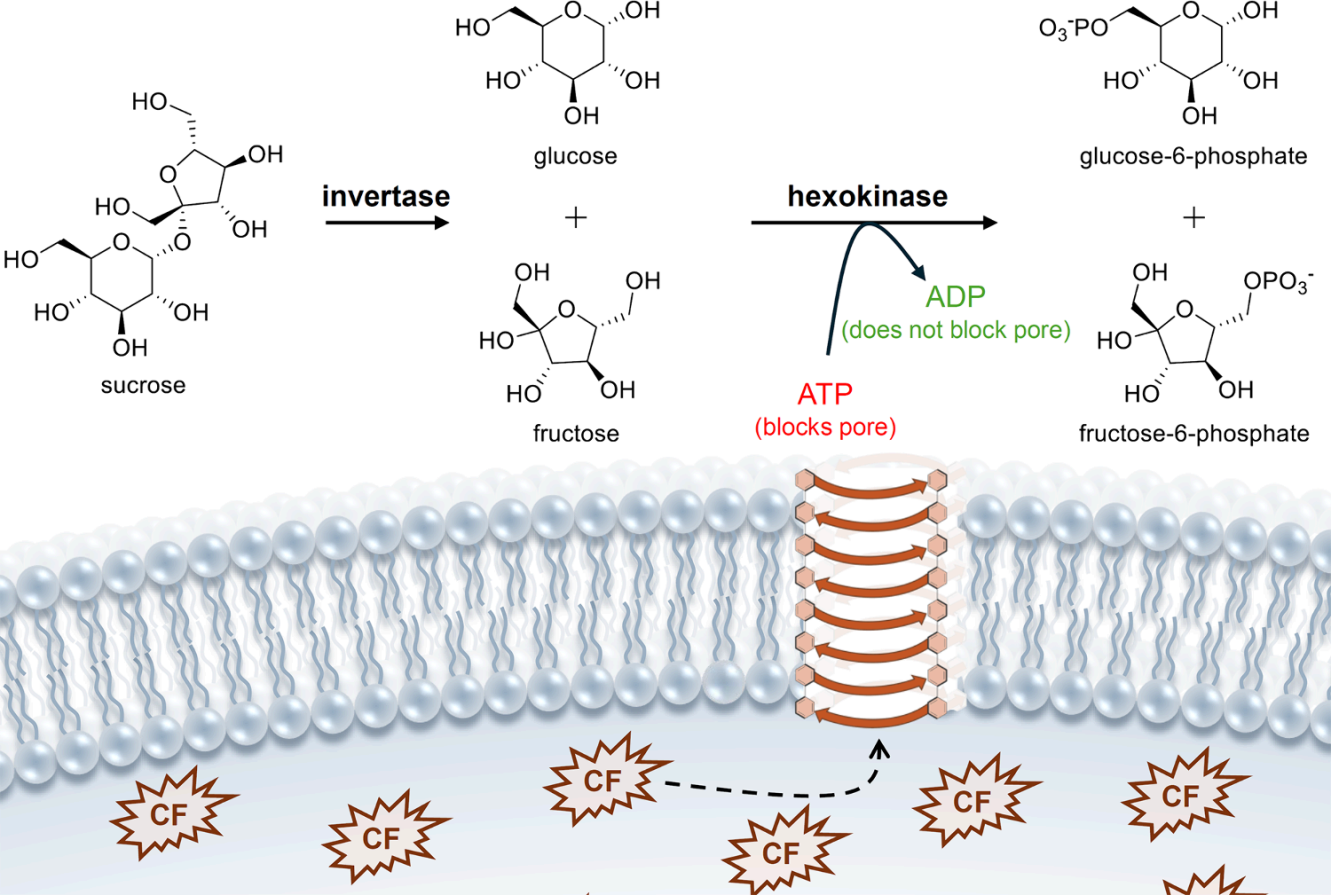
Principle of Stefan Matile’s β-barrel pore-based sucrose sensing system. Enzymatic conversion of sucrose to glucose-6-phosphate and fructose-6-phosphate leads to the conversion of ATP to ADP. As a result, the synthetic β-barrel pore becomes unblocked (“open”) and carboxyfluorescein (CF) can leak out, leading to an increase in fluorescence. Adapted from ref 605. Copyright 2005 American Chemical Society.

The Matile group continued their work in this area by introducing signal amplifiers to improve the sensing sensitivity of analytes that are bad pore blockers. The signal amplifiers react *in situ* with the products of the enzymatic reaction and thereby generate a better pore blocker. This principle was first applied to detect lactate in sour milk (Figure 4).^608^ Lactate oxidase served as a specific signal generator converting lactate into pyruvate. Because pyruvate is a poor blocker of the lysine-histidine β-barrel pore, cascade blue hydrazide was introduced as the signal amplifier. This hydrazide selectively reacts with pyruvate to make a cascade blue pyruvate hydrazone, which acts as a potent pore blocker. As a result, addition of sour milk pretreated with lactate oxidase and cascade blue hydrazide to carboxyfluorescein-loaded EYPC liposomes containing the synthetic β-barrel allows the determination of the lactate concentration in the sour milk. Optimization of the enzymes, amplifiers and synthetic β-barrels further allows this strategy to be used for the detection of other analytes such as lactose, citrate, acetate, α-ketoglutarate and glutamate.^608–611^ Moreover, the concept even expands to differentiate two enantiomers such as l- and d-lactate.^612^

**Figure 4:**
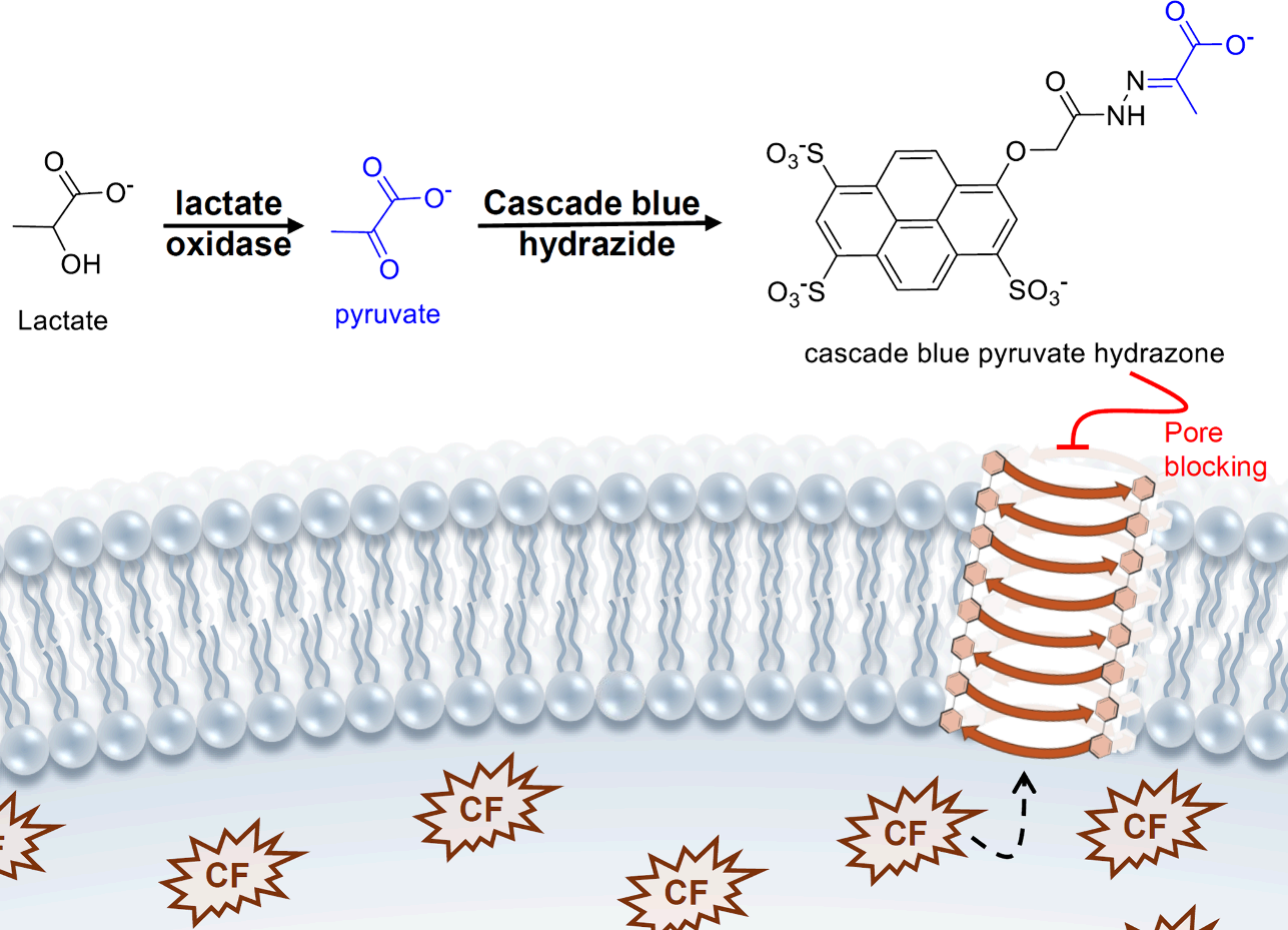
Principle of Stefan Matile’s β-barrel pore-based lactate sensing system. Lactate (e.g., in sour milk) is converted to pyruvate by the enzyme lactate oxidase. Pyruvate then reacts with signal amplifier cascade blue hydrazide to generate cascade blue pyruvate hydrazine, which effectively blocks synthetic β-barrel pores and blocks the leakage of carboxyfluorescein (CF). Different intensities of CF fluorescence will therefore be observed in the presence of different concentrations of lactate.

Instead of using their synthetic rigid-rod β-barrels, the Matile group has also introduced counterion activators as a novel approach for transmembrane transport-based sensors.^613^ This was based on their observation that amphiphilic anions can facilitate the uptake of cationic CPPs such as polyarginines via counterion exchange, and that amphiphilic cations can mediate DNA delivery (see drug delivery section 2.5). It was observed that a complex between oligoarginine and amphiphilic cations (the counterion activators) can transport carboxyfluorescein (CF) out of liposomes due to partial anion exchange between the original counterion activator and anionic CF. With this in mind, Matile and co-workers developed a sensing system for cholesterol that is reminiscent of the signal amplifier method with β-barrels.^614^ Cholesterol was pretreated with cholesterol oxidase (and Triton X-100 for solubility) to generate a hydrophobic ketone that can react with cascade blue hydrazide to form an amphiphilic anion (Figure 5). This amphiphilic anion can function as a counterion activator, and the addition of polyarginine and liposomes loaded with self-quenching CF results in the transport of CF out of the liposomes, leading to an increase in fluorescence intensity. Using this method, they were able to determine the cholesterol content in egg yolks, black lumpfish eggs and human blood serum. A similar polyarginine-counterion transport system was also used to develop a screening assay for inhibitors of the hyaluronidase enzyme.^613^ In subsequent manuscripts, Matile and co-workers have been able to obtain similar results with guanidium containing synthetic polymers based on polyguanidino-oxanobornenes (PGONs).^615^ These PGONs were also shown to transport CF out of liposomes and this ability could be enhanced by the addition of amphiphilic anions (e.g., pyrene butyrate) or diminished by very hydrophilic anions (e.g., ATP, phytate, heparin and cascade blue). As a result, the PGONs could be used to develop a sensor for lactate, by treating sour milk with lactate oxidase followed by cascade blue hydrazide and then PGONs and CF-loaded liposomes.

**Figure 5:**
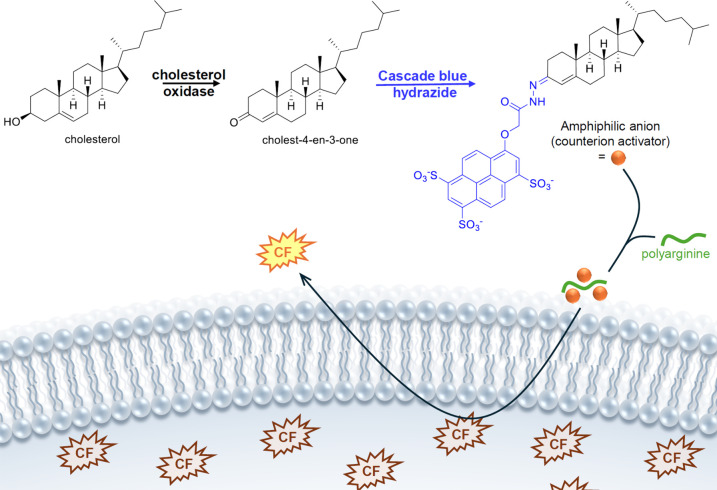
Principle of Stefan Matile’s counterion activator-based cholesterol sensor. Cholesterol is treated with cholesterol oxidase to generate cholest-4-en-3-one, which can react with cascade blue hydrazide to form an amphiphilic anion. The amphiphilic anion can function as a counterion activator for polyarginine, where the polyarginine–counterion complex is able to transport CF out of liposomes via counterion exchange.

In a similar fashion, it was shown that amphiphilic cations can function as counterion activators for DNA. Thus, the complex of calf thymus DNA (ctDNA) with an amphiphilic cation such as calixarene **138** can transport cationic molecules across bulk liquid membranes and liposomes via counterion exchange. To turn this property into a sensing system, the Matile group used a modified HPTS-DPX leakage assay. HPTS is an anionic fluorophore that is quenched by the cationic quencher DPX. When the cationic DPX quencher is transported out of the liposomes by the DNA-countercation complex, HPTS fluorescence will increase.^616,617^ It was illustrated that this property can be used for phytate sensing (Figure 6).^617^ Phytate is the fully phosphorylated version of inositol and bears a high negative charge. As a result, phytate can compete with DNA for binding to calixarene **138** and therefore no leakage of DPX is observed in the presence of phytate. However, when phytate is fully hydrolyzed to neutral inositol using the enzyme phytase, the **138**-ctDNA complex can function as a transporter for DPX and fluorescence increases. By comparing the fluorescence of the system before and after the treatment of almond extract with phytase, Matile and co-workers were able to determine the phytate content in almonds. In addition, when the ATP aptamer is used instead of calf thymus DNA the system could be converted to an ATP sensor.^618^ In a later manuscript, they showed how this DNA counterion activation method can also be used for differential sensing (also called “artificial nose” or “multisensor array”).^619^ This was achieved by *in situ* generating the amphiphilic counterion activators from the reaction of cationic hydrazides with lipophilic aldehydes and ketones found in various fragrances, followed by the addition of ctDNA and HPTS/DPX-loaded liposomes. By using multiple cationic hydrazides combined with principal component analysis or hierarchical clustering, the method was able to differentiate at least 21 analytes and could also differentiate between different brands of commercial perfumes.

**Figure 6:**
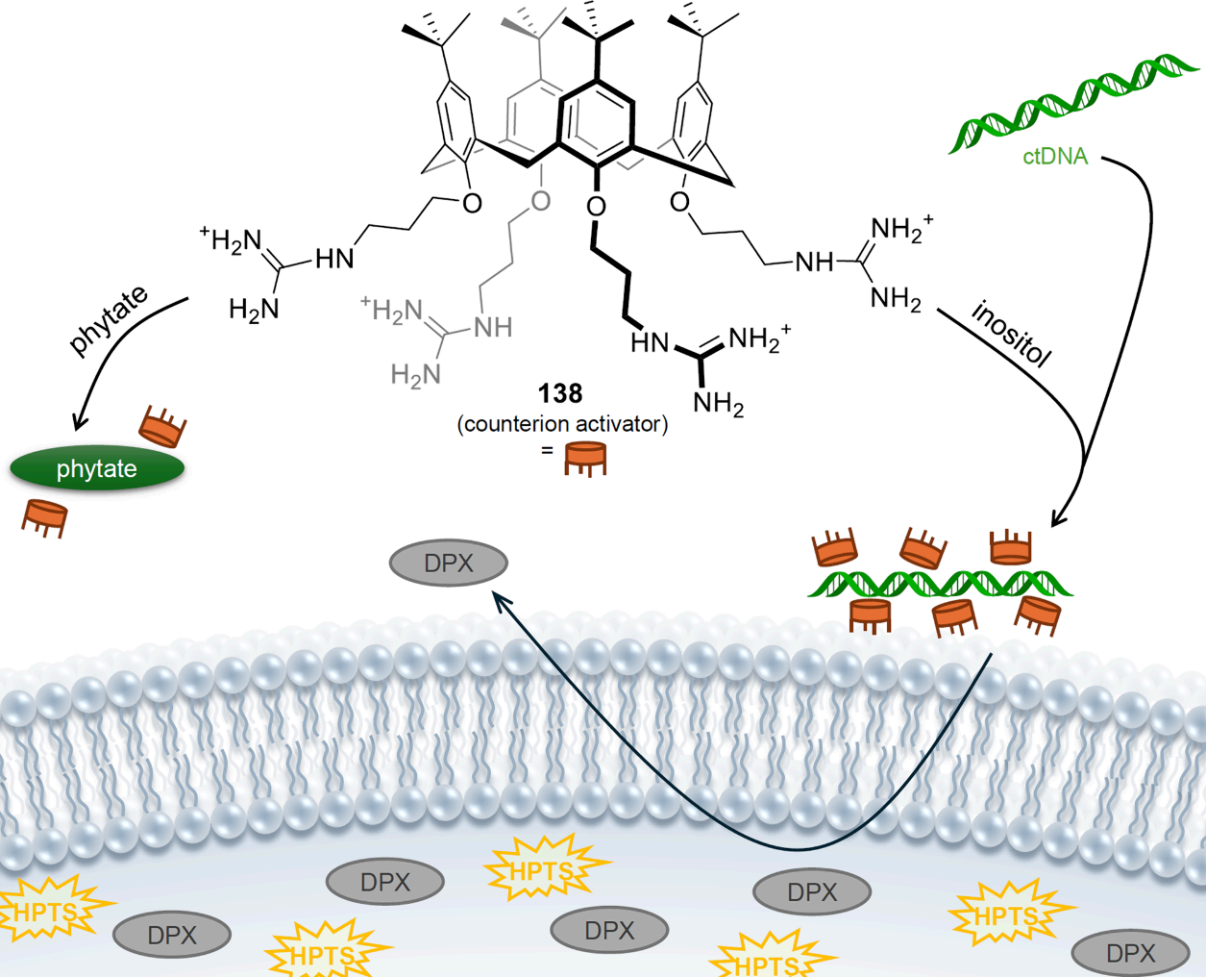
Principle of Stefan Matile’s counterion activator-based phytate sensor. Calixarene **138** functions as a counterion activator for ctDNA, allowing the transport of DPX out of the liposomes, leading to an increase in HPTS fluorescence. In the presence of phytate, phytate competes with ctDNA for binding to calixarene **138** and DPX is not transported. When phytate is hydrolyzed to inositol by phytase, DPX transport is restored.

The Busschaert group used a very different approach for employing transmembrane anion transport for sensing applications.^620^ They used transmembrane anion transport as an additional orthogonal selectivity filter to increase the selectivity of water-soluble anion sensors. The hypothesis is that by encapsulating a fluorescent anion sensor into liposomes containing an anion transporter, only those anions that are both transported across the membrane and induce an optical change of the encapsulated fluorophore will be sensed by the system (Figure 7). Essentially, this method employs the barrier function of membranes to keep out potentially interfering analytes. Note that neither the fluorophore nor the transporter need to be highly selective, they only need orthogonal selectivity. Using this method, they were able to show that lucigenin could be converted into a selective sensing system for iodide, capable of accurately estimating [I^–^] concentrations even in the presence of a large excess of NaCl. Only a simple anionophore such as *N,N*-diphenylurea was required to obtain selectivity. Recently, the same group showed that a similar technique can be employed to increase the selectivity of optical anion sensors that are not water-soluble, by using liquid–liquid extraction instead of transmembrane transport.^621^

**Figure 7:**
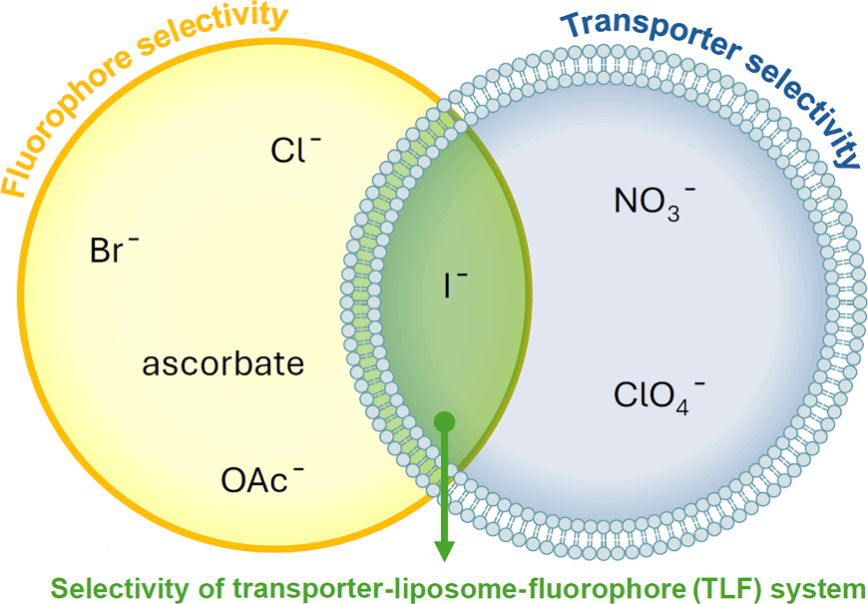
Principle of the transporter–liposome–fluorophore (TLF) system for anion selectivity: a partially selective fluorophore is encapsulated inside liposomes containing a synthetic anion transporter. Only those anions that are both transported and induce an optical change in the fluorophore will be sensed by the system.

### 3.4 Synthesis and Catalysis

The majority of the aforementioned research uses liposomes as a mere means to test transmembrane transport activity. However, it is also possible to see liposomes as useful entities in their own right, such as drug delivery vehicles (see section 2.5), artificial cells (see section 3.2), sensing devices (see section 3.3), or as “nanofactories” or “nanoreactors” capable of performing useful reactions. In nature, compartmentalization is used to spatiotemporally separate and control reaction pathways or incompatible reagents, and to create unique local chemical environments.^622^ For example, the membranes of the peroxisomes and lysosomes avoid that the harsh chemical conditions inside these organelles damage the rest of the cell. Furthermore, by placing enzymes responsible for metabolic cascade reactions in spatial proximity, more efficient multistep transformations can be achieved, that allow feedback mechanisms and the use of labile intermediates. It is therefore not surprising that researchers have tried to use liposomes and polymersomes (as well as more complex multilayered or liposome-in-liposome assemblies) as nanofactories for multistep reactions.^506,515,623,624^ In a more ambitious application of these nanoreactors, they have been incorporated into living cells as artificial organelles that can add new functions to the cells by providing new catalytic reactions, or for drug delivery purposes.^625^ However, as in nature, control over membrane permeability is important in these nanofactories and artificial organelles. In nature, transport proteins play an important part in metabolism by facilitating the import and export of metabolic substrates, cofactors and products, and by creating a suitable chemical environment (e.g., pH gradients).^626^ Synthetic nanofactories based on liposomes or polymersomes therefore either use membranes with intrinsic permeability or include pore-forming proteins in the membrane to facilitate these metabolic fluxes. For example, Nallani and co-workers prepared a multicompartment system consisting of polymersomes encapsulating horseradish peroxidase which themselves were enclosed by an outer semipermeable polymer membrane that contained glucose oxidase. Upon addition of glucose, glucose oxidase produces H_2_O_2_ which moves to the inner polymersome where it is used by horseradish peroxidase to generate a fluorophore (Figure 8).^627^ It was found that the addition of pore-forming protein OmpF in the internal polymersome membrane was essential for good conversion. While pore forming proteins are common, the use of artificial transporters and ionophores are rarely employed in this field. Recently, Langton and co-workers reported a liposome-system where a photoresponsive synthetic ionophore is able to transport Pd^II^ inside liposomes, where it is reduced to Pd^0^ (via ligand exchange) which then serves as a catalyst for a deallylation reaction.^628^ However, the use of synthetic anionophores in liposome- or polymersome-based nanoreactors has not yet been reported.

**Figure 8:**
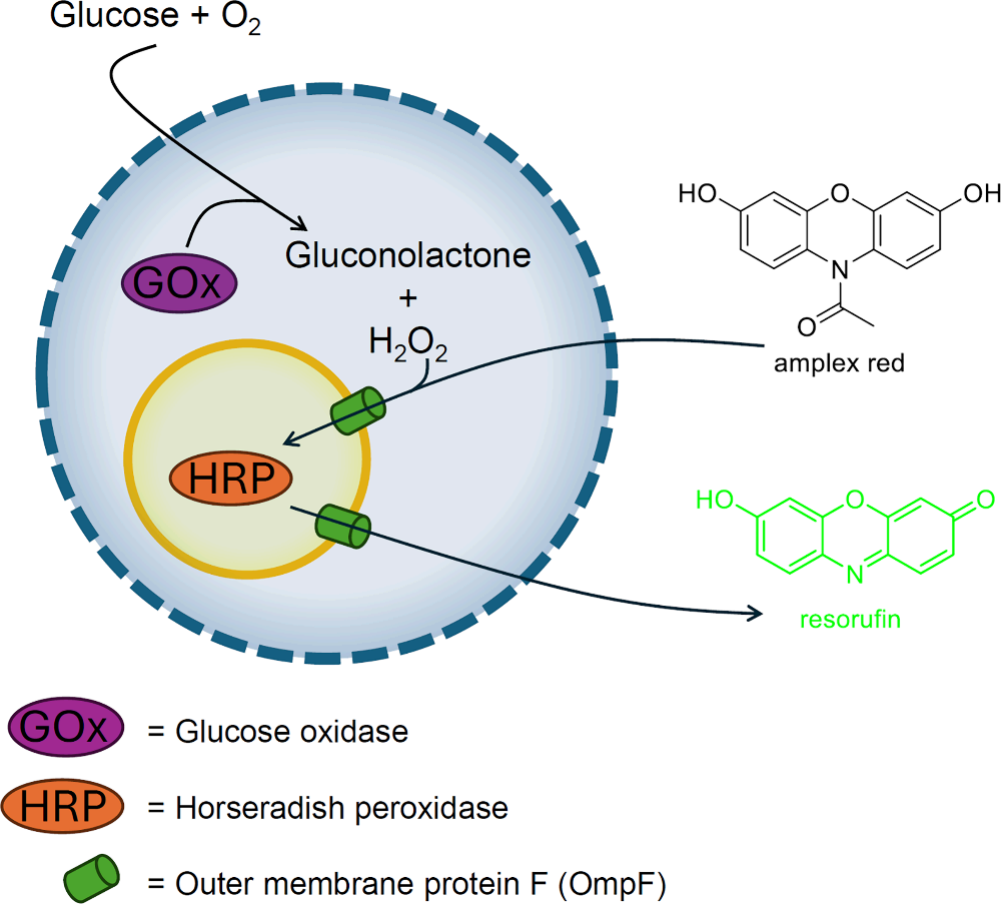
Principle of a polymersome-in-polymersome nanoreactor that uses the outer membrane protein F (OmpF) to allow flux of reagents and products throughout the system, reported by Nallani and co-workers. Adapted with permission from ref 627. Copyright 2014 Royal Society of Chemistry.

Another approach to combine transmembrane transport with synthesis is to study channels or mobile carriers that are themselves catalysts. For example, engineered mutants of α-hemolysin have been used to study catalytic cycles by taking advantage of the catalytic activity of the mutant protein combined with the single molecule detection ability of α-hemolysin in biosensors.^629–631^ The Matile group have also explored the combination of ion channel and catalytic activity with their synthetic rigid rod β-barrels (for an example, see Scheme 29).^632–636^ They found that their synthetic β-barrels with internal histidine–histidine, histidine–arginine, or histidine–lysine dyads are also capable of catalyzing ester and amide hydrolysis reactions (with *K*_M_ < 1 μM for some of the barrels), especially for anionic cascade blue-type substrates. They reasoned that the barrels might be able to transform the substrate as it is being transported through the pore. If this is the case, the resulting removal of the product by the transmembrane transport event should enhance the catalytic reaction. To test this, it was shown that catalysis can be enhanced when polarized liposomes are used, presumably because the inside-negative membrane potential drives the anionic substrate into the barrel and the subsequent anionic product out into the external solution (Figure 9).^634^ These results point toward a broader application of synthetic ion transporters in synthesis and catalysis, whereby the selective removal of products from the reaction via transmembrane transport events could potentially shift equilibria toward the products.

**Figure 9:**
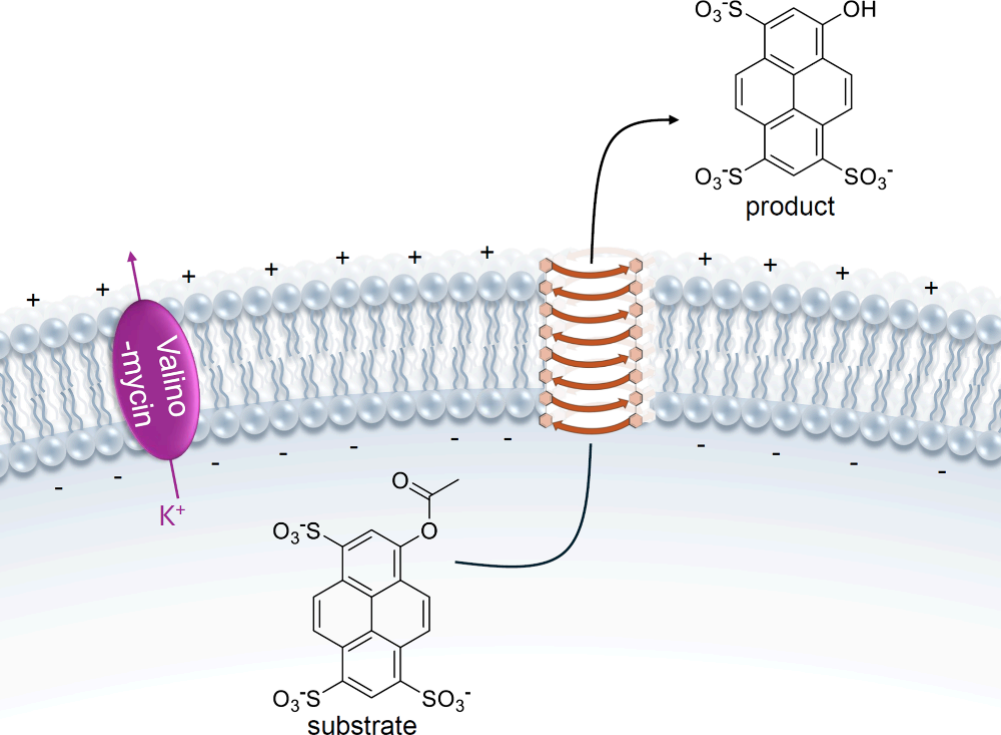
Principle of Matile’s multifunctional β-barrels with ion channel and catalytic activity. Liposomes are polarized by potassium gradients and the addition of the potassium carrier valinomycin. The created inside-negative membrane potential accelerates the transport of the substrate through the barrel, where it is also converted to the product via barrel-catalyzed ester hydrolysis.

More recently, Matile and co-workers got interested in halogen, chalcogen, pnictogen, and tetrel bonding for the development of synthetic anionophores.^637–639^ As these interactions have also been used in organocatalyst development,^640,641^ the Matile group thought to combine these two properties.^642^ They reported that stibine **139** functions as a potent transmembrane anion transporter, and can also catalyze the conversion of polyepoxide **140** into polyether **141** (Scheme 53). Because polyethers are common cation transporters, an interesting experiment was devised. When anion transporter/catalyst **139**, polyepoxide **140** and EYPC liposomes were mixed together, ion transport increased with increasing incubation times. Presumably, **139** can convert **140** into **141** and ion transport is then observed as cotransport mediated by anion transporter **139** and cation transporter **141**. It was found that under these optimized transport conditions, the conversion of **140** to **141** catalyzed by **139** could be completed in 1 h. As the same catalytic reaction in CD_2_Cl_2_ took multiple days to reach completion, a very large rate enhancement of >6 orders of magnitude is implied by the addition of liposomes and a transmembrane transport system. This enhancement could be the result of the high local concentrations achieved in the membrane, the essentially solvent-free conditions inside the membrane, and potentially a shift in the reaction mechanism. Whatever the reason, this example clearly shows the power of combining catalysis and ion transport in liposomes.

**Scheme 53:**
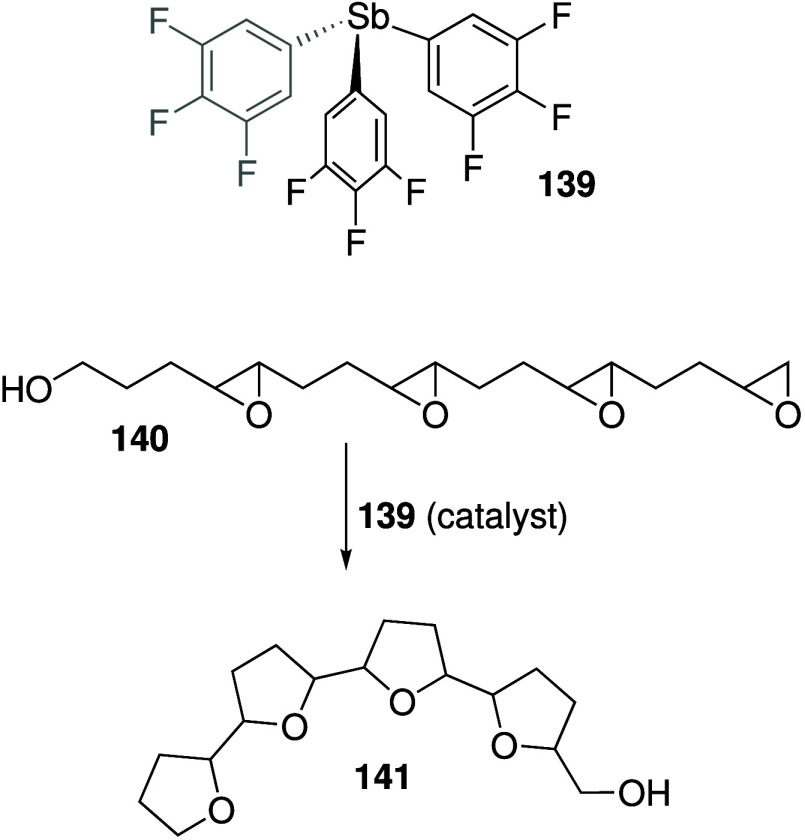
Structure of Anionophore **139** and the Reaction That It Can Catalyze (Converting Polyepoxide **140** into Polyether **141**)

Somewhat related to catalysis and synthesis is the development of artificial photosynthesis systems, which aim to convert photon (light) energy into chemical energy. As natural photosynthesis occurs through the action of a variety of transmembrane proteins, it comes as no surprise that membrane-based systems have been employed for artificial photosynthesis.^643–647^ In terms of using synthetic anion transporters for photosynthesis, an interesting report came from the Matile group.^648^ They reasoned that anionophores could be used to compensate the transmembrane electronic imbalance that occurs due to the photoinduced electron transport in photosynthetic systems. To demonstrate this, they reported the activity of oligo-(*p*-phenylene)-*N,N*-perylenediimide (PDI) rod **142** (Scheme 54). The PDI scaffold does not only have suitable photophysical properties for electron transport, but is also able to mediate transmembrane anion transport (e.g., chloride) via anion-π interactions. Photosynthetic activity was demonstrated in EYPC liposomes under continuous irradiation, with EDTA in the external solution as the electron donor and [Co­[bpy)_3_]^3+^ in the internal solution as electron acceptor.

**Scheme 54:**
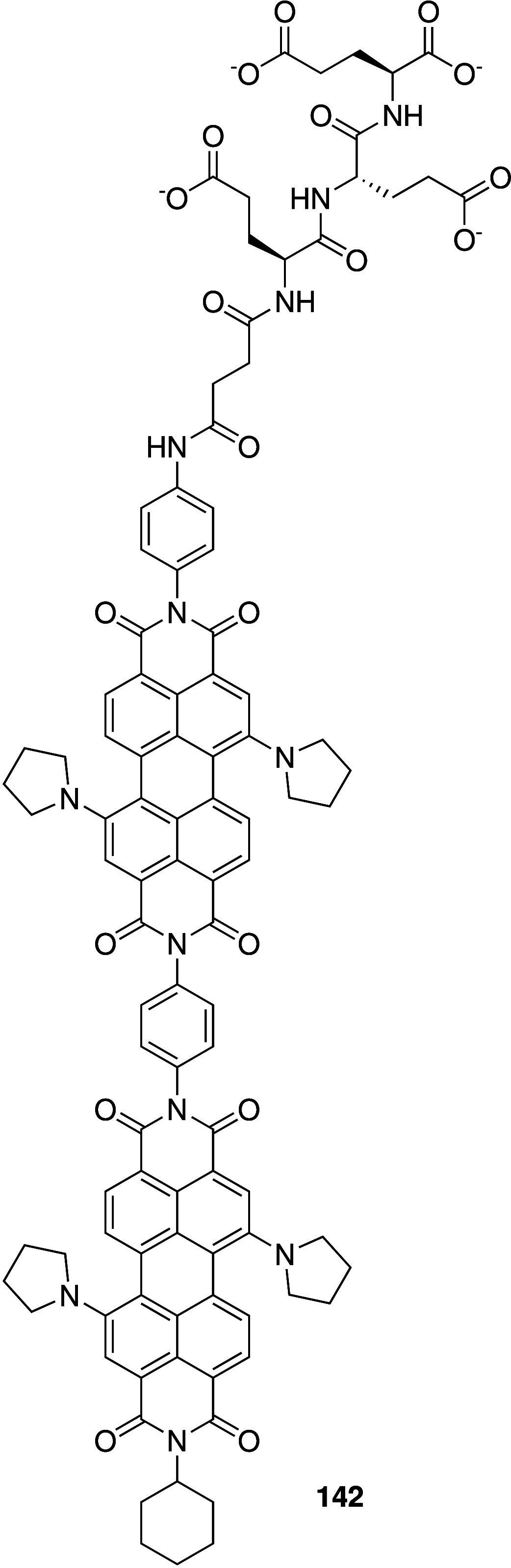
Structure of PDI Rod **142** with Photosynthetic Activity

### 3.5 Other Potential Applications

The previous sections have demonstrated that there is a plethora of potential applications for synthetic transmembrane anion transporters, ranging from the commonly studied cystic fibrosis and anticancer activity to the less explored sensing and catalysis systems. On top of this, we believe that there are even more applications that could be explored with synthetic anionophores and in this section we will speculate on some potential new avenues. Our aim is to encourage researchers to try out new applications for their anionophores, or to collaborate with researchers in other fields to test synthetic anionophores for other applications. For examples, ionophores are commonly used in ion selective electrodes and optodes, because these devices also rely on ionophores to transfer analytes from an aqueous solution to a membrane.^649–653^ In fact, the well-known cationophore valinomycin is commonly used in potassium selective electrodes.^654,655^ It therefore comes as no surprise that many of the research into anion selective electrodes employs similar chemical scaffolds as the anionophores covered in this review (e.g., bis- and tris­(thio)­ureas and squaramides).^656–662^ Collaborations between groups working on anionophores for medical applications with those working on ion selective electrodes (or optodes) could be mutually beneficial for both fields. Other inspiration can be found in the many applications that have been explored for natural ion channels and pore forming peptides or proteins, as well as synthetic cation channels. We have already mentioned the use of pore forming proteins in artificial cells (section 3.2), sensors (section 3.3) and catalysis (section 3.4), but other applications have also been suggested. Because ion channels essentially move charges from one place to another, they have been explored for electronic applications such as nanoelectronics and nanoionics.^663,664^ Natural membrane proteins have been also suggested for incorporation into polymer membranes, such as those used for nanofiltration (water purification) or for removal of pollutants.^665–668^ Furthermore, polymer membranes with nanometer-sized channels etched into them have been used as biomimetic systems with potential applications in materials science (usually by further functionalizing the etched channel/pore).^559,669,670^ It is therefore conceivable that synthetic anion channels and anion carriers could also find application in nanoelectronics, pollutant removal or materials science. It might be tempting to think that transmembrane anion transporters could be used for the liquid–liquid or liquid–solid extraction of anions (e.g., to remove toxic anions). However, efficient extraction benefits from ever increasing binding constants, while for transmembrane transport a double extraction is needed (first extraction of anions from the aqueous phase to the membrane and then again from the membrane to another aqueous layer) and binding constants cannot be too high. Transmembrane transport is therefore more related to catch-and-release applications than pure pollutant removal via extraction. Nevertheless, there is certainly potential to think of ways in which synthetic transmembrane anion transport could be used for pollutant removal applications. Finally, Gokel and co-workers tested the effect of their peptide-based synthetic anion transporters on the root growth of the plant *Arabidopsis thaliana.* ^671^ While most of the compounds had no effect on root growth, a few of them did. Interestingly, diphenylureas are known plant growth regulators (cytokinins)^672–674^ and have also been shown to function as transmembrane anion transporters (as mentioned a few times in this review). Studies of the effect of synthetic anionophores on plant growth could therefore still be interesting.

## Conclusions

In this review, we have mentioned the various applications that are possible for synthetic transmembrane anion transporters. The large variety of structures that have been shown to facilitate transmembrane anion transport has started to reveal some general design principles, such as the optimization of lipophilicity and binding strength (not too high and not too low for both, the so-called ‘Goldilocks’ effect). As a result, many compounds have started to be tested for potential real-world applications. While the field has mostly focused on the treatment of channelopathies and cancer, this review shows that there are many other possible applications. In recent years, there has been a growth in the number of publications mentioning the antibacterial properties of anionophores, and there is reason to believe that antifungal and antiviral applications are also possible. Furthermore, synthetic anion transporters can be used for drug or probe delivery, chemical biology, artificial cells, sensors and catalysis. For the field to move forward, we believe that researchers should start to standardize the assays that are employed to test activity (e.g., types of lipids used, mechanistic studies employed, EC_50_ values in mol % or μM, duration of experiments, ...). This will allow better comparison between compounds studied by different groups and will allow machine learning algorithms to be employed to find trends in the data and to predict new structures. We believe that this could greatly improve the field and move away from “potential” applications toward “real” applications.

## Abbreviations

POPC: (1-palmitoyl-2-oleoyl-glycero-3-phosphocholine)
EYPC: (egg yolk phosphatidylcholine)
IC_50_: (half-maximal inhibitory concentration–used to measure cytotoxicity)
MIC: (minimum inhibitory concentration–used to measure antibacterial activity)
EC_50_: (half-maximal effective concentration–used to report anion transport activity)
